# Neural anticipation of virtual infection triggers an immune response

**DOI:** 10.1038/s41593-025-02008-y

**Published:** 2025-07-28

**Authors:** Sara Trabanelli, Michel Akselrod, Julia Fellrath, Giulia Vanoni, Tommaso Bertoni, Silvia Serino, Georgia Papadopoulou, Maren Born, Matteo Girondini, Giuseppe Ercolano, Giulia Ellena, Anthony Cornu, Giulio Mastria, Hector Gallart-Ayala, Julijana Ivanisevic, Petr Grivaz, Maria Paola Paladino, Camilla Jandus, Andrea Serino

**Affiliations:** 1 Departement of Oncology, UNIL-CHUV, University of Lausanne, Epalinges, Switzerland; 2 Targeting of Cytokine Secreting Lymphocytes (TCSL) Laboratory, Department of Pathology and Immunology, Faculty of Medicine, University of Geneva, Geneva, Switzerland; 3 Ludwig Institute for Cancer Research, Lausanne Branch, Lausanne, Switzerland; 4 Geneva Centre for Inflammation Research (GCIR), Geneva, Switzerland; 5 Translational Research Centre in Onco-Hematology (CRTOH), Geneva, Switzerland; 6 MySpace Lab and NeuroRehab Research Center, Service of University Neurorehabilitation (SUN), Lausanne University Hospital, Institution of Lavigny and University of Lausanne, Switzerland, Switzerland; 7 Department of Psychology, Alma Mater Studiorum, University of Bologna, Bologna, Italy; 8 Metabolomics and Lipidomics Platform, Faculty of Biology and Medicine, University of Lausanne, Lausanne, Switzerland; 9 Department of Psychology and Cognitive Science, University of Trento, Trento, Italy; 10 Present Address: School of Health Sciences, HES-SO Valais-Wallis, Sion, Switzerland; 11 Present Address: Département Hospitalier, Institution de Lavigny, Lavigny, Switzerland; 12 Present Address: Translational Neural Engineering Lab, Neuro-X Institute, École Polytechnique Fédérale de Lausanne (EPFL), Lausanne, Switzerland; 13 Present Address: Department of Psychology, University of Milano-Bicocca, Milan, Italy; 14 Present Address: Department of Pharmacy, University of Naples Federico II, Naples, Italy; 15 Present Address: Center for Mind/Brain Sciences–CIMeC, University of Trento, Rovereto, Italy; 16 Present Address: Human Islet Isolation Facility, Nuffield Department of Surgical Sciences, University of Oxford, Oxford, UK; 17 Present Address: Nuffield Department of Clinical Neurosciences, John Radcliffe Hospital, Oxford, UK

**Keywords:** Perception, Innate immune cells

## Abstract

Once contact with a pathogen has occurred, it might be too late for the immune system to react. Here, we asked whether anticipatory neural responses might sense potential infections and signal to the immune system, priming it for a response. We show that potential contact with approaching infectious avatars, entering the peripersonal space in virtual reality, are anticipated by multisensory–motor areas and activate the salience network, as measured with psychophysics, electroencephalography and functional magnetic resonance imaging. This proactive neural anticipation instigates changes in both the frequency and activation of innate lymphoid cells, mirroring responses seen in actual infections. Alterations in connectivity patterns between infection-sensing brain regions and the hypothalamus, along with modulation of neural mediators, connect these effects to the hypothalamic–pituitary–adrenal axis. Neural network modeling recapitulates this neuro–immune cross-talk. These findings suggest an integrated neuro–immune reaction in humans toward infection threats, not solely following physical contact but already after breaching the functional boundary of body–environment interaction represented by the peripersonal space.

## Abstract (web summary)

Serino et al. show that seeing an infectious avatar approach the body in virtual reality triggers an immune response, indicating that the brain prepares the body to fight infections even for perceived, but not real, threats.

## Main

A vital function of an organism is to anticipate contact with threats to promptly activate a proper ‘fight-or-flight’ response. Mechanisms of predator detection and processing of threat imminence have been widely explored^1,2^. Pathogens represent special forms of threats that must be detected and avoided. Through evolution, social species developed a series of behavioral responses, such as social distancing, aimed at preventing contacts and thus infections that have been termed the ‘behavioral immune system’^3–6^. In primates, a mechanism that might be functional to predict contact with potential harm has been described within a network of fronto–parietal neurons, which integrate tactile stimuli on the body with external sensory information close to the body, that is, the peripersonal space (PPS) system^7,8^.

Once an external stimulus comes in contact with the body, another system reacts, that is, the immune system, composed of early- and late-acting arms (innate and adaptive immunity, respectively)^9^. A concerted effort of these two arms secures efficient pathogen clearance while preserving host tissue integrity^10^. Although reciprocal regulation of the neural and immune systems during actual disease is a research field in development^11–14^, there is no evidence of mutual interaction between the behavioral and biological immune systems that anticipates a concerted response to a potential infection before physical pathogen encounters.

Here, we asked whether the human brain is equipped with a mechanism of early sensing and anticipation of contact with virtual infections that is able to trigger a reaction of early players of the immune system, that is, innate lymphocytes (for example, innate lymphoid cells (ILCs) and natural killer (NK) cells) similar to when responding to contact with a physical pathogen. We exposed healthy participants to potential infection threats using virtual reality (VR) in the form of human faces displaying clear signs of infection (infectious avatars) and entering participants’ PPS. By using psychophysics, electroencephalography (EEG), functional magnetic resonance imaging (fMRI), mass spectrometry (MS) and flow cytometry, we measured behavioral, neural and immune responses to infectious avatars compared to responses evoked by control stimuli or actual contact with pathogens (that is, injection of a flu vaccine). Our results show that potential contact with virtual infection threats is predicted by fronto–parietal areas of the PPS system, activates the salience network and triggers a cascade of neuro–immune mediators, ultimately inducing changes in ILC frequency and activation.

## Results

### The PPS system anticipates contacts with infectious avatars

**Fig. 1:**
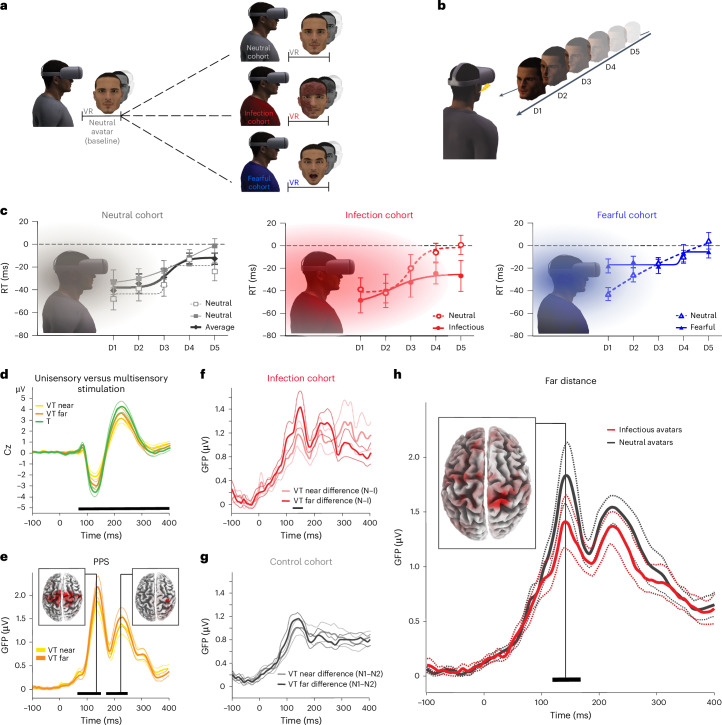
Early detection of infectious avatars by the PPS system. **a**, VR setup. Participants were first exposed to a neutral avatar (baseline) and then assigned to one of three cohorts encountering neutral, infectious or fearful avatars in a second session. **b**, PPS task. Participants responded to tactile stimuli on their face, delivered either in empty space (unisensory) or combined with an avatar approaching (multisensory). Tactile stimuli (flash icon) were presented at five delays, with the avatar located at one of five distances (D5 = far, D1 = near). **c**, PPS representation across cohorts. Reaction times (RTs) to multisensory stimuli were normalized by unisensory RTs (dotted line) and plotted by avatar distance. Shaded areas illustrate the PPS gradient, defined by significantly faster multisensory RTs (*P* < 0.05, corrected; paired-sample *t*-tests); *N* = 15 per cohort. Data are shown as mean ± s.e.m. (see Supplementary Table 3 for full statistics). **d**–**h**, EEG responses recorded from the central electrode (Cz) as an example (*N* = 16 per cohort). Black segments denote statistically significant time windows (cluster-based, nonparametric statistical tests; *P* < 0.01, corrected; solid lines = mean EEG response; dotted lines = 95% confidence interval). **d**, Evoked responses comparing unisensory (T) and multisensory (VT) stimulation as an index of multisensory processing. **e**, GFP analysis of PPS-related processing, that is, VT response differences for near versus far avatars. Insets show near–far current distribution differences at peak GFP effects. **f**, Significant GFP difference for near versus far stimulation between infectious and neutral avatars in the infection cohort. **g**, No GFP differences were found between two neutral avatars in the control cohort. **h**, Significant GFP differences between infectious and neutral avatars at the far location in the infection cohort, with the inset showing peak current distribution difference.

To trigger specific responses associated with virtual infection threats, we created a set of avatars showing clear signs of infection (infectious avatars) and two control conditions, namely neutral and fearful avatars (that is, an arousing but not pathogenic threatening stimulus; Fig. 1a). Results from explicit ratings, a seating distance scale^15^ and the Implicit Association Task^16^ demonstrated that infectious avatars were perceived as sick and contagious and evoked implicit avoidance responses compared to neutral and fearful avatars (Supplementary Table 1).

We then tested whether infectious avatars induced a specific response from the PPS system by applying a validated multisensory paradigm measuring the spatial extent of PPS at the behavioral level^6,17^. Participants were asked to respond as fast as possible to a tactile stimulation on their face while an avatar face was shown as approaching in immersive VR (Fig. 1b and Supplementary Table 2). Reaction times (RTs) to touch^18,19^ were measured when the approaching avatar was at five possible distances (D5–D1; multisensory RT) and normalized for RT when no avatar was presented (unisensory tactile (T) RT). The PPS extent was then indexed as the distance of the visual stimulus inducing a multisensory facilitation effect, that is, faster RT to multisensory than unisensory stimuli (hereafter the PPS effect).

To define a baseline for VR exposure, in the first session, all participants were exposed to neutral avatars, and in a second session, participants were divided into three cohorts and exposed to one of the three specific avatars, namely infection, neutral and fearful cohorts (Fig. 1a; the different cohorts were matched for participant sensitivity to disgust and anxiety; Supplementary Table 3). The results showed that the PPS effect varied between the baseline session and the second session as a function of the presented avatar (Fig. 1c). In the infection cohort, the PPS effect extended in the second session to cover the whole space between D1 and D4, whereas it was limited to D1 and D2 in the baseline session. The PPS effect did not vary between the two sessions in the neutral cohort and differed only at the closest position (D1) in the fearful cohort. Thus, infectious avatars specifically elicited a PPS effect at farther distances, indicating that the PPS system anticipates potential contacts with a virtual pathogenic threat when it is still far from the body.

### PPS brain areas sense infection threats early

We next searched for a neurophysiological marker of early detection of virtual infection compared to neutral stimuli entering the PPS. The visuo–tactile (VT) PPS paradigm was thus adapted to high-density EEG (128-channel EEG), in which participants were presented with either neutral or infectious avatars (infection cohort) or two different sets of neutral avatars (control cohort) and randomized in an event-related design. First, a cluster-based nonparametric statistical procedure was applied to determine a time window associated with VT multisensory processing (that is, in the presence of an avatar) compared to T processing only (Fig. 1d and Supplementary Fig. 1). Within this time window, we computed the Global Field Power (GFP^20^) as an index of evoked potential and compared GFP multisensory responses when a near or far avatar was presented as an index of PPS processing^21,22^. Results from both cohorts demonstrated a near–far difference arising from parietal and premotor areas, in line with previous neuroimaging studies^23,24^ (Fig. 1e), showing that the presence of an approaching avatar activates PPS-related brain areas.

We then tested whether this PPS effect differed when an infectious or neutral avatar was presented. In the infection cohort, the contrast [near (neutral – infectious) – far (neutral – infectious)] showed a significant GFP difference between 129 and 150 ms (Fig. 1f and Supplementary Figs. 2 and 3). To understand the origin of this effect, we compared GFP responses within this time window in the presence of an infectious or neutral avatar in the near or far space. We found a significant difference for far stimuli between the infectious and neutral avatars, whereas no difference was observed for near stimuli. In the control cohort, no difference was found between the two sets of neutral avatars (Fig. 1g). Thus, a virtual infection threat evoked a different neural response than a neutral stimulation, already when presented at a far location, in line with and extending the behavioral results presented earlier (Fig. 1c). Inverse solution analyses illustrated that the source of this difference was localized to parietal areas, which are part of the PPS system (Fig. 1h and Supplementary Tables 4 and 5). These electrophysiology findings demonstrate an anticipatory detection of virtual infections entering the PPS by multisensory–motor areas. To further localize these responses and clarify their directionality (see ‘Limitations of the study’ section), we conducted an fMRI experiment (see below).

### Infection threats modulate ILC function and activation

**Fig. 2:**
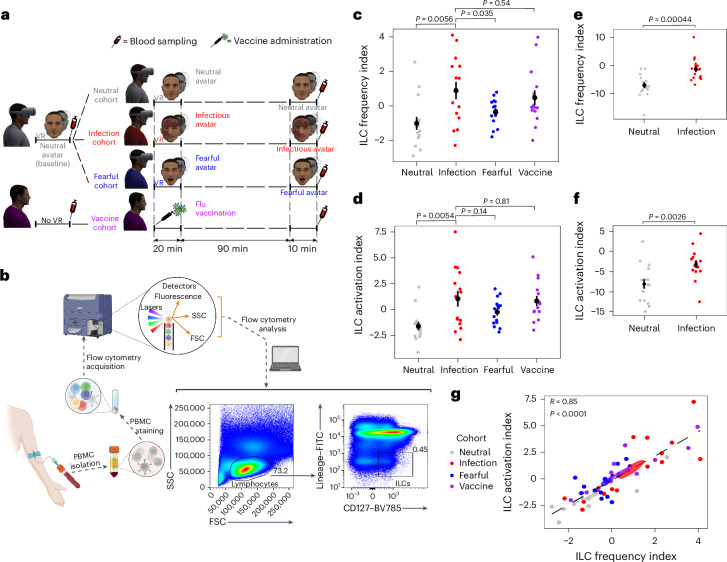
Modulation of ILC frequency and activation as a function of virtual or real stimulation. **a**, Study design for immunomonitoring. Participants were first exposed to neutral avatars (baseline). Blood samples were collected immediately after the baseline. Participants were then assigned to the neutral, infection or fearful cohort and exposed to the corresponding avatar condition (second session consisted of a 20-min cohort-specific VR stimulation, a 90-min break and a 10-min same cohort-specific VR stimulation). Blood samples were collected immediately at the end of the second VR session. In the vaccine cohort, blood samples were collected before and after flu vaccination (at the same time delay as for the VR cohorts, that is, with a 120-min time break between the first and second blood sampling). **b**, Scheme showing ILC identification from blood sampling. Peripheral blood mononuclear cells (PBMCs) were isolated, stained, and analyzed by flow cytometry. Figure created with BioRender.com. ILCs were identified as FCS^low^SSC^low^ lymphocytes that were negative for lineage markers and positive for CD127. **c**, ILC frequency was measured at the baseline and after the second session. The synthetic index of ILC frequency changes (first PCA component) in the four experimental cohorts is shown (analysis of variance (ANOVA): *F*_3,56_ = 4.71, *P* = 0.0053). **d**, Synthetic index of ILC activation changes (first PCA component) in the four cohorts (see Supplementary Fig. 8 for activation marker expression in each ILC subset; ANOVA: *F*_3,56_ = 5.45, *P* = 0.0023). **e**,**f**, Synthetic index of ILC frequency changes (**e**) and ILC activation changes (**f**) in two different independent cohorts (only neutral and infectious avatars were tested); *N* = 16 distinct participants per cohort (*P* values were derived from two-tailed *t*-tests). **g**, Correlation between synthetic ILC frequency and activation indexes, with different colors denoting the different cohorts (*R* = 0.85, *P* < 0.0001); shaded ellipses indicate the 66% confidence interval for the mean of each cohort. The black segmented line represents the linear regression. In **c**–**e** and **f**, data are presented as the difference between the values after the second VR exposure and the values at baseline. Black dots and lines represent the mean (dot) ± s.e.m. (line). *N* = 15 distinct participants per cohort (**c**, **d** and **g**).

We then tested whether virtual infections trigger an actual immune response. A new sample of participants was first exposed to neutral avatars, and then blood samples were taken immediately after the neutral VR stimulation to define an equivalent baseline. The participants were then divided into three cohorts (matched for sensitivity to disgust and anxiety; Supplementary Table 3), which were exposed to infectious, neutral or fearful avatars, and blood samples were taken after VR stimulation. To compare the immune response to virtual infection threats to that of a real pathogen, we tested a fourth cohort of participants who were not stimulated with VR but received an influenza vaccine (vaccine cohort), an ethically acceptable surrogate model for pathogen inoculation in humans (Fig. 2a). We assessed the early/antigen-independent phase of the lymphoid response by measuring the peripheral blood frequency and activation of innate lymphocytes, that is, ILCs and NK cells (Fig. 2b and Supplementary Fig. 4)^25–28^. To study a global index of the immune response, principal component analysis (PCA) was used on ILCs and NK cells (Supplementary Figs. 5–7 and Supplementary Table 6). Results revealed that virtual and real infections induced a similar stronger modulation of ILC frequency and activation indexes (evaluated as the differential expression of the activation markers CD25, CD27, CD69, NKp30, NKp44, NKp46, KLRG1, PD1 and HLA-DR) than neutral and fearful avatars (Fig. 2c,d and Supplementary Fig. 8). The main finding (that is, virtual avatars induce a stronger modulation of ILC frequency and activation indexes than neutral avatars) was further confirmed in an independent experiment (Fig. 2e,f).

ILC frequency and activation indexes showed a strong correlation and were both enhanced by virtual and real infections compared to the neutral cohort (Fig. 2g). No change was found in NK cell subset distribution and activation in response to real and virtual infections (Supplementary Figs. 9–13).

**Fig. 3:**
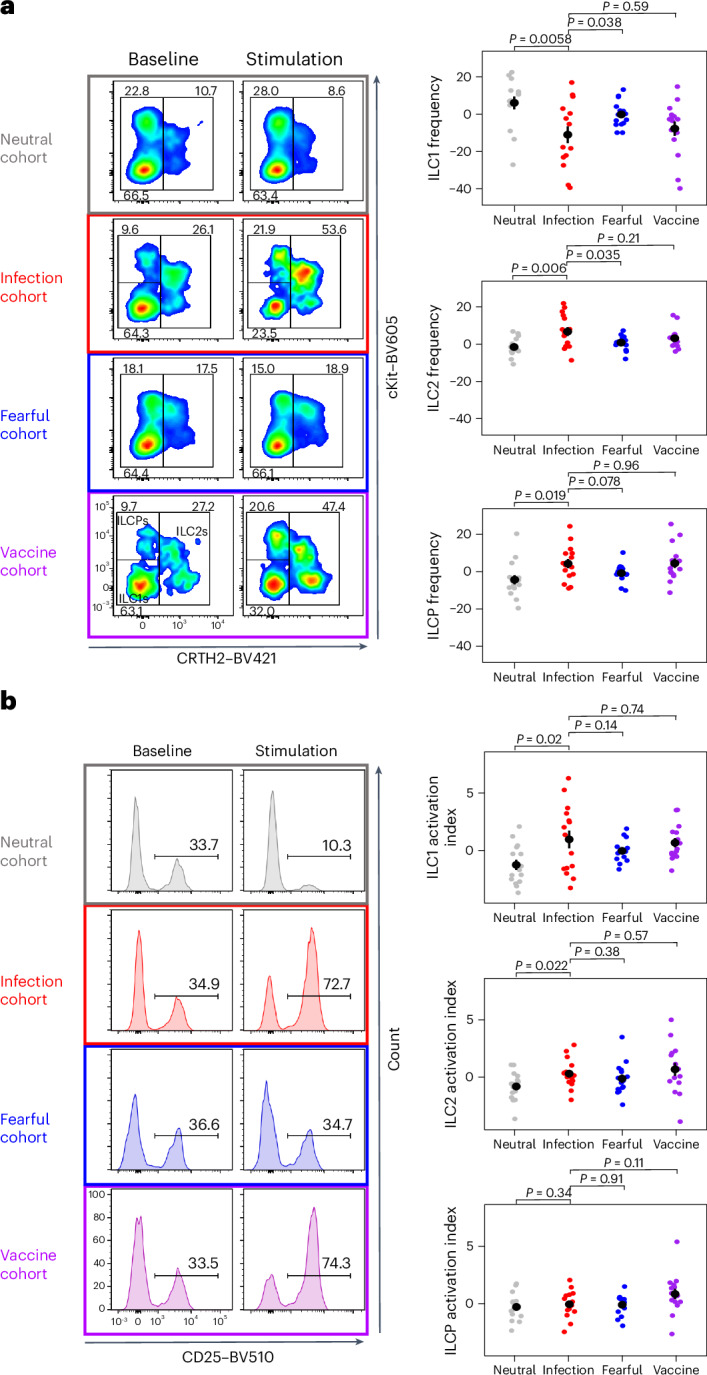
Modulation of ILC subsets as a function of virtual or real stimulation. **a**, ILC subset distribution. Left, representative dot plots of ILC1s identified as CRTH2^−^cKit^−^, ILC2s as CRTH2^+^cKit^+/−^ and ILCPs as CRTH2^−^cKit^+^ among total ILCs (that is, living lineage^−^CD127^+^ lymphocytes; see Fig. 2b) at baseline and after the second VR session. Right, changes in ILC1, ILC2 and ILCP frequencies among the different cohorts (ANOVA ILC1s: *F*_3,56_ = 4.69, *P* = 0.0054; ILC2s: *F*_3,56_ = 4.81, *P* = 0.0048; ILCPs: *F*_3,56_ = 3.65, *P* = 0.018). **b**, ILC activation by flow cytometry. Left, representative histograms of the percentage of CD25^+^ ILCs at baseline and after the second VR session as an illustrative example of ILC activation. Right, synthetic index of ILC1, ILC2 and ILCP activation changes (first PCA component) in the different cohorts (ANOVA ILC1s: *F*_3,56_ = 3.68, *P* = 0.017; ILC2s: *F*_3,56_ = 2.49, *P* = 0.07; ILCPs: *F*_3,56_ = 2.67, *P* = 0.056). In **a** and **b**, *N* = 15 distinct participants per cohort. Mean (dot) ± s.e.m. (line) is shown in black. Data are presented as the difference between the values after the second VR exposure and values at baseline.

Because the ILC family comprises three main subsets (excluding NK cells), namely ILC1s, ILC2s and ILC precursors (ILCPs), we analyzed the frequency and activation variations of each subset individually to understand which one contributed to the difference observed in the global ILC analysis. Virtual and real infections induced a similar strong decrease in ILC1s and increase in ILC2s and ILCPs (Fig. 3a), suggesting that all three subsets are responsible for the global changes observed. The reduction in ILC1 frequency was associated with a higher activation index in the virtual and real infection cohorts than in the neutral cohort (Fig. 3b). These results suggest that heightened ILC1 activation, concomitant with decreased ILC1 frequency (Supplementary Fig. 14), is likely a consequence of their activation-induced migration from the blood to tissues, consistent with their involvement in early antiviral tissue responses^29^. Together, these data show that ILCs react to infections not only when they are detected in the body but also when they are processed as a potential threat approaching the body.

### Brain regions encoding virtual infection threats

**Fig. 4:**
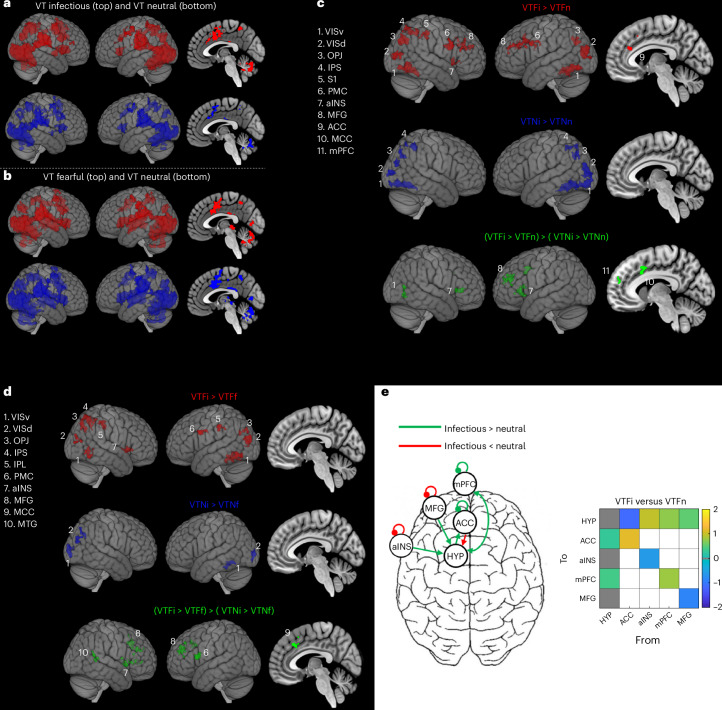
fMRI activations and hypothalamic connectivity in response to infectious, fearful and neutral avatars. **a**, In the infection cohort, contrasts (red: VTFi + VTNi; blue: VTFn + VTNn) revealed VT activations specific to each face type. Activated regions included the bilateral superior/inferior parietal lobules (SPL and IPL, respectively), S1, supplementary motor area (SMA), middle cingulate cortex (MCC), temporo–parietal junction (TPJ), face-sensitive visual areas (occipital, fusiform and lingual gyri), cerebellum and insula. **b**, Similar activation patterns were observed for the fearful cohort using equivalent contrasts (*N* = 20). **c**, Infection-specific activations (infectious > neutral) varied by avatar proximity as shown for far space (red), near space (blue) and their direct contrast (green). **d**, Comparison between infectious and fearful avatars (versus neutral) for far (red) and near (blue) space and their direct contrast (green); *N* = 38. **e**, DCM revealed modulated hypothalamic connectivity during exposure to infectious avatars. Increased input: from mPFC and aINS. Decreased input: from MFG and ACC. Significant connections (posterior probability > 0.95) to/from the hypothalamus are shown in the connectivity matrix. fMRI activations were thresholded at cluster-level family-wise error correction, *P* < 0.05; i, infection; n, neutral; F, far; N, near; VISv, ventral visual areas; VISd, dorsal visual areas; OPJ, occipito–parietal junction; IPS, intraparietal sulcus; PMC, premotor cortex; MTG, middle temporal gyrus; HYP, hypothalamus. In **a**, **c** and **e**, *N* = 18 participants.

To gather deeper insight into the localization and response pattern of the brain network detecting infectious avatars, we conducted an fMRI experiment by adapting the PPS paradigm to an fMRI-compatible VR setup. Within the same participants, we compared brain activation induced by tactile stimuli associated with either infectious or neutral avatars, presented in far space (that is, where infectious avatars evoked specific behavioral and electrophysiological effects) or in the near space. To disentangle specific brain processing responsible for the detection of infection threats from responses to a generic environmental threat, another group of participants was presented with either fearful or neutral avatars, with the same design. We first identified multisensory processing from a distributed bilateral fronto–parieto–occipital network (Fig. 4a,b and Supplementary Fig. 15), including key nodes of the PPS system^24^ in the two groups. To test whether the activation of the PPS system, when participants faced a far infectious avatar, reflected anticipatory processing, we contrasted the blood oxygen level-dependent (BOLD) responses to tactile stimuli coupled with infectious versus neutral avatars in the far and near spaces. Activations (infectious > neutral) in the intraparietal sulcus and visual areas were common to both far and near conditions. In turn, activations in the right primary somatosensory cortex (S1), right anterior insula (aINS), bilateral premotor cortex, bilateral anterior cingulate cortex (ACC) and bilateral middle frontal gyrus (MFG) were specific for the far conditions (Fig. 4c). The direct contrast between responses to infectious versus neutral avatars in the far compared to the near space highlighted additional specific activations in the left medial prefrontal cortex (mPFC) and left aINS (Fig. 4c and Supplementary Table 7). These regions are part of the so-called salience network, that is, an ensemble of interconnected brain regions whose major role is detecting and filtering salient stimuli, including threats^30^, to recruit relevant functional networks implementing proper reactions. Some of the areas activated in our task (intraparietal sulcus, ACC and MFG) have been reported to be triggered during a cognitive task performed under an inflammatory state induced by a vaccine^31^. Here, we show that the PPS network and the salience network respond to virtual infections to implement fast responses. Importantly, this pattern of brain activations was specific to detection of virtual infection, as they also emerged when we directly compared activity induced by infection and fearful avatars (after subtracting the response to neutral avatars; Fig. 4d and Supplementary Table 8). We then studied how this brain mechanism of infection threat detection might project to the immune system to trigger an immune response, as the ILC reaction shown above.

### Infectious avatars change cortex–hypothalamus connectivity

Behavioral responses to threats are described in terms of ‘fight-or-flight’, and their neural implementation has been characterized in rodents within a network of areas including the frontal cortex, amygdala, hypothalamus and periaqueductal gray^32^. The lateral hypothalamus is also a key area in the regulation of innate immune responses by the central nervous system, which could mainly occur by direct release of catecholamines via the sympathetic nervous system and by corticosteroids via the hypothalamic–pituitary–adrenal cortex (HPA) axis^11,33^. These responses might be further modulated by a number of signaling factors, including eicosanoids and neuroinflammatory mediators^34,35^.

Thus, we first searched for involvement of the HPA axis in responding to virtual infection. We used dynamic causal modeling (DCM) analyses to reveal stimuli-specific changes in connectivity between the hypothalamus and key areas of the PPS system and the salience network being activated by infectious avatars compared to neutral or fearful avatars. Results showed different modulation of hypothalamic connectivity when infectious avatars were presented in the far space compared to neutral avatars. In particular, far infectious avatars were associated with connectivity upregulation between the hypothalamus and the aINS, MFG, mPFC in the left hemisphere and S1 in the right hemisphere (Fig. 4e and Supplementary Fig. 16). No modulation was found from the same analyses for conditions with avatars in the near space nor in the fearful avatar group. Thus, fMRI connectivity results point to a modulation of hypothalamus activity via the salience network in response to virtual infections anticipated by the PPS system that might be the upstream node of the neuro–immune pathway triggering the systemic innate immune response.

### Infectious avatars activate a specific neuro–immune axis

**Fig. 5:**
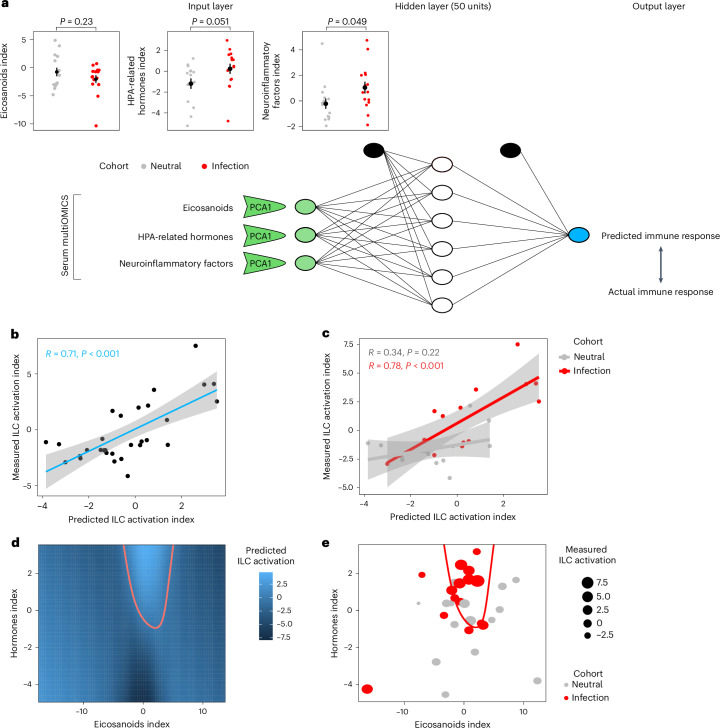
Neural network modeling of immune signaling. **a**, Network architecture and testing. We trained a single hidden layer neural network using the first PCA component of eicosanoids (neutral versus infectious; *t*(28) = 1.25, *P* = 0.23), HPA-related hormones (neutral versus infectious; *t*(28) = 2.04, *P* = 0.051) and neuroinflammatory factors (neutral versus infectious; *t*(28) = 2.06, *P* = 0.049) as input and the ILC activation index as an output on all participants from the neutral and infection cohorts. To compare network predictions with actual ILC activation, we used leave-one-out cross-validation; *N* = 15 distinct participants per cohort. Data are shown as mean ± s.e.m. *P* values of two-sided two-sample *t*-tests (neutral versus infectious) are reported. **b**,**c**, Comparison between predicted (*x* axis) and measured (*y* axis) ILC activation indexes in all participants (*P* = 0.000011; **b**) and differentiation between the infection and neutral cohorts in red (*P* = 0.000604) and gray (*P* = 0.215002; **c**), respectively. Linear regressions were performed (shaded area = 95% confidence interval); *N* = 15 distinct participants per cohort. **d**, Predicted ILC activation as a function of HPA-related hormones and eicosanoids. The network’s input–output relation in two dimensions is shown from training a network on the whole population, while the neuroinflammation level was fixed at its mean value. The red line denotes the activation hot spot, defined as the region in which the predicted immune response is larger than the average. **e**, Measured eicosanoids and HPA-related hormones in participants from the infection (red dots) and neutral (gray dots) cohorts (with neuroinflammation input fixed at its average); *N* = 15 distinct participants per cohort. Most participants in the infection cohort fall within the predicted activation hot spot (*P* = 0.008, two-sided *χ*^2^ test) indicated by the red line.

Previous data showed that ILC functions are modulated by HPA-related hormones in models of endotoxin-induced systemic inflammation^36^. In line with these observations, ILC modulation by infectious avatars might be detected on a cascade of effects along the HPA axis. Thus, we performed MS quantification of a set of HPA-related hormones in the serum of individuals in the infection and neutral cohorts (Supplementary Table 9 and Supplementary Fig. 17)^37–40^. In addition to these hormones, other factors may intervene in neuro–immune cross-talk. Brain- and systemic-derived molecules, such as catecholamines and nonsteroidal metabolites of the arachidonic acid pathway, are largely involved in inflammatory responses, leukocyte chemotaxis, temperature and blood pressure regulation during infection^34,35,41^. The half-life of catecholamines in the blood is extremely short (1–2 min), rendering their measurement impossible in the current setting. Thus, we quantified eicosanoids as arachidonic acid metabolites (Supplementary Table 10) and neuroinflammatory factors (Supplementary Figs. 18 and 19) to study the cross-talk between neural signaling and immune responses triggered by infectious compared to neutral avatars. We first applied a data reduction approach to neural mediators by running three independent PCAs on HPA-related hormones, eicosanoids and neuroinflammatory factors (Fig. 5a). We extracted the first component of each PCA (explaining 39, 36 and 33% of the variance, respectively; Supplementary Figs. 20 and 21) as a synthetic index of each pathway, and we used it to predict the pattern of elicited immune response as captured from the ILC activation index. Univariate or multivariate linear regressions between neural signals and immune activation revealed no conclusive pattern, suggesting that the neuro–immune cross-talk could not be explained by simple relationships. Thus, we applied a machine learning-based approach, whereby the three synthetic indices of neuro–immune signaling were used to predict ILC activation in a single hidden layer neural network (Fig. 5a).

We tested the relationship between the predicted activation index and the empirical data by using leave-one-out cross-validation. The neural network was able to explain 71% of the variance in the empirical data by uncovering multivariate, nonlinear relationships between neural signaling and immune activation, with a stronger prediction power for the infection cohort, even if information about the cohort was not used for training (Fig. 5b,c). To interpret such complex, multidimensional relationships in the neuro–immune cross-talk, we mapped the predicted immune response as a function of the level of HPA-related hormones, eicosanoids and neuroinflammatory factors. We found that ILC activation increased almost linearly with HPA-related hormone levels and decreased almost linearly with neuroinflammatory factor levels. ILC activation was facilitated when eicosanoid levels were within a specific level, resulting in a nonlinear, Gaussian-shaped response profile. As a result, the highest predicted immune response was found in a ‘hot spot’ of high HPA-related hormones, low neuroinflammatory factors and intermediate eicosanoid levels (Fig. 5d and Supplementary Figs. 25–28). As an empirical confirmation of such predicted effects, we found that actual data from participants from the infection cohort had a significantly higher probability than participants from the neutral cohort to fall into the hot spot predicted by the neural network (Fig. 5e). The same neural network trained on the neutral and fearful groups did not reveal significant relationships between neuro–immune signaling and ILC response (Supplementary Fig. 22). The network also failed when any of its three inputs was removed, suggesting that all three families of signaling factors are essential to drive the immune response. Together, these analyses suggest that a virtual infection threat (and not a generic threat) induces a specific pattern of neuro–immune signaling, which is sufficient to drive ILC activation.

## Discussion

Our integrated behavioral, neurophysiological, immunological and computational analyses provide a direct demonstration that potential infection threats (even when presented in VR) are processed by the PPS system and the salience network in an anticipatory way and preactivate the immune system by triggering ILC responses, likely via a nonlinear neuro–immune cross-talk involving the HPA axis. An important line of work describes the neural mechanisms allowing animals to detect threats and select appropriate behaviors^1,7,8,42^. Our results extend this framework to the response of the immune system to virtual infection threats. One key mechanism to defend oneself from infection is by early detection of potential threatening encounters to promptly decide whether to engage in ‘fight-or-flight’. Therefore, anticipation can result in a pathogen avoidance strategy, for example, by adopting social distancing behaviors as for the recent coronavirus disease 2019 (severe acute respiratory syndrome coronavirus 2) pandemic^6,43–45^, or in a pathogen defense approach, by activating immune cells to destroy the infection threat. Thus, in the absence of a consolidated avoidance policy, the findings from the infection cohort suggest that our immune system adopts fight anticipatory strategies, allowing our organism to react to immune threats not only once they are in the body but also when they overcome the primary functional boundary of self-environment interaction represented by the PPS. These results extend the spectrum of responses of the behavioral immune system from adapting social behavior^4^ to driving innate immune responses of effectors and counter-regulatory loops. Although surprising, our finding that immune responses can be triggered by simulated infections presented in VR is consistent with the principle of the smoke detector in biological systems^46^. Accordingly, the behavioral immune system evolved to minimize false-negative responses and is exquisitely sensitive to cues that superficially resemble the symptoms and signs of pathogen infections. In this study, VR proved to efficiently deliver such cues, demonstrating its potential as a valuable approach to further dissect the vital and complex link between the central nervous system and the immune system.

### Limitations of the study

First, given the lack of prior research on immune responses to virtual brain stimulation in humans, this study is exploratory. Second, the selection of immune markers, timing of virtual and real (vaccine) pathogen exposures, neuroinflammatory factors and use of machine learning were guided by current scientific knowledge and available tools. Third, the generalizability of the results requires further studies because ILC changes were only compared to one vaccine (FluarixTetra 2018–2019, used in the 2018 Swiss anti-influenza campaign). Additionally, as immune and PPS systems vary with age^47–49^, our focus on young adults limits the applicability to other age groups. Furthermore, we used a well-established paradigm with looming stimuli to probe PPS responses^18,19^, but whether static images elicit similar immune effects remain unknown and requires future research. Finally, the EEG results show different responses to infectious and neutral avatars from PPS-related areas, especially in the far space, that is, at the PPS boundary for infection stimuli. However, the direction of the GFP modulation is not fully consistent with the electrophysiology literature on PPS representation (normally showing higher responses for PPS-related stimuli). This might depend on the use of looming stimuli, compared to static stimuli used in previous studies^21–23^, of VT stimuli rather than audio–tactile stimuli and on the sampled spatial distances. These factors might affect the temporal dynamics of the electrophysiological response. Future studies should better clarify this point. In the present study, the fMRI results better allowed us to interpret the directionality of brain responses to infectious avatars.

A theoretical limitation in the field is the potential confound between perceived infectiousness and disgust. Disgust is central to the behavioral immune system and helps avoid infectious threats^50^. Infectious-looking avatars may trigger more disgust than neutral ones, but this factor cannot be fully separated from contamination-avoidance mechanisms. We addressed this by (1) ensuring that our cohorts were matched for disgust sensitivity (Supplementary Table 3) and (2) showing that including disgust as a covariate in fMRI analyses did not affect the key results (Supplementary Fig. 23).

## Methods

### Study

This research complies with all relevant ethical regulations. The experiments were conducted in accordance with the principles of the 1964 Declaration of Helsinki and were approved by the ethical committee ‘Commission cantonale d‘éthique de la recherche sur l‘être humain’ of the Vaud and Geneva cantons, Switzerland (201701588).

### Participants

A total of 248 individuals participated in the study across the following five different experiments: (1) attitude and arousal toward avatars (*n* = 41), (2) behavior (*n* = 45), (3) EEG (*n* = 32), (4) immune response (*n* = 60 + 32) and (5) fMRI (*n* = 38). The different experimental cohorts were age and sex matched (total of 132 women; mean age = 26.8 years, range 18–49). Within each experiment, the different experimental cohorts (infectious, fearful and neutral) were tested for differences in sensitivity to disgust and anxiety, and no difference was found (Supplementary Table 1). All participants were recruited for a single experiment through the participant management software ‘Sona-Systems’.

Participants were right-handed, reported no history of neurological, psychiatric or immune disorders, had no somatosensory impairments and had normal or corrected-to-normal vision. None reported specific diseases making the acquisition of blood samples unsafe (for example, hemophilia and anemia). The vaccine cohort was vaccinated with FluarixTetra 2018–2019.

For all participants, the experiment was run at the same time in the morning (from 8:30 a.m. to 12:30 p.m.). Participants were financially reimbursed for their time (20 Swiss Francs per h), and all provided written informed consent before participating.

### Visual stimuli

Visual stimuli were presented stereoscopically with a head-mounted display (Oculus Rift CV1, 110° field of view, resolution 2,160 × 1,200, refresh rate of 90 Hz). All visual stimuli consisted of female or male virtual faces with different facial features that were sex matched to the participants. Avatar faces could present a neutral expression, a fearful expression or signs of infectious disease and sickness. All avatars were created with ‘Poser 10 Pro’ software (posersoftware.com) and were postprocessed using Blender (www.blender.org). Neutral and fearful avatars were selected from previous validated literature^51^. For infectious avatars, a validation procedure consisted of an initial database of 22 virtual faces (11 men and 11 women), each showing signs of sickness (Supplementary Methods 1 and 2).

### Tactile stimuli

Tactile stimulation was delivered using a pair of cylindrical mechanical vibrators (Precision Microdrives, Pico Vibe Vibration Motor, diameter of 10 mm, stimulation frequency of 100 Hz, stimulation duration of 200 ms) taped to the cheeks of the participants and interfaced using a custom-made programmable microcontroller, which also sampled at 10 kHz the response times provided by a hand-held press button. For the fMRI experiment, a magnetic resonance-compatible pneumatic stimulator was used instead of non-magnetic resonance-compatible mechanical vibrators.

### Behavioral responses to virtual infectious threats entering the PPS

To measure whether infectious avatars affected PPS representation, we adapted a well-validated multisensory interaction task previously used to measure the extent of the PPS behaviorally^17–19^. In this task, participants are required to press a button as fast as possible after receiving an automatized mild touch to their face, while concurrently observing a task-irrelevant visual stimulus looming toward their face. In different trials, touch is delivered when the visual stimulus is perceived at a different distance from the participant, and RT is measured to derive a proxy of the extent of PPS (Supplementary Method 3). Custom Python (v3.7) code was used to present the VR stimuli, based on the ExpyVR toolbox (v1.0; https://www.epfl.ch/labs/lnco/research/expyvr/)

### EEG analyses (sensor space)

EEG data were acquired using a 128-channel system (ActiveTwo, Biosemi V.O.F.). The EEG paradigm, acquisition and preprocessing (EEGLAB v14 with MATLAB v2017a and Cartool v3.12) are described in Supplementary Method 4 and Supplementary Fig. 24. Statistical analyses were performed in two steps. First, we identified time windows responding to multisensory (VT) stimuli (versus T). Then, within these time windows, we characterized distinct PPS responses for neutral and infectious avatars. In the first step, we compared multisensory (VT near and VT far) to unisensory (T) responses (Fig. 1d). To increase the power of this analysis, for each distance (near and far), we combined EEG data of condition 1 (neutral avatars) and condition 2 (neutral avatars in the control cohort and infectious avatars in the infection cohort) of both cohorts (control and infection). Significant differences between T and VT (near and far) were determined with a cluster-based, nonparametric statistical procedure as implemented in the Fieldtrip toolbox (v20171231 with MATLAB v2017a)^52,53^. This data-driven approach controls for the false-positive error rate in a situation of multiple comparisons (multiple time points and electrodes). Significant time windows (between −100 ms and 400 ms) in the contrast between VT and T were considered as multisensory responses and were selected for the second step of the analyses. In the second step, we conducted analyses based on the classic approach to study PPS^22,23,54^, in which PPS is defined as a multisensory modulation of tactile stimulation due to an external stimulus (here visual presentation of avatars), as a function of the distance of these stimuli from the body in space. We first estimated the PPS distance effect (VT near versus VT far) with EEG data from both cohorts together (Fig. 1e). We then tested whether the PPS response was distinct when a neutral or infectious avatar was presented by using the contrast [near (neutral – infectious) – far (neutral – infectious)] in the infectious cohort and [near (neutral – neutral 2) – far (neutral – neutral 2)] in the control cohort (Fig. 1f–h). All statistical analyses in the second step were performed on GFP. GFP has the advantage of representing a measure of the neural strength of evoked responses while reducing the inherent high dimensionality of EEG data (false positive).

### EEG analyses (source space)

To localize neural activity in key contrasts, we performed a current density analysis in three-dimensional Tailarach/MNI space of scalp-recorded electrical activity using the sLORETA/eLORETA software package^55^. sLORETA estimates the distribution of electrical neural activity in three-dimensional space based on the measurements of a dense grid of 6,239 voxels at 5-mm spatial resolution, which are placed on the entire scalp surface covering the brain. This inverse solution algorithm assumes related orientations and strengths of neighboring neuronal sources (represented by adjacent voxels). Figure 1e,h shows the difference in estimated current distribution at the time point showing the strongest statistical difference in GFP within each significant time window.

### PBMC and serum isolation

Venous blood was drawn from 60 healthy donors immediately after the baseline session (consisting of a 20-min VR stimulation with neutral avatars or following arrival of the participants assigned to the vaccine cohort) and after the second session (consisting of two VR stimulations accordingly to the assigned cohort). The second session consisted of a 20-min VR exposure, 90-min break and another 10-min VR exposure. Thirty individuals were randomly assigned to the neutral and infection cohorts (*n* = 15 per cohort), with an equal number of tested participants per condition per day. Two independent cohorts were further enrolled and assigned to the fearful or vaccine cohort. Participants assigned to the vaccine cohort waited for the same time delay as participants exposed to VR. Therefore, the time interval between the blood samplings was 120 min in all cohorts.

To replicate the main findings, a new independent sample of participants (*n* = 16 per cohort, see Statistics for the sample size calculation) was recruited and randomly assigned to the neutral or infection cohort. For comparisons between the neutral and infection conditions, participant cohort assignment was not communicated to the immunologists analyzing blood samples to ensure blindness in the analysis.

PBMCs were isolated by Lymphoprep centrifugation (600*g*, 20-min centrifugation without break, room temperature) and washed twice with PBS. Platelets were removed by centrifugation (200*g*, 10-min centrifugation with break, room temperature), and red blood cells were eliminated by incubating the cell pellets with 1 ml of red blood cell lysis buffer for 5 min at 37 °C. For serum collection, whole blood was centrifuged (1,800*g*, 10 min, room temperature). Following centrifugation, the serum was collected and immediately cryopreserved at −80 °C.

### PBMC analysis

Isolated PBMCs were immediately stained for 20 min at room temperature in sorting buffer (PBS, 50 μM EDTA and 0.2% bovine serum albumin) with the following FITC-conjugated lineage markers: anti-human CD3 (UCHT1, Beckman Coulter (BC), 1:200), anti-human CD4 (SFCI12T4D11, BC, 1:200), anti-human CD8 (MEM-31, Immunotools, 1:100), anti-human CD14 (RMO52, BC, 1:200), anti-human CD15 (80H5, BC, 1:50), anti-human CD19 (J3-119, BC, 1:100), anti-human CD20 (2H7, Biolegend, 1:400), anti-human CD33 (HIM3-4, Biolegend, 1:400), anti-human CD34 (561, Biolegend, 1:100), anti-human CD203c (E-NPP3, Biolegend, 1:25) and anti-human FcεRIα (AER-37, Biolegend, 1:200). Additionally, we used APC/Cy7 anti-human CD27 (M-T271, Biolegend, 1:50), Brilliant Violet 605 anti-human CD117 (cKit) (104D2, Biolegend, 1:200), Brilliant Violet 421 anti-human CRTH2 (CD294; BM16, Biolegend, 1:200), PerCP/Cy5.5 anti-human CD335 (NKp46; 9E2, Biolegend, 1:50), PE anti-human CD337 (NKp30; P30-15, Biolegend, 1:100), PE/Dazzle 594 anti-human HLA-DR (L243, Biolegend, 1:200), PE/Cy7 anti-human KLRG1 (14C2A07, Biolegend, 1:200), APC anti-human CD336 (NKp44; P44-8, Biolegend, 1:100), Alexa Fluor 700 anti-human CD16 (3G8, Biolegend, 1:100), Brilliant Violet 510 anti-human CD25 (BC96, Biolegend, 1:100), Brilliant Violet 650 anti-human CD69 (FN50, Biolegend, 1:200), Brilliant Violet 711 anti-human CD279 (PD1; NAT105, Biolegend, 1:50), Brilliant Violet 785 anti-human CD127 (IL-7Rα; A019D5, Biolegend, 1:200) and BUV737 anti-human CD56 (NCAM16.2, BD, 1:100). Dead cells were excluded using the viability dye DAPI (Invitrogen, 1:10,000). Samples were acquired on an LSR SORP flow cytometer (BD using the BD FACSDiva software v8.0.2), and data were analyzed using FlowJo software v10.7.1. For the replication experiment, samples were acquired on an LSR Fortessa flow cytometer (BD using the BD FACSDiva software v8.0.2), and data were analyzed using FlowJo software v10.8.1 (TreeStar). This combination of markers allowed us to identify NK^bright^ cells as living lineage^−^CD16^−^CD56^bright^ lymphocytes, NK^dim^ cells as living lineage^−^CD16^+^CD56^dim^ lymphocytes and total ILCs as living lineage^−^CD16^−^CD56^−^CD127^+^ lymphocytes. ILCs were further divided into CRTH2^−^cKit^−^ ILC1s, CRTH2^+^cKit^+/−^ ILC2s and CRTH2^−^cKit^+^ ILCPs. The other markers were used to assess the activation status of NK cells and ILCs and were analyzed and plotted using GraphPad Prism version 10.4.1 (Supplementary Figs. 8, 11 and 13).

### fMRI data acquisition and processing

All data were acquired on a Siemens Prisma 3T magnetic resonance scanner with a 32-channel receiver/transmitter head coil (see Supplementary Method 5 for fMRI adaptation of the PPS task). Functional volumes were acquired using a gradient echo planar imaging sequence over the whole brain (TR: 1,000 ms; TE: 32 ms; slice thickness: 2 mm; 66 axial slices; in-plane resolution: 2 × 2 mm^2^; multislice acceleration factor: 6). Four functional runs were acquired with the presentation of the experimental conditions, each with 380 volumes. Furthermore, T1-weighted structural images (mprage sequence, sagittal orientation, resolution: 1 × 1 × 1 mm^3^, TR: 2,000 ms, TE: 2.25 ms, flip angle: 8°) were recorded after the acquisition of functional images.

All images were preprocessed using SPM12 software (with MATLAB v2021a) (Wellcome Department of Cognitive Neurology). Preprocessing steps included slice timing correction, realignment, minimal smoothing (full-width at half-maximum = 3 mm) and normalization to MNI space. A generalized linear model analysis, including the four experimental runs, was performed to estimate the BOLD responses (beta estimates) associated with the different experimental conditions. The model included seven regressors, one for each experimental condition, convoluted with the hemodynamic response, as well as the six rigid-body motion parameters and the frame-wise displacement as nuisance regressors^56^ (total of seven nuisance regressors). The software mricroGL (https://www.nitrc.org/projects/mricrogl/) was used for visualization of the results. At the single-participant level, the following contrasts were computed in each cohort:

1. contrasts 1 and 2 to highlight the multisensory activations common to infectious/fearful and neutral conditions:
  contrast 1 (VTFinfectious/VTFfearful + VTNinfectious/VTNfearful)
  contrast 2 (VTFneutral + VTNneutral)
2. contrast 3 to highlight the activations specific to infectious avatars in the far space compared to neutral avatars:
  contrast 3 (VTFinfectious/VTFfearful > VTFneutral)
3. contrast 4 to highlight the activations specific to infectious avatars in the near space compared to neutral avatars:
  contrast 4 (VTNinfectious/VTNfearful > VTNneutral)
4. contrast 5 to highlight the difference between contrasts 3 and 4:
  contrast 5 (VTFinfectious/VTFfearful – VTFneutral > VTNinfectious/VTNfearful – VTNneutral)

These single-participant contrasts were then used to compute the following group-level statistical tests: (1) one-sample *t*-test with contrast 1 in the infectious cohort (VTinfectious), (2) one-sample *t*-test with contrast 2 in the infectious cohort (VTneutral), (3) one-sample *t*-test with contrast 1 in the fearful cohort (VTfearful), (4) one-sample *t*-test with contrast 2 in the fearful cohort (VTneutral), (5) one-sample *t*-test with contrast 3 in the infectious cohort (dVTFi: VTFi > VTFn), (6) one-sample *t*-test with contrast 4 in the infectious cohort (dVTNi: VTNi > VTNn), (7) one-sample *t*-test with contrast 5 in the infectious cohort (dVTFi > dVTNi), (8) two-sample *t*-test (infectious versus fearful) with contrast 3 (dVTFi > dVTFf), (9) two-sample *t*-test (infectious versus fearful) with contrast 4 (dVTNi > dVTNf) and (10) two-sample *t*-test (infectious versus fearful) with contrast 5 (dVTFi > dVTNi) > (dVTFf > dVTNf).

Finally, to control that our results cannot be explained by a simple effect of disgust during exposure to infectious avatars (for example, an increase in brain activity when disgust is experienced), we computed an additional one-sample *t*-test at the group level with contrast 3 by including as a covariate an assessment of sensitivity to disgusting stimuli using the germ aversion questionnaire^57^.

Whole-brain results were corrected for multiple comparisons using family-wise error cluster-level correction at *P* < 0.05 (with a primary threshold of *P* < 0.005; Supplementary Fig. 23).

### DCM

DCM (SPM12) analyses were performed to investigate the connectivity between the hypothalamus (central node of the HPA axis) and the cortical regions that were associated with infectious avatars presented in far space. The hypothalamus was delineated using the parcellation pipeline of the Connectome Mapper 3 software (v3.1.0; https://connectome-mapper-3.readthedocs.io/). The cortical regions activated by infectious avatars presented in the far space were selected from group comparisons 5 and 7. To extract the time series from each selected region, a generalized linear model with the four functional runs concatenated was computed. We directly modeled BOLD fluctuations associated with the difference between infectious and neutral conditions by defining a ‘task’ condition (for example, VTF for both type of faces) and an ‘avatar’ parametric modulation (1 for infectious trials and −1 for neutral trials). Next, we computed two DCM analyses (neural model options: bilinear neural model, one state per region, no stochastic effects, with input centering), one for each hemisphere (cortical regions selected from contrasts 5 and 7 in the right hemisphere + right hypothalamus and cortical regions selected from contrasts 5 and 7 in the left hemisphere + left hypothalamus). The ‘task’ and ‘avatar’ conditions for VTF were included in the two DCM analyses. The driving input C was enabled for all regions of the ‘task’ condition, but not for the ‘avatar’ condition. The baseline connectivity A was initialized to a fully connected network. The modulatory connectivity B was enabled for the ‘avatar’ condition and was restricted to self-connections and connections between the hypothalamus and all other regions. The parametric empirical Bayes framework was used to identify (at the group level) the connections that were differently modulated across VTFinfectious and VTFneutral conditions. Connections were considered significantly modulated (VTFinfectious > VTFneutral or VTFinfectious < VTFneutral) when a posterior probability of 0.95 was reached. As control analyses, the same DCM analyses were computed with the corresponding conditions presented in the near space (VTNinfectious and VTNneutral), as well as in the fearful cohort.

### Serum analysis

Serum samples were thawed and analyzed using different methods. For steroid quantification, serum samples (100 µl) were mixed with 550 µl of 5% H_3_PO_4_ and 75 µl of internal standard solution and extracted by solid-phase extraction on an OASIS MCX µElution 96-well plate (30 µm, 2 mg). Wells were washed and conditioned with 200 µl of methanol and 200 µl of water, respectively. Loaded samples were washed with 200 µl of 5% NH_4_OH and twice with 200 µl of water:methanol (4:1 (vol/vol)), and steroids were eluted with 2 × 100 µl of isopropanol. The solvent was then evaporated to dryness under N_2_ gas (TurboVap, Biotage), and final extracts were reconstituted with 100 µl of methanol. Extracted samples were analyzed by reversed-phase liquid chromatography (LC) coupled to tandem MS (MS/MS) in both positive and negative ionization mode using a TSQ Altis triple-quadrupole system interfaced with a Vanquish UHPLC system (Thermo Fisher Scientific). Chromatographic separation was performed in an Accucore aQ C18 column (2.6 μm, 100 mm × 2.1 mm inner diameter; Thermo Fisher Scientific). The mobile phase was composed of A (0.25 mM ammonium fluoride in water) and B (100% methanol) at a flow rate of 250 μl min^−1^, column temperature of 40 °C and sample injection volume of 2 µl. Gradient elution was performed with 70% A as the starting condition for 1 min and linearly decreased to 55% at 1.5 min, 15% at 5 min and 0% at 6 min to 7 min. The column was then washed with solvent B for 1 min and equilibrated to initial conditions. ESI source conditions were set as follows: vaporizer temperature of 350 °C, sheath gas of 50 arbitrary units (AU), auxiliary gas of 13 AU and sweep gas of 1 AU. The ion transfer tube temperature was set at 275 °C, the positive ion spray voltage was set at +3,500 V, and the negative ion spray voltage was set at −2,800 V. Scheduled multiple reaction monitoring with polarity switching was used as the acquisition mode with a minimum dwell time between 8 and 22 ms. Optimized collision energies for each metabolite were applied. Raw LC–MS/MS data were processed using TraceFinder 5.0 software (Thermo Fisher Scientific). For absolute quantification, calibration curves and stable isotope-labeled internal standards were used to determine the response factor. Linearity of the standard curves was evaluated for each metabolite using a 12-point range; in addition, peak area integration was manually curated and corrected when necessary.

For eicosanoid quantification, serum samples (150 µl) were mixed with 150 µl of extraction buffer (citric acid/Na_2_HPO_4_, pH 5.6) and 10 µl of internal standard solution and extracted by solid-phase extraction using an OASIS HLB LP 96-well plate (60 µm, 60 mg). Wells were conditioned and equilibrated with 1 ml of methanol and 1 ml of water, respectively. Loaded samples were washed with water:methanol (90:10 (vol/vol)), and eicosanoids were eluted with 750 µl of methanol. The solvent was then evaporated to dryness under N_2_ gas (TurboVap, Biotage), and final extracts were reconstituted with 75 µl of methanol:water (6:1 (vol/vol)). Extracted samples were analyzed by reversed-phase LC–MS/MS^40^ in negative ionization mode using a 6495 triple-quadrupole system interfaced with a 1290 UHPLC system (Agilent Technologies). Chromatographic separation was performed in an Acquity BEH C18 column (1.7 μm, 150 mm × 2.1 mm inner diameter; Waters). The mobile phase was composed of A (water with 0.1% acetic acid) and B (acetonitrile:isopropanol 90:10 (vol/vol)) at a flow rate of 500 μl min^−1^, column temperature of 60 °C and sample injection volume of 2 µl. Gradient elution was performed with 80% A as the starting condition and was linearly decreased to 65% at 2.5 min, 60% at 4.5 min, 58% at 6 min, 50% at 8 min, 35% at 14 min, 27.5% at 1 min and 0% at 16.6 min. The column was then washed with solvent B for 0.9 min and equilibrated to initial conditions. ESI source conditions were set as follows: dry gas temperature of 290 °C, nebulizer at 25 psi and flow of 12 l min^−1^, sheath gas temperature of 400 °C and flow of 12 l min^−1^, nozzle voltage of 2,000 V and capillary voltage of 3,000 V. Dynamic multiple reaction monitoring was used as the acquisition mode with a total cycle time of 250 ms. Optimized collision energies for each metabolite were applied^58^. Raw LC–MS/MS data were processed using Agilent Quantitative analysis software (version B.07.00, MassHunter Agilent Technologies). For absolute quantification, calibration curves and stable isotope-labeled internal standards were used to determine the response factor. Linearity of the standard curves was evaluated for each metabolite using a 12-point range; in addition, peak area integration was manually curated and corrected when necessary.

For other soluble mediators, the ‘human neuroinflammation panel 1 (740796)’ LEGENDplex kit (Biolegend) was used, according to manufacturer’s instructions. This platform allowed for the quantification of the concentration of 13 different soluble factors at the same time, namely VILIP-1, CCL2 (MCP-1), sTREM-2, BDNF, TGFβ1, VEGF, IL-6, sTREM-1, β-NGF, IL-18, TNF, sRAGE and CX3CL1 (Fractalkine).

Steroids, eicosanoids and neuroinflammatory factors were analyzed and plotted using GraphPad Prism version 10.4.1 (Supplementary Figs. 17–19).

### Neural network prediction of ILC activation from serum multiOMICS after exposure to infectious avatars

Univariate and multivariate linear regressions were first attempted to predict VR-induced changes in ILC activation from changes in eicosanoid, neuroinflammatory factor and hormone concentrations. These analyses resulted in nonsignificant effects (univariate: *R*^2^ = 0.001, 0.015 and 0.058 and *P* = 0.86, 0.53 and 0.20, for eicosanoids, neuroinflammatory factors and hormones, respectively; multivariate: *R*^2^ = 0.13 and *P* = 0.3), suggesting that the neural signals triggering an immune response are based on a more complex, nonlinear combination of inputs. For this reason, we developed a one-hidden-layer neural network with changes in eicosanoid, neuroinflammatory factor and hormones concentrations as input and changes in ILC activation as output (Supplementary Method 6).

### Statistics

All data were checked for normality distribution (Shapiro–Wilk test). Because the majority of data were normally distributed, to ensure uniform statistical testing, we performed two-sided *t*-tests in all comparisons. Given the absence of previous studies assessing immunological responses to virtual threats, we first calculated the required sample size for the behavioral multisensory experiment. Based on previous experiments^17,19^, an averaged effect size *f* = 0.403 was calculated. Thus, we originally estimated, for immunomonitoring, a sample size of 15 participants per group, with a desired power of 0.95 (1 − *β*) on within-group comparisons via G*Power 3.1 software. The sample size for the other experiments was then established accordingly. For the replication experiment, based on the effect size of the data presented in the paper of the original cohorts (neural versus infection cohorts: Cohen’s *d* = 1.114 for ILC frequency and *d* = 1.116 for ILC activation), the sample size with a *P* value of <0.05 and a power of 0.80 was determined to be 14 participants per cohort. To minimize the risk of dropouts and data loss, we enrolled and analyzed 16 participants per group.

### Reporting summary

Further information on research design is available in the Nature Portfolio Reporting Summary linked to this article.

## Online content

Any methods, additional references, Nature Portfolio reporting summaries, source data, extended data, supplementary information, acknowledgements, peer review information; details of author contributions and competing interests; and statements of data and code availability are available at 10.1038/s41593-025-02008-y.

## Supplementary information

Supplementary Information Supplementary Tables 1–10, Figs. 1–28, Methods 1–6 and References 1–17.

Reporting Summary

## Data Availability

All data are available in the main text or the Supplementary Information. All data files can be found at https://osf.io/rg9fa/files/osfstorage.

## Code availability

Custom code to replicate the results shown in Fig. 5 can be found at https://osf.io/rg9fa/files/osfstorage.
