# Addendum: Neural anticipation of virtual infection triggers an immune response

**DOI:** 10.1038/s41593-026-02284-2

**Published:** 2026-04-24

**Authors:** Sara Trabanelli, Michel Akselrod, Julia Fellrath, Giulia Vanoni, Tommaso Bertoni, Silvia Serino, Georgia Papadopoulou, Maren Born, Matteo Girondini, Giuseppe Ercolano, Giulia Ellena, Anthony Cornu, Giulio Mastria, Hector Gallart-Ayala, Julijana Ivanisevic, Petr Grivaz, Maria Paola Paladino, Camilla Jandus, Andrea Serino

**Affiliations:** 1 Departement of Oncology, UNIL-CHUV, University of Lausanne, Epalinges, Switzerland; 2 Targeting of Cytokine Secreting Lymphocytes (TCSL) Laboratory, Department of Pathology and Immunology, Faculty of Medicine, University of Geneva, Geneva, Switzerland; 3 Ludwig Institute for Cancer Research, Lausanne Branch, Lausanne, Switzerland; 4 Geneva Centre for Inflammation Research (GCIR), Geneva, Switzerland; 5 Translational Research Centre in Onco-Hematology (CRTOH), Geneva, Switzerland; 6 MySpace Lab and NeuroRehab Research Center, Service of University Neurorehabilitation (SUN), Lausanne University Hospital, Institution of Lavigny and University of Lausanne, Switzerland, Switzerland; 7 Department of Psychology, Alma Mater Studiorum, University of Bologna, Bologna, Italy; 8 Metabolomics and Lipidomics Platform, Faculty of Biology and Medicine, University of Lausanne, Lausanne, Switzerland; 9 Department of Psychology and Cognitive Science, University of Trento, Trento, Italy; 10 Present Address: School of Health Sciences, HES-SO Valais-Wallis, Sion, Switzerland; 11 Present Address: Département Hospitalier, Institution de Lavigny, Lavigny, Switzerland; 12 Present Address: Translational Neural Engineering Lab, Neuro-X Institute, École Polytechnique Fédérale de Lausanne (EPFL), Lausanne, Switzerland; 13 Present Address: Department of Psychology, University of Milano-Bicocca, Milan, Italy; 14 Present Address: Department of Pharmacy, University of Naples Federico II, Naples, Italy; 15 Present Address: Center for Mind/Brain Sciences–CIMeC, University of Trento, Rovereto, Italy; 16 Present Address: Human Islet Isolation Facility, Nuffield Department of Surgical Sciences, University of Oxford, Oxford, UK; 17 Present Address: Nuffield Department of Clinical Neurosciences, John Radcliffe Hospital, Oxford, UK

**Keywords:** Perception, Innate immune cells

Addendum to: *Nature Neuroscience* 10.1038/s41593-025-02008-y, published online 28 July 2025.

The authors wish to acknowledge previous research that has provided evidence for links between perceptual processing of disease-related cues and biological immune responses. A seminal study reported that viewing images depicting symptoms of infectious disease enhanced the IL-6 response in blood samples exposed to a subsequent immune challenge^1^. Further studies reported changes in salivary immune markers (including cytokines, TNF-α and secretory immunoglobulin A, sIgA) upon exposure to disease-related cues such as images, videos or virtual reality interactions with individuals displaying symptoms of illness (for example, sneezing or coughing)^2–8^. However, in some of these studies, comparable changes in immune markers were observed following exposure to both potentially contagious and control cues^3,6–8^. In addition, salivary immune monitoring presents methodological challenges, so that more than one quarter of the collected saliva samples were excluded from analysis because of insufficient sample quality or reliability. As a result, findings based solely on salivary immune markers should be interpreted with caution and should be considered as preliminary indicators of immune modulation. More robust immuno-monitoring analyses should rather focus on soluble markers, immune cell frequency and activation measurements, and bulk RNA profiling in the blood samples of participants exposed to infection-related cues.

Another line of research has investigated the neural processing of disease cues using functional magnetic resonance imaging^8–10^. These studies examined responses to visual and even olfactory signs of illness and reported activation in regions including middle frontal and orbitofrontal cortex (associated with salience evaluation and decision making), higher-order sensory regions, such as inferior temporal and occipital cortices, multisensory areas, processing exteroceptive, e.g., intraparietal sulcus, or interoceptive, i.e., insula - body-related stimuli. Together, these findings demonstrate neural sensitivity to sickness cues, but not whether such neural responses translate into anticipatory activation of the immune system, nor through which neuro-immune pathways such interactions might occur.

The findings from Trabanelli et al. extend the framework of anticipatory neuro-immune activation in humans, demonstrating that predictive neural processes may engage immune pathways in preparation for potential infection.
