# Supplementary material for: Neural anticipation of virtual infection triggers an immune response
Source: Nat Neurosci. 2025 Jul 28;28(9):1968–77. doi: 10.1038/s41593-025-02008-y (PMC12411218; doi:10.1038/s41593-025-02008-y)
Supplement: Supplementary file 1 — Supplementary Tables 1–10, Figs. 1–28, Methods 1–6 and References 1–17. [file 41593_2025_2008_MOESM1_ESM.pdf]

nature neuroscience

Supplementary information https://doi.org/10.1038/s41593-025-02008-y

Neural anticipation of virtual infection triggers an immune response

In the format provided by the authors and unedited

Table S1

	Neutral	Infectious	*t*(19)	*p*
Pleasant	5.36 (.28)	1.71(.15)	12.57	<.001
Unpleasant	1.68 (.20)	5.41(.26)	10.74	<.001
Healthy	6.02 (.22)	1.43(.08)	19.61	<.001
Sick	1.38 (.12)	6.14(.13)	24.24	<.001
Contagiousness	1.40 (.14)	5.14(.22)	12.27	<.001
Realistic	4.84 (.30)	3.37(.24)	4.75	<.001
Seating distance	2.02 (.24)	4.39(.36)	5.96	<.001

Table S1. Attitudes towards virtual infectious threats. Results from paired sample t-tests between neutral and infectious faces on the following attributes: realistic, pleasant, unpleasant, healthy, sick, contagious, interpersonal distance. Mean (SD) are presented.

Results from the three different assessments confirmed that infectious faces were perceived both implicitly and explicitly as sick and contagious, and evoked automatic avoidance behaviours. In the implicit association task, the IAT D score was large and positive ($t(19) = 9.51$, $P < 0.001$), showing that infectious faces were preferentially associated with avoidant reactions and neutral faces with approach reactions (than the other way around).

For each dimension, explicit ratings were compared between healthy and infectious avatars. Paired sampled t-tests showed significant differences between avatars for all dimensions. Neutral avatars scored higher in terms of attractiveness and realism, whereas infectious avatars were perceived as sicker and more contagious.

Finally, larger seating distance[1] was reported toward infectious than neutral faces.

Interestingly, larger distance toward the infectious avatar’s was related to their perceived contagiousness ($r = 0.59$, $P < 0.01$) and overall unpleasantness ($r = 0.58$, $P < 0.01$ and $r = -0.46$, $P < 0.05$).

Table S2

Block	Task description	Number of trials	Function
1	Healthy vs. Sick faces categorization	36	practice
2	Approach vs. avoidance categorization	36	practice
3	Combined task (familiarization phase)	36	practice
4	Combined task	96	test
5	Healthy vs. Sick faces categorization (reversed task instruction)	36	practice
6	Combined task (familiarization phase)	36	practice
7	Combined task	96	test

Table S2. Implicit Attitude Test: block task description.

Table S3

**Two-sample t-test**	**T-value**	**P-value**
Disgust sensitivity<br>*Behavioural responses to virtual infectious threats entering the PPS experiment*		
infectious vs fearful	0.32	0.75
infectious vs neutral	0.25	0.80
fearful vs neutral	0.09	0.93
Anxiety sensitivity<br>*Behavioural responses to virtual infectious threats entering the PPS experiment*		
infectious vs fearful	1.47	0.15
infectious vs neutral	0.96	0.35
fearful vs neutral	0.68	0.50
Disgust sensitivity<br>*Immune responses to virtual and real infectious threats*		
infectious vs fearful	-0.18	0.86
infectious vs neutral	-0.59	0.56
fearful vs neutral	0.42	0.68
Anxiety sensitivity<br>*Immune responses to virtual and real infectious threats*		
infectious vs fearful	0.91	0.37
infectious vs neutral	0.77	0.44
fearful vs neutral	0.05	0.96
Disgust sensitivity<br>*Neuroimaging responses to virtual infection threats*		
infectious vs fearful	-0.74	0.46
Anxiety sensitivity<br>*Neuroimaging responses to virtual infection threats*		
infectious vs fearful	0.09	0.93

Table S3. Sensitivity to disgust and anxiety in the different cohorts.

Table S4

	Region	XYZ (MNI)	Brodmann area	*t*-value
**1) 87-168ms**	Parietal Lobe, Precuneus	20 -50 35	31	6.80*
		15 -50 45	7	6.72*
		15 -50 40	7	6.67*
		15 -50 35	31	6.43±
**2) 185-246ms**	Frontal lobe, Precentral gyrus	35 -25 70	6	6.13±

* *p*-value < 0.05

± *p*-value < 0.10

Table S4. **Electrophysiological correlates of PPS.** Statistical non-parametric comparisons between current source density values of visuo-tactile near vs. visuo-tactile far stimulations. We investigated electrophysiological correlates of PPS using GFP[2] extracted from the 128 electrodes and performed analyses within the time window associated to multisensory response (87 to 400 ms). As illustrated in the main text (see Fig. 1E), the contrast between visuo-tactile near and visuo-tactile far GFP measures revealed two significant time windows: from 87 to 168ms (t1, $P < 0.05$, FDR corrected) and from 185 to 246ms (t2, $P < 0.05$, FDR corrected), in accordance with previous electrophysiological literature.[3] Since the size of the faces for the near and the far distances occupied the same visual angle, these different space dependent responses could not be attributed to different low-level visual features in the stimuli processing. To localize these PPS-related differences, sLORETA was applied for each time point within the two significant time-windows (t1 and t2). The strongest effect for the first time-window was found at 102ms over the right precuneus/inferior parietal regions. For the second time-window, we found a statistical trend at 220ms in the right precentral gyrus ($P < 0.10$).

Table S5

	Region	XYZ (MNI)	Brodmann area	*t*-value
**129-150ms**	Parietal Lobe, Postcentral gyrus	20 -40 60	3	7.08*
	Parietal Lobe, Precuneus	10 -50 55	7	6.98±
	Parietal Lobe, Postcentral gyrus	20 -40 65	3	6.80*

* *p*-value < 0.05

± *p*-value < 0.10

Table S5. Different responses at far distance between neutral and infectious avatars. Statistical non-parametric comparisons between source density values of infectious vs. neutral avatars presented in the far space in the infection cohort.

To understand the origin of the previous distance effect in the infection cohort, we performed post-hoc comparisons in the time window between 129 and 150ms and compared GFP responses associated to an infectious or a neutral avatar presented at near or far distance. We found no significant difference between VT neutral, and VT infectious avatars presented in the near space. In contrast, this difference was significant between neutral and infectious avatars presented in the far space (between 129-150ms; $P < 0.05$, FDR corrected). Source localization of this GFP difference revealed a peak of current density at 145ms, localized over the right postcentral areas ($P < 0.05$) and a statistical trend over the right parietal/precuneus region ($P < 0.10$).

Table S6

Cohort	Coefficient	SE	p-value
All	1.15	0.09	<0.0001
Neutral	0.93	0.13	<0.0001
Infection	1.25	0.23	0.0001
Fearful	0.88	0.40	0.046
Vaccine	1.03	0.15	<0.0001

Table S6. Estimated coefficients for the regression between the synthetic ILC frequency and activation indexes for the overall sample and each cohort.

Table S7

contrast VTFi > VTFn	Anatomy	Voxels	T-value	X Y Z
Visual ventral left (VISv)	Lingual L	51	5.1	-24 -56 -12
	Occipital Inf L	105	5.4	-34 -76 -12
	Fusiform L	356	8.6	-34 -54 -16
	Temporal Mid L	11	5.0	-42 -64 -6
	Temporal Inf L	66	6.3	-44 -46 -10
Visual ventral right (VISv)	Lingual R	20	4.9	26 -58 -12
	Occipital Inf R	41	4.4	34 -70 -12
	Fusiform R	324	7.8	30 -56 -16
	Temporal Mid R	29	8.2	42 -62 -2
	Temporal Inf R	77	5.5	42 -64 -8
Visual dorsal left (VISd)	Occipital Mid L	290	5.7	-32 -88 18
Visual dorsal right (VISd)	Occipital Mid R	164	5.3	30 -86 6
Occipitoparietal Junction left (OPJ)	Occipital Mid L	67	4.7	-28 -66 30
	Parietal Sup L	12	3.8	-22 -62 40
Occipitoparietal Junction right (OPJ)	Occipital Sup R	93	4.6	28 -74 36
	Occipital Mid R	61	4.6	30 -70 34
Intraparietal sulcus right (IPS)	Parietal Sup R	145	4.7	18 -68 56
	Parietal Inf R	70	5.9	26 -56 50
	Angular R	17	3.7	30 -62 38
Primary Somatosensory right (S1)	Postcentral R	80	6.4	40 -30 48
	Parietal Inf R	15	3.6	40 -36 48
	SupraMarginal R	15	3.4	38 -32 42
Premotor cortex left (PMC)	Precentral L	71	4.1	-42 4 20
	Frontal Inf Oper L	18	3.7	-56 10 28
Premotor cortex right (PMC)	Precentral R	73	4.8	56 12 30
	Frontal Inf Oper R	51	4.9	56 12 28
Midde frontal gyrus left (MFG)	Frontal Mid L	77	4.8	-38 22 38
	Frontal Mid L	76	3.9	-28 30 28
	Frontal Inf Oper L	23	3.9	-38 18 32
	Frontal Inf Tri L	46	3.8	-50 26 28
Midde frontal gyrus right (MFG)	Frontal Mid R	71	4.2	28 34 40
	Frontal Inf Tri R	44	3.7	50 28 20
	Frontal Sup R	25	3.3	22 44 22
	Frontal Mid R	33	3.5	20 46 26
Anterior cingulate cortex (ACC)	Cingulum Ant L	57	5.4	-6 32 24
	Cingulum Ant R	21	3.9	0 22 26
	Cingulum Ant R	14	3.8	10 24 28
	Cingulum Mid R	39	4.1	10 24 30
Anterior Insula right (aINS)	Insula Ant R	49	4.0	34 26 -4

contrast VTNi > VTNn	Anatomy	Voxels	T-value	X Y Z
Visual ventral left (VISv)	Lingual L	75	4.6	-28 -84 -16
	Occipital Inf L	182	6.6	-34 -76 -12
	Fusiform L	347	5.8	-30 -52 -16

	Occipital Inf L	37	4.3	-44 -70 -14
	Temporal Mid L	23	3.9	-48 -56 -6
	Temporal Inf L	52	4.9	-48 -56 -8
Visual ventral right (VISv)	Lingual R	61	4.9	26 -56 -12
	Occipital Inf R	41	5.9	28 -86 -16
	Fusiform R	272	5.6	28 -44 -18
Visual dorsal left (VISd)	Occipital Mid L	317	4.8	-34 -84 22
Visual dorsal right (VISd)	Occipital Mid R	71	4.4	34 -78 22
Occipitoparietal Junction left (OPJ)	Occipital Mid L	63	4.1	-28 -68 38
	Parietal Sup L	63	3.7	-26 -72 48
	Parietal Inf L	51	4.3	-30 -68 38
	Angular L	15	3.5	-38 -72 36
Occipitoparietal Junction right (OPJ)	Occipital Sup R	61	4.4	26 -72 44
	Occipital Mid R	23	3.7	30 -70 34
	Parietal Sup R	12	3.2	26 -72 52
	Occipital Sup R	13	4.1	26 -90 10
	Occipital Mid R	107	5.6	28 -90 10
Intraparietal sulcus left (IPS)	Parietal Sup L	21	3.7	-24 -52 48
	Parietal Inf L	53	3.8	-32 -58 50
Intraparietal sulcus right (IPS)	Parietal Sup R	44	4.4	30 -46 48
	Parietal Inf R	56	6.8	30 -48 50

**contrast (VTFi > VTFn) > (VTNi > VTNn)**	**Anatomy**	**Voxels**	**T-value**	**X Y Z**
Visual ventral right (VISv)	Temporal Mid R	46	5.2	42 -66 -2
	Temporal Inf R	22	4.6	46 -66 -8
Anterior Insula right (aINS)	Insula R	22	4.3	30 26 2
	Frontal Inf Tri R	43	6.0	48 22 2
Anterior Insula left (aINS)	Insula L	95	6.1	-26 24 10
	Frontal Inf Tri L	30	5.0	-44 20 -2
Middle frontal gyrus left (MFG)	Frontal Mid L	114	5.9	-30 48 22
	Frontal Sup L	29	5.7	-26 48 22
Medial prefrontal cortex left (mPFC)	Frontal Sup Medial L	46	5.4	-6 50 18
Middle cingulate cortex (ACC)	Cingulum Mid L	62	4.5	-8 14 34
	Supp Motor Area L	12	4.2	-6 12 44

Table S7. fMRI activations in the infectious cohort for contrasts 3 (activations specific to infectious avatars presented in the far space), 4 (activations specific to infectious avatars presented in the near space) and 5 (Activations specific to infectious avatars presented in the far vs near spaces). We contrasted the BOLD responses evoked by visuo-tactile stimulation for infectious vs. neutral avatars in the far space (Fig. 4C in the main text, contrast VTFi > VTFn). Significant activations (infectious > neutral in the far space) were found bilaterally in visual areas of the ventral and dorsal streams (occipital, fusiform, lingual and temporal gyri), bilateral occipito-parietal junction (OPJ), bilateral premotor cortex (PMC), bilateral middle frontal gyrus (MFG), the right intraparietal sulcus (IPS), the right primary somatosensory cortex (S1), and the right anterior Insula (aINS), and the left anterior cingulate

cortex (ACC). As for the far space, we also contrasted the BOLD responses evoked by visuo-tactile stimulation for infectious vs. neutral avatars in the near space (Fig. 4C in the main text, contrast VTNi > VTNn). Significant activations (infectious > neutral in the near space) were found bilaterally in visual areas of the ventral and dorsal streams (occipital, fusiform, lingual and temporal gyri), bilateral occipito-parietal junction (OPJ) and bilateral intraparietal sulcus (IPS). These activations (infectious > neutral) were overlapping for the far and near spaces (contrasts 3 and 4), thus we did not find any activations (infectious > neutral) that were specific for the near space. We then further contrasted the difference in BOLD response evoked by tactile stimuli coupled with infection vs. neutral avatars in the far as compared to the near space (Fig. 4C in the main text, contrast (VTFi > VTFn) > (VTNi > VTNn)). We found activations in right visual areas, left medial prefrontal cortex (mPFC), left middle cingulate cortex (MCC), bilateral anterior Insula (aINS) and left frontal middle gyrus (MFG).

Table S8

contrast (VTFi > VTFn) vs (VTFf > VTFn)	Anatomy	Voxels	T-value	X Y Z
Visual ventral left (VISv)	Lingual L	29	3.7	-24 -66 -10
	Occipital Inf L	51	4.4	-44 -58 -16
	Fusiform L	247	4.8	-36 -62 -14
	Temporal Mid L	13	4.3	-48 -60 -6
	Temporal Inf L	84	4.7	-48 -62 -8
	Cerebellum 6 L	26	3.6	-26 -50 -20
Visual ventral right (VISv)	Temporal Mid R	21	3.7	42 -62 -4
	Temporal Inf R	68	4.4	54 -54 -12
Visual dorsal left (VISd)	Occipital Mid L	165	4.9	-36 -80 12
Visual dorsal right (VISd)	Occipital Mid R	69	4.4	36 -80 20
Occipitoparietal junction left (OPJ)	Occipital Mid L	64	3.8	-26 -74 32
Occipitoparietal junction right (OPJ)	Occipital Sup R	66	4.5	30 -72 44
	Angular R	18	3.6	30 -68 46
Inferior parietal lobule left (IPL)	Parietal Inf L	45	3.7	-58 -24 38
	SupraMarginal L	26	4.0	-58 -26 38
Inferior parietal lobule right (IPL)	Postcentral R	77	5.1	38 -28 40
	Parietal Inf R	25	4.0	40 -36 48
	SupraMarginal R	33	4.3	40 -30 38
Intraparietal sulcus right (IPS)	Parietal Sup R	223	4.8	30 -72 46
	Parietal Inf R	61	4.2	26 -56 52
Anterior Insual right (aINS)	Insula R	51	4.5	36 12 0
	Putamen R	20	5.0	30 14 -4
Premotor cortex left (PMC)	Precentral L	84	4.0	-48 -4 32

contrast (VTNi > VTNn) vs (VTNf > VTNn)	Anatomy	Voxels	T-value	X Y Z
Visual ventral left (VISv)	Fusiform L	112	4.7	-28 -44 -22
Visual ventral right (VISv)	Calcarine R	62	4.0	12 -94 4
	Cuneus R	59	4.4	14 -94 4
	Occipital Mid R	30	3.5	28 -90 10
Visual dorsal left (VISd)	Calcarine L	26	4.5	-10 -92 -10
	Occipital Sup L	48	3.8	-12 -96 2
	Occipital Mid L	17	3.6	-12 -96 0
Visual dorsal right (VISd)	Occipital Mid R	88	4.3	36 -78 20
	Occipital Sup R	52	4.1	28 -66 34

contrast (VTFi > VTFn) > (VTNi > VTNn) vs (VTFf > VTFn) > (VTNf > VTNn)	Anatomy	Voxels	T-value	X Y Z
Midde frontal gyrus left (MFG)	Frontal Mid L	104	5.6	-30 46 26
	Frontal Sup L	42	4.4	-20 40 30
Midde frontal gyrus right (MFG)	Frontal Mid R	48	4.3	30 34 26
Medial prefrontal cortex left (mPFC)	Frontal Sup Medial L	18	3.7	0 26 40
Middle cingulate cortex (MCC)	Cingulum Mid L	24	3.5	-8 16 34

	Cingulum Mid R	79	4.5	2 26 38
Anterior Insula right (aINS)	Insula R	53	4.7	40 14 -4
	Insula R	36	3.9	30 26 2
	Frontal Inf Tri R	15	3.6	32 32 2
	Putamen R	11	3.7	32 8 0
Premotor cortex left (PMC)	Frontal Inf Oper L	78	5.0	-54 16 12
Middle temporal gyrus right (MTG)	Temporal Mid R	39	3.7	54 -44 8
	Temporal Sup R	42	4.3	50 -40 10

Table S8. fMRI activations in the infectious vs fearful cohorts for contrasts 3 (Activations specific to infectious avatars presented in the far space in comparison to fearful avatars), 4 (Activations specific to infectious avatars presented in the near space in comparison to fearful avatars) and 5 (Activations specific to infectious avatars presented in the far vs near spaces in comparison to fearful avatars). To control that our results are specific for infectious avatars presented in the far space, we directly compared contrast 3 between the infectious and fearful cohorts (two-sample t-test with contrasts VTFi > VTFn vs VTFf > VTFn). We found activations in bilateral visual areas of the ventral and dorsal streams (occipital, fusiform, lingual, and temporal gyri), bilateral occipito-parietal junction (OPJ), right intraparietal sulcus (IPS), right primary somatosensory cortex (S1), right anterior Insula (aINS), left inferior parietal lobule and left premotor cortex (PMC).

We then computed the same comparison for infectious avatars presented in the near space and directly compared contrast 4 between the infectious and fearful cohorts (two-sample t-test with contrasts VTNi > VTNn vs VTNf > VTNn). We found activations in bilateral visual areas of the ventral and dorsal streams (occipital, fusiform, lingual, and temporal gyri).

Finally, we computed the same comparison for infectious avatars presented in the far vs near space and directly compared contrast 5 between the infectious and fearful cohorts (two-sample t-test with contrasts (VTFi > VTFn) > (VTNi > VTNn) vs (VTFf > VTFn) > (VTNf > VTNn)). We found activations in bilateral middle frontal gyrus (MFG), right middle temporal gyrus (MTG), right anterior Insula (aINS), left premotor cortex (PMC) and left anterior cingulate cortex (ACC).

Table S9

Compound	RT (min)	Polarity	Precursor (m/z)	Product (m/z)	Collision Energy (V)	Min Dwell Time (ms)
Estrone sulfate	2.82	Negative	349.1	145.1	54	12
			349.1	269.2	32	12
DL-SODIUM ESTRONE 3-SULFATE (13C6)	2.82	Negative	355.5	275.2	31	12
DHEAS	3.01	Negative	367.2	80.0	55	12
			367.2	97.0	33	12
DHEAS (D5)			372.2	98.0	36	12
Pregnenolone sulfate	3.43	Negative	395.5	97.0	35	11
			395.5	97.0	35	11
Pregnenolone sulfate (D4)			399.5	97.0	36	11
Cortisone	3.53	Positive	361.2	163.1	24	11
			361.2	299.2	22	11
			361.2	343.0	16	11
CORTISONE-(2,3,4-13C3)	3.53	Positive	364.2	166.2	24	11
			364.2	346.2	16	11
Cortisol	3.68	Positive	363.2	121.0	24	11
			363.2	309.1	17	11
			363.2	327.1	16	11
CORTISOL-D4			367.2	121.1	25	11
			367.2	331.4	16	11
Corticosterone	4.15	Positive	347.1	120.9	25	9
			347.1	311.1	16	9
			347.1	121.1	25	9
			347.1	329.3	15	9
11-deoxycortisol	4.23	Positive	347.2	96.9	25	8
			347.2	109.0	27	8
11-DEOXYCORTISOL (D5)			352.4	100.1	25	8
Androstenedione	4.65	Positive	287.1	96.9	22	8
			287.1	109.0	24	8
Androstene-3,17-dione (2,3,4-13C3)			290.1	100.1	22	8
Estrone	4.72	Negative	269.2	143.1	53	8
			269.2	145.1	38	8
			269.2	183.0	37	8
ESTRONE (2,3,4-13C3)			272.4	148.1	38	8
11-deoxycorticosterone	4.79	Positive	331.2	97.0	20	8
			331.2	109.0	24	8

11-Deoxycorticosterone (2,3,4-13C3)			334.2	100.2	23	8
17-OH-P	4.94	Positive	331.2	96.9	24	8
			331.2	109.0	26	8
17ALPHA-HYDROXYPROGESTERONE (2,3,4-13C3)	4.94	Positive	334.2	112.1	27	8
Testosterone	4.95	Positive	289.1	96.9	22	8
			289.1	109.0	25	8
Testosterone (2,3,4-13C3)			292.5	112.1	25	8
EpiTestosterone	5.12	Positive	289.2	97.1	23	8
			289.2	109.1	25	8
17-HydroxyPregnenolone	5.16	Negative	331.2	287.2	19	8
			331.2	313.3	20	8
17ALPHA-HYDROXYPREGNENOLONE (D3)			333.2	287.3	20	8
Progesterone	5.76	Positive	315.2	96.9	22	10
			315.2	109.0	25	10
Progesterone (D9)			324.2	113.1	27	10
Allopregnanolone	6.41	Positive	319.3	283.3	13	22
			319.3	301.3	9	22
ALLOPREGNANOLONE (2,2,3,4,4-D5)			324.1	306.4	10	22

Table S9. List of analysed steroids. Optimized MRM transitions and retention times for steroid standards and internal standards. These metabolites were chosen according to[4-6].

Table S10

Alpha-Linolenic Acid	11,12-DiHETrE	Eicosapentanoic acid	Gamma-LinolenicAcid	arachidonic acid	14,15-DiHETrE
12-KETE	8,9-DiHETrE	15-HEDE	15-KETE	13-KODE	9-KOTrE
9-HODE	9-HETE	14,15-DiHETE	8-HETrE	9-HOTrE	20-HETE
PGE2	PGB2	18-HEPE	13-HODE	12,13-DiHOME	10,17-DiHDoHE
17(18)-EpETE	5-iPF2a-VI	17-HDoHE	8,15-DiHETE	EKODE	15(S)-HETrE
PGE1	5,15-DiHETE	6-trans-LTB4	8-iso-PGF2a	15-HEPE	PGD1
Linoleic acid	docosahexaenoic acid	9(10)-EpOME	17,18-DiHETE	5-KETE	11-HEDE
7,17-hydroxy DPA	9-KODE	12(13)-EpOME	9,10-DiHOME	5-HETrE	13-HOTrE(gamma)
14-HDoHE	13,14-Dehydro-15-keto-PGE2	15-HETE	19,20-DiHDPA	12-HETE	LXB4
5,6-DiHETrE	11-HEPE	LTB4	11-HETE	13-HOTrE	9-HEPE
11-HDoHE	PGF2a	4-HDoHE	12(S)-HHTrE	PGD2	12-HEPE
5-HEPE	8-HDoHE	8-HETE	15-epi-LXA4/LXA4	8-HEPE	5-HETE

Table S10. List of analysed eicosanoids. These metabolites were chosen according to[7].

Fig. S1

We selected time windows associated with multisensory responses compared to unisensory responses. We combined EEG data from both cohorts and applied the cluster-based, non-parametric statistical procedure. Multisensory near responses differed from tactile only responses from 89 to 400ms ($P < 0.001$) and multisensory far responses were distinct from tactile only responses from 87 to 400ms ($P < 0.001$).

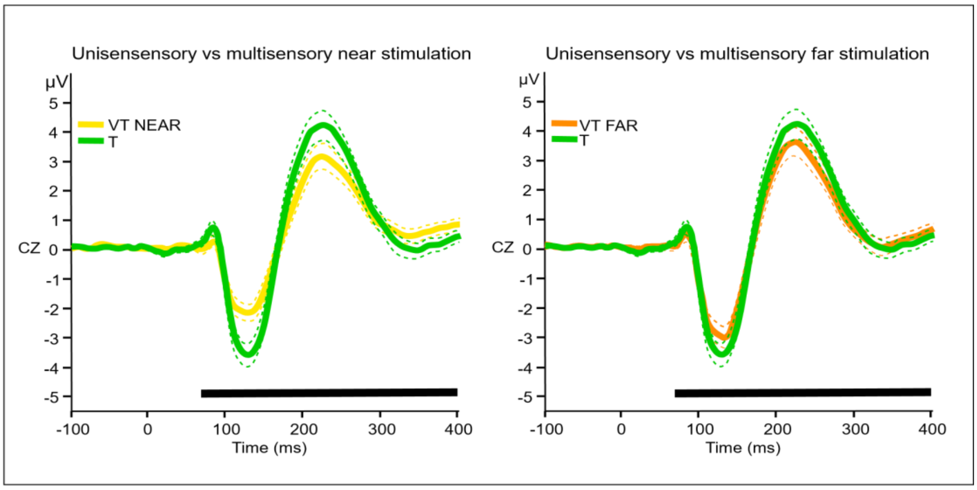

Fig. S1. Event related potentials showing distinct responses for unisensory (tactile = T) compared to visuo-tactile near (VT NEAR; Left) and visuo-tactile far (VT FAR; Right) stimulation. EEG data of both cohorts (control, infection) is shown together. The black segments on the bottom indicate significant time points from the whole period (from -100 to 400ms) and all electrodes (128) for the comparison between tactile only and visuo-tactile near and visuo-tactile far conditions. The black segment indicates epoch of significant difference between condition resulting from non-parametric analyses (and permutation tests to correct for multiple comparisons). Electrode CZ is shown as a paradigmatic example to illustrate different ERPs across conditions.

Figs. S2-S3

We tested whether the electrophysiological correlates of the PPS were distinct when a neutral or an infectious avatar was presented in both time windows showing a PPS effect (t1 = 87-168ms; t2 = 185-246ms). In the infection cohort, the contrast [Near (Neutral – Infectious) – Far (Neutral – Infectious)] revealed a significant GFP modulation between 129 and 150ms in the first-time window ($p < 0.05$, FDR corrected), while no significant effect was found in the second time window. No significant GFP modulation was found in the control cohort with the contrast [Near (Neutral – Neutral 2) – Far (Neutral – Neutral 2)] in either time windows (Fig. S2). These results demonstrate a distinct electrophysiological PPS effect when a neutral or an infectious avatar was presented. The comparisons between the two sets of neutral avatars in the control cohort showed no significant GFP difference (Fig. S3).

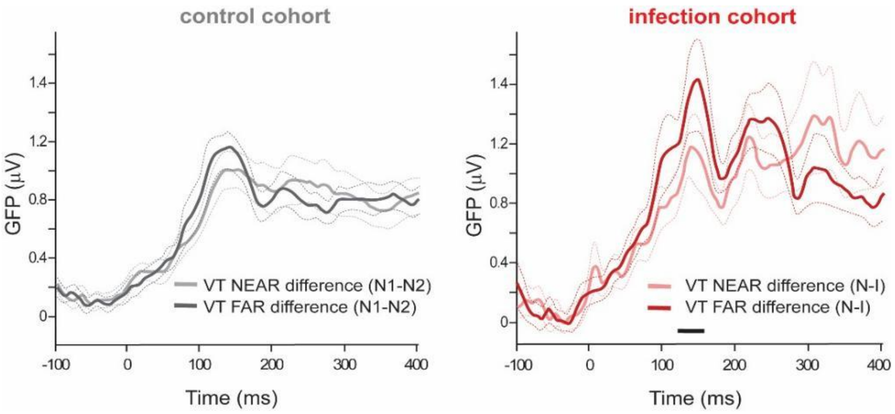

Fig. S2. Differential PPS responses for infectious and neutral avatars. The left plot shows the GFP difference between first and second block of exposure to neutral faces in the near (light grey) and far (dark grey) distance, demonstrating no differences in the control cohort. The right plot shows the GFP difference from the exposure to neutral and infectious faces in the near (light red) and far (dark red) distance.

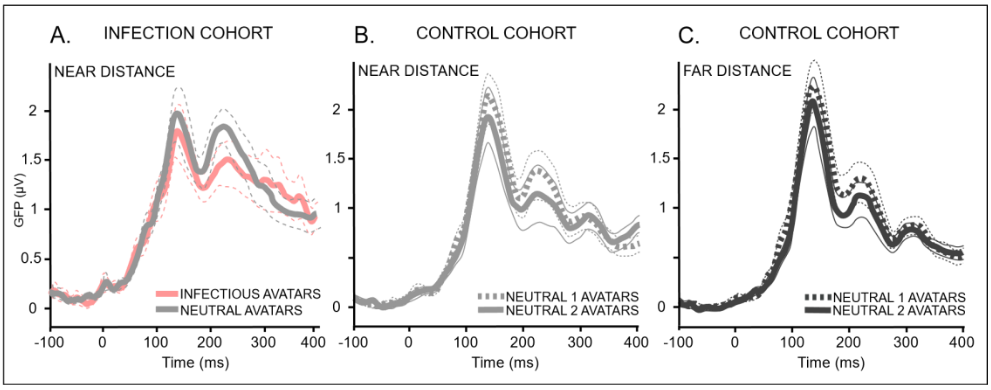

Fig. S3. Control comparisons. A) Comparison in the infection cohort between GFP evoked by infectious and neural avatars presented in the near space. **B-C)** Comparison in the control cohort between GFP evoked the two sets of neutral avatars (Neutral and Neutral 2) presented in the **B)** near and **C)** far space.

Fig. S4

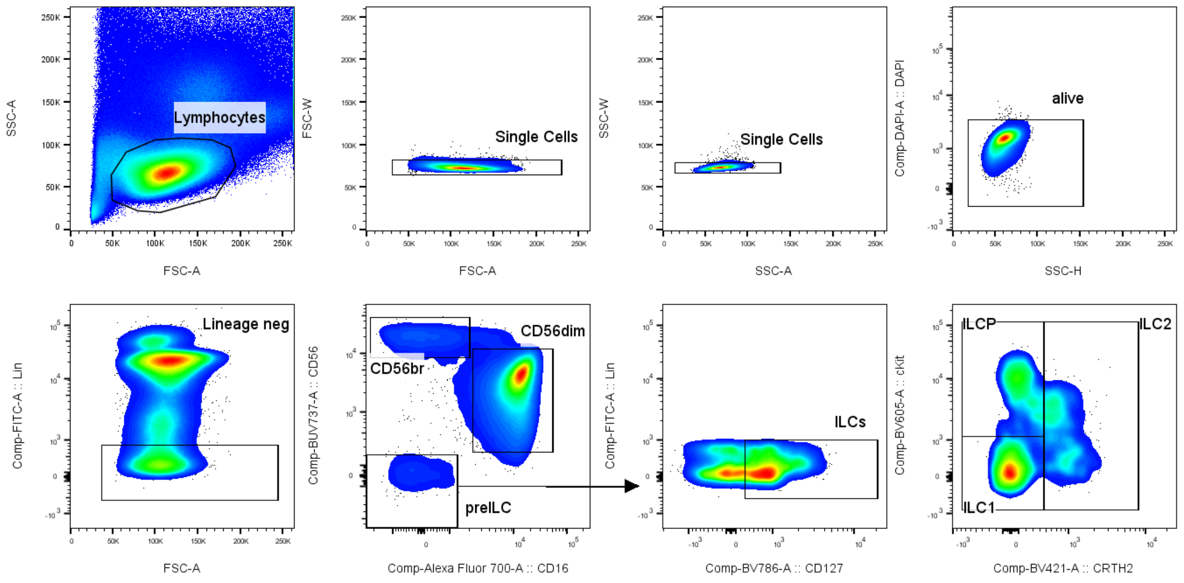

Fig. S4. Gating strategy to identify NKs and ILCs in flow cytometry.[8] NKs and ILCs were identified in the $FSC^{low}SSC^{low}$ lymphocyte gate. After doublets' exclusion, living cells were considered as DAPI negative. From the lineage negative (Lin^-) living lymphocytes, according to the expression of CD16 and CD56, NK^{bright} (CD56br) were identified as $CD56^{bright}CD16^-$, NK^{dim} (CD56dim) as $CD56^{dim}CD16^+$ and pre-innate lymphoid cells (preILC) as $CD56^-CD16^-$. From the preILC, total ILCs (ILCs) were identified as Lin^-CD127^+ cells. According to the expression of CRTH2 and cKit, ILC1s were gated as $CRTH2^-cKit^-$, ILC2s as $CRTH2^+cKit^{+/-}$ and ILCPs as $CRTH2^-cKit^+$.

Figs. S5-S7

We used principal component analyses on ILC frequencies and activation markers (for all types of ILCs combined). In both cases, the first PCA component explained most of the variance (90 % and 55 % for frequencies and activations respectively) and was therefore deemed sufficient for further analyses. ILCPs and ILC2s have a strong positive loading on the first PCA component, while ILC1s have a strong negative loading. This is reflected in the structure of correlations between these variables, in which ILCPs and ILC2s are strongly positively correlated, while strongly anti-correlate with ILC1s. All the loadings of the first component of the PCA on ILC markers are positive, therefore the component consists of a positively weighted average of all the markers. This is reflected in the strong positive correlations between almost all the activation markers. A similar pattern was observed for NK^{dim} and NK^{bright} activation markers (Figs. S5-S6), although the first principal component explained significantly.

To ensure that the first components of PCAs analysed were stable despite the relatively small sample size and high number of input variables, we used bootstrapping to estimate random variability due to sampling. We took 100 samples of 30 subjects (with replacement) from the neutral and infectious cohorts to simulate samples of participants from the population distribution. We applied PCA to each resample and computed the loadings of the first component. We then applied these loadings to the original sample of 30 participants, and computed the correlation of first components obtained through this procedure across resamples. This procedure was applied to ILC frequencies and activations, HPA-related hormones, eicosanoids, and neuroinflammatory factors. The average Pearson correlation coefficient was greater than 0.95 for all the five PCAs considered (see Figs. S5 panels G-J and S20 panels D-I).

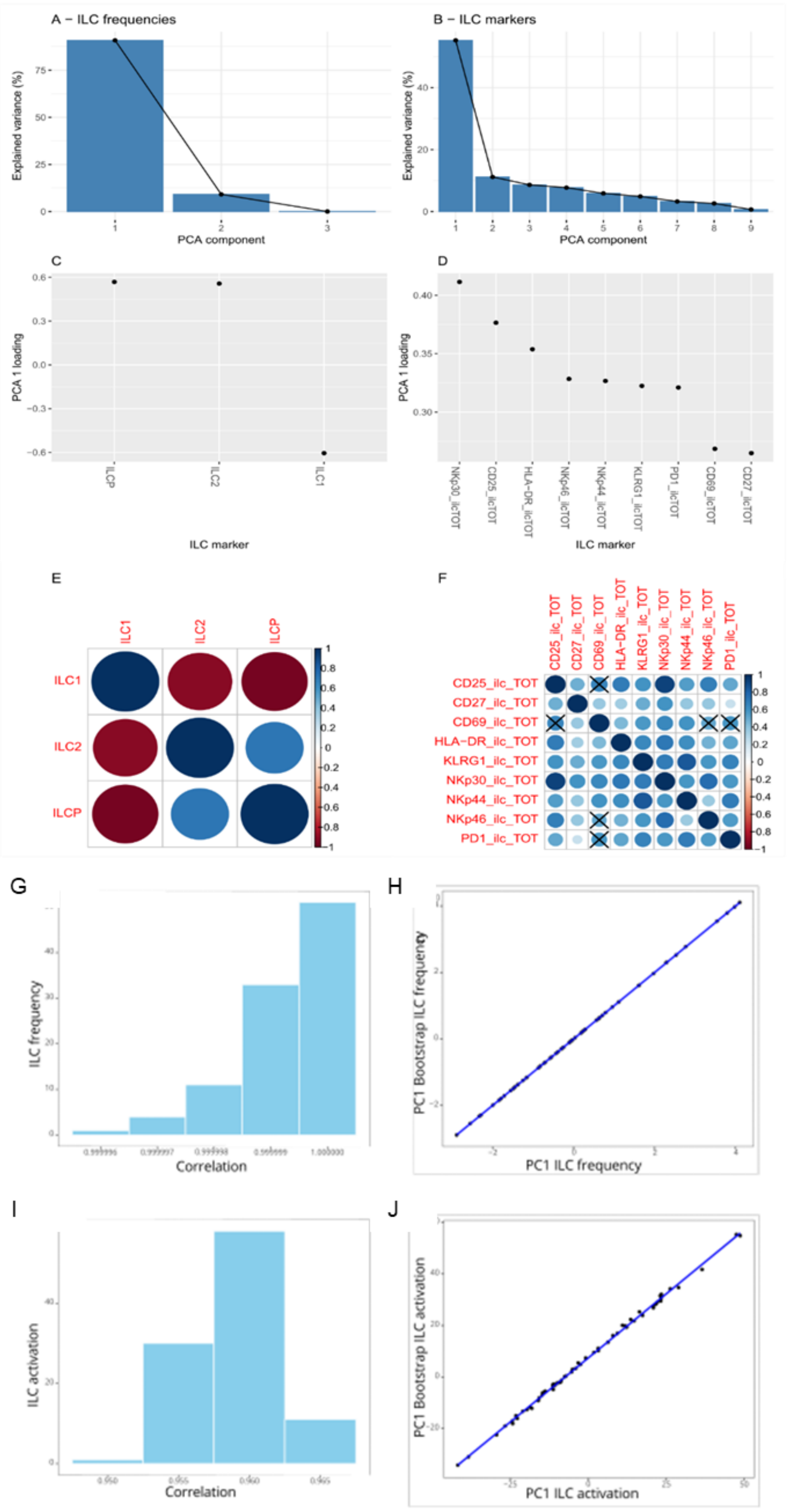

Fig. S5. Dimensionality reduction of ILC frequencies and markers. (**A, B**) Explained variance by the different PCA components for ILC frequencies' (**A**) and markers' (**B**) changes. (**C, D**) Loadings on the first PCA component for ILC frequencies' (**A**) and markers' (**B**)

changes. (**E, F**) Correlation matrix for ILC frequencies' (**E**) and markers' (**F**) changes. Crosses indicate non-significant correlations ($p > 0.05$). Stability of PCA components was confirmed for both ILC frequencies (**G, H**) and ILC markers (**I, J**), showing in left panels the histogram of correlation coefficients for the first component across resamples for each of the two PCAs considered, and showing in right panels an example correlation between the original first principal component, and the first principal component computed based on loadings from a resample.

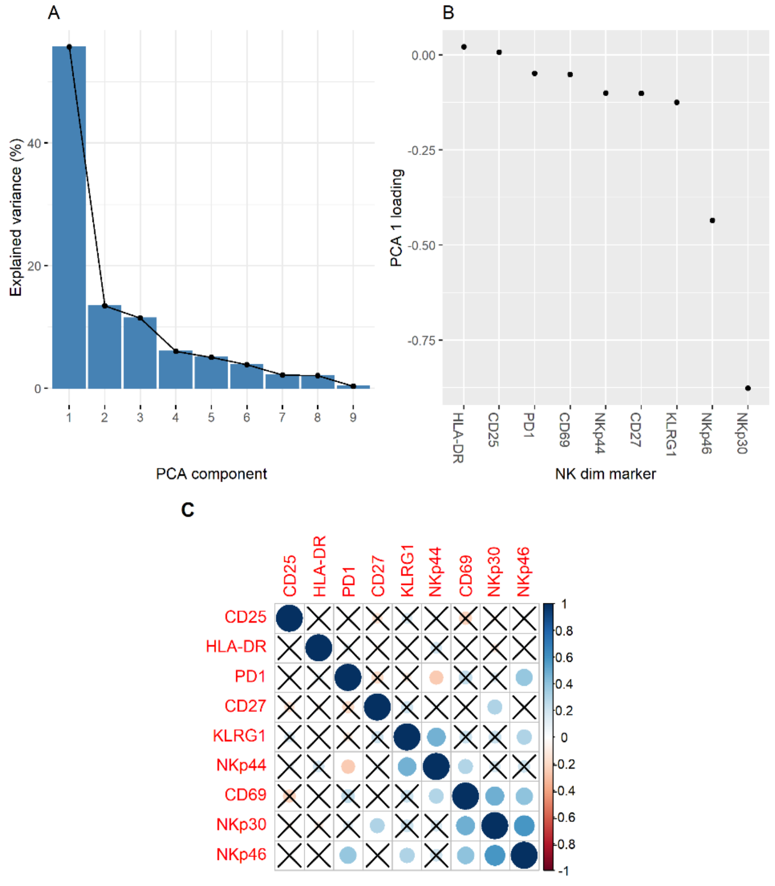

Fig. S6. Dimensionality reduction of NK^{dim} markers. (**A**) Explained variance by the different PCA components for NK^{dim} markers' changes. (**B**) Loadings on the first PCA component for NK^{dim} markers' changes. (**C**) Correlation matrix for NK^{dim} markers' changes. Crosses indicate non-significant correlations ($p > 0.05$).

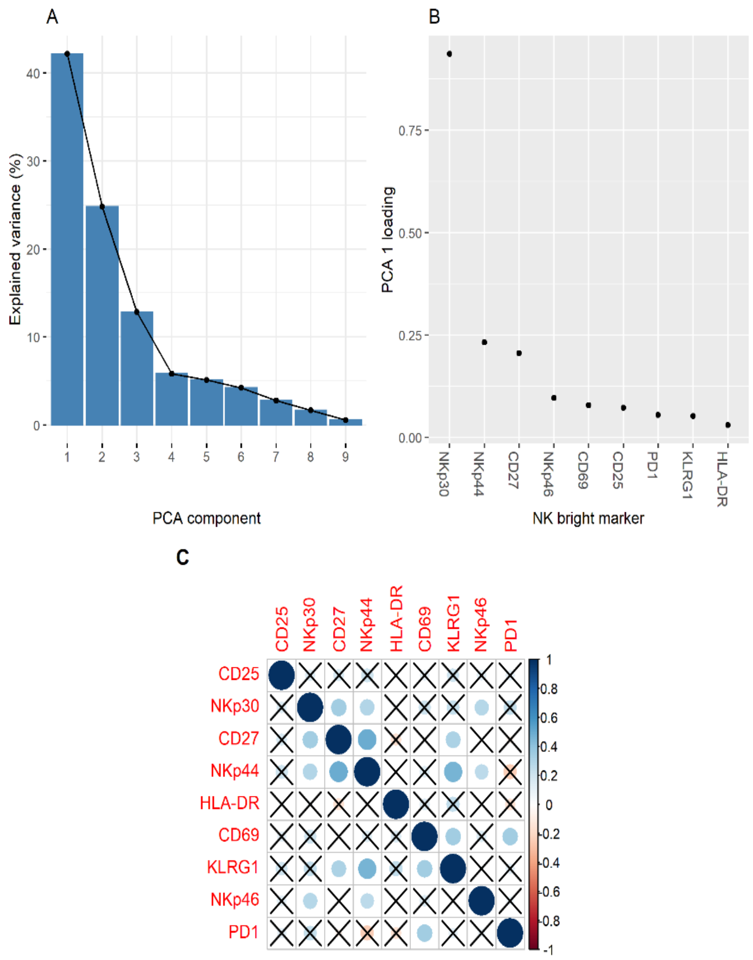

Fig. S7. Dimensionality reduction of NK^{bright} markers. (**A**) Explained variance by the different PCA components for NK^{bright} markers' changes. (**B**) Loadings on the first PCA component for NK^{bright} markers' changes. (**C**) Correlation matrix for NK^{bright} markers' changes. Crosses indicate non-significant correlations ($p > 0.05$).

Fig. S8

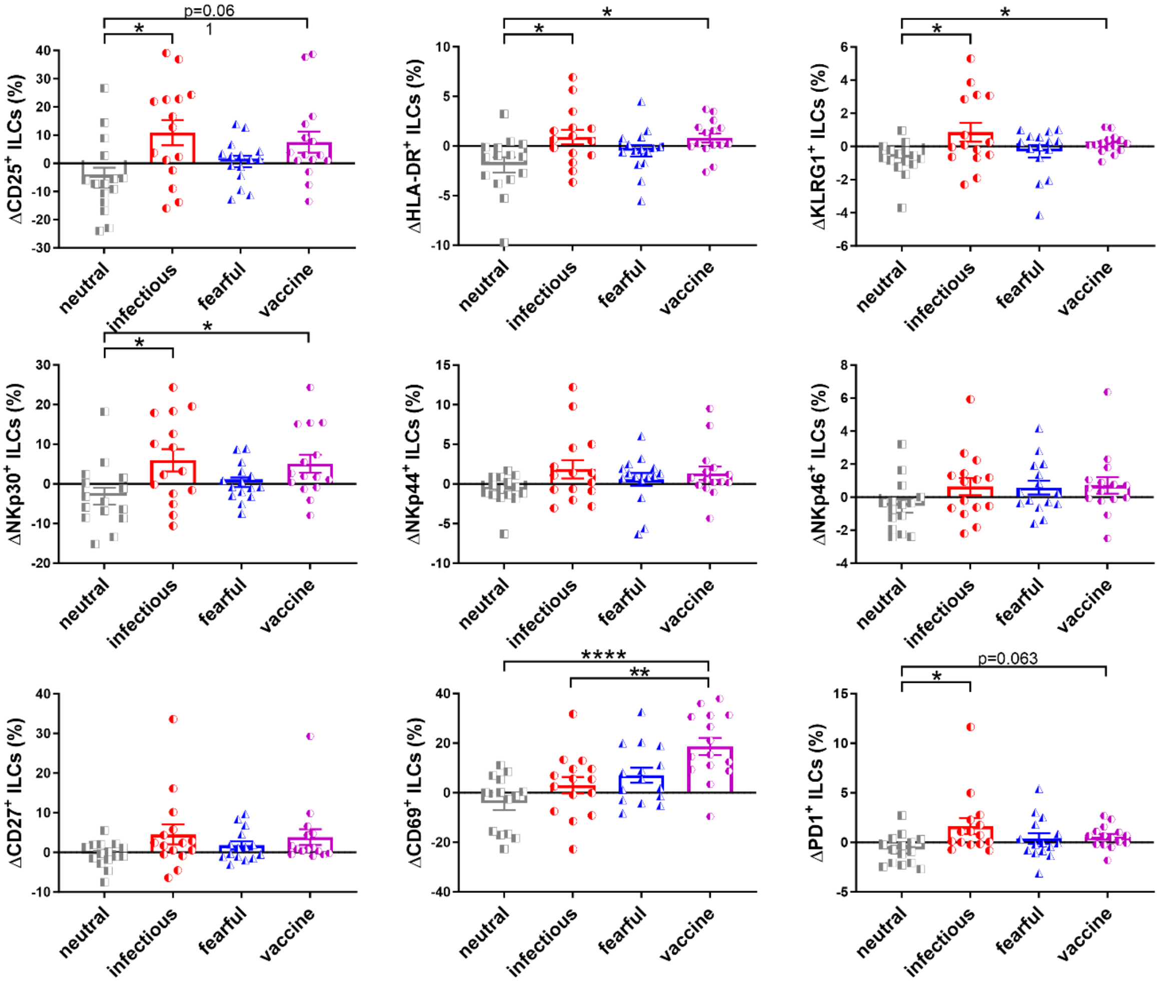

Fig. S8. Expression of activation markers on the ILCs of the 4 cohorts. The activation status of ILCs was evaluated through the expression of well-known activation markers, i.e., CD25, CD27, CD69, HLA-DR, NKp30, NKp44, NKp46, KLRG1 and PD1. Beside the significant modulation of CD25 (K=9.418, p=0.0242; neutral vs infectious p=0.021), HLA-DR ($F(3,56)=4.075$, p=0.0109, $\eta^2=0.1792$; neutral vs infectious p=0.014, neutral vs vaccine p=0.019), KLRG1 (K=8.880, p=0.0309; neutral vs infectious p=0.04, neutral vs vaccine p=0.048), NKp30 ($F(3,56)=3.759$, p=0.0157, $\eta^2=0.1676$; neutral vs infectious p=0.019, neutral vs vaccine p=0.041), and PD1 (K=10.09, p=0.0178; neutral vs infectious p=0.012) on ILCs, we did not observe a significant difference for the expression of CD27, NKp44 and NKp46 on ILCs (t-test, all p-values>0.099). Data are presented as the difference between the marker expression frequency after the second session VR exposure and the baseline (Δ).

Figs. S9-S13

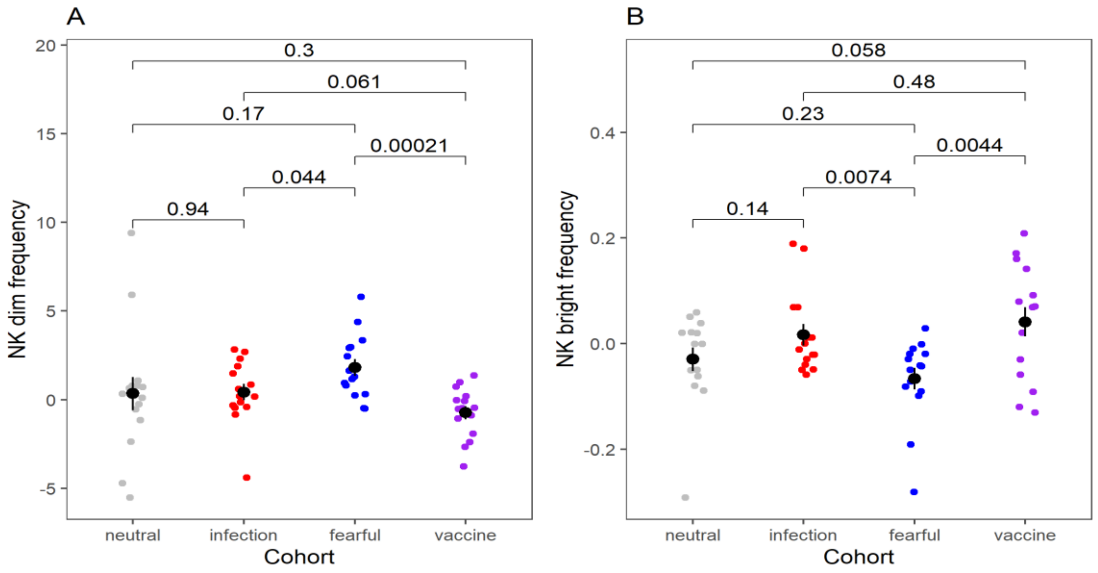

Fig. S9. NK^{dim} and NK^{bright} frequency in the 4 cohorts. (A) NK^{dim}, **(B)** NK^{bright}. The frequency and activation of NK^{dim} and NK^{bright} cells were analysed at baseline and after the second VR stimulation (or at the same time delay for the vaccine cohort). Data are presented as the difference between the cell subset population frequency after the second VR exposure and the baseline (Δ). For NK cell distribution, no differences were found for the single comparisons with respect to the neutral cohort (t-test, all p-values > 0.058).

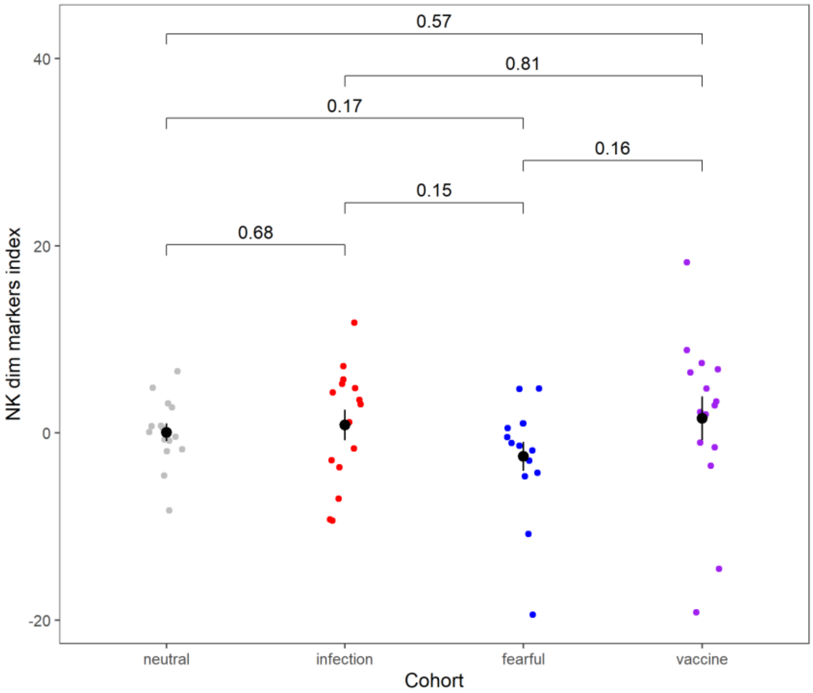

Fig. S10. Synthetic index NK^{dim} activation changes (first PCA component).

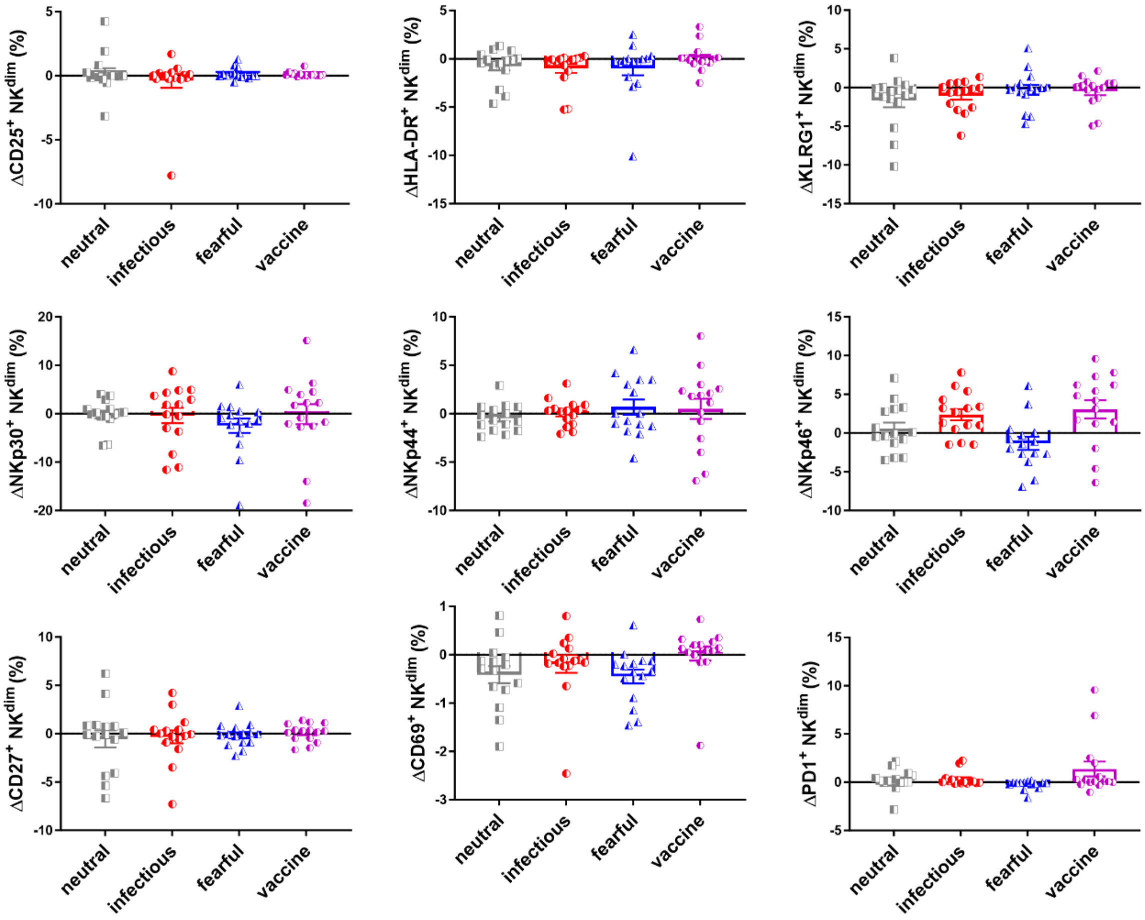

Fig. S11. Expression of activation markers on the NK^{dim} of the 4 cohorts.

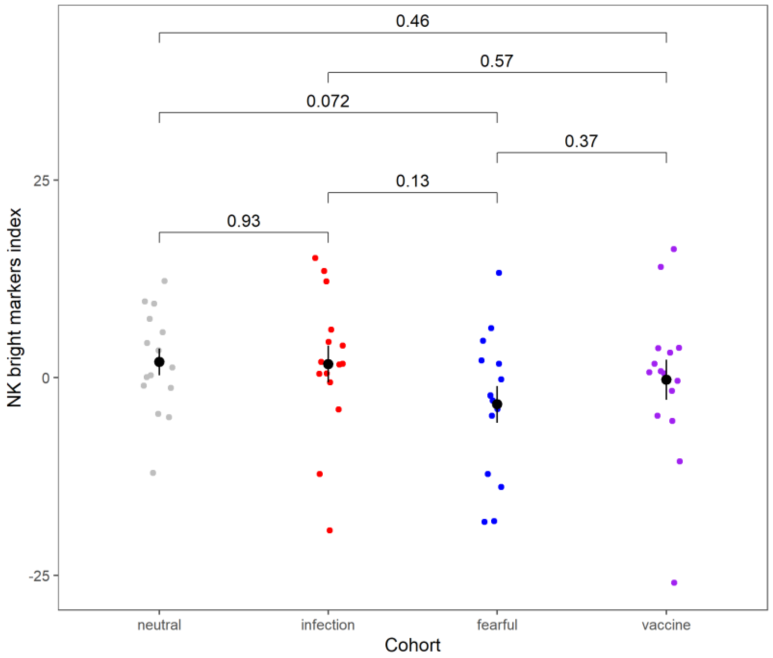

Fig. S12. Synthetic index NK^{bright} activation changes (first PCA component).

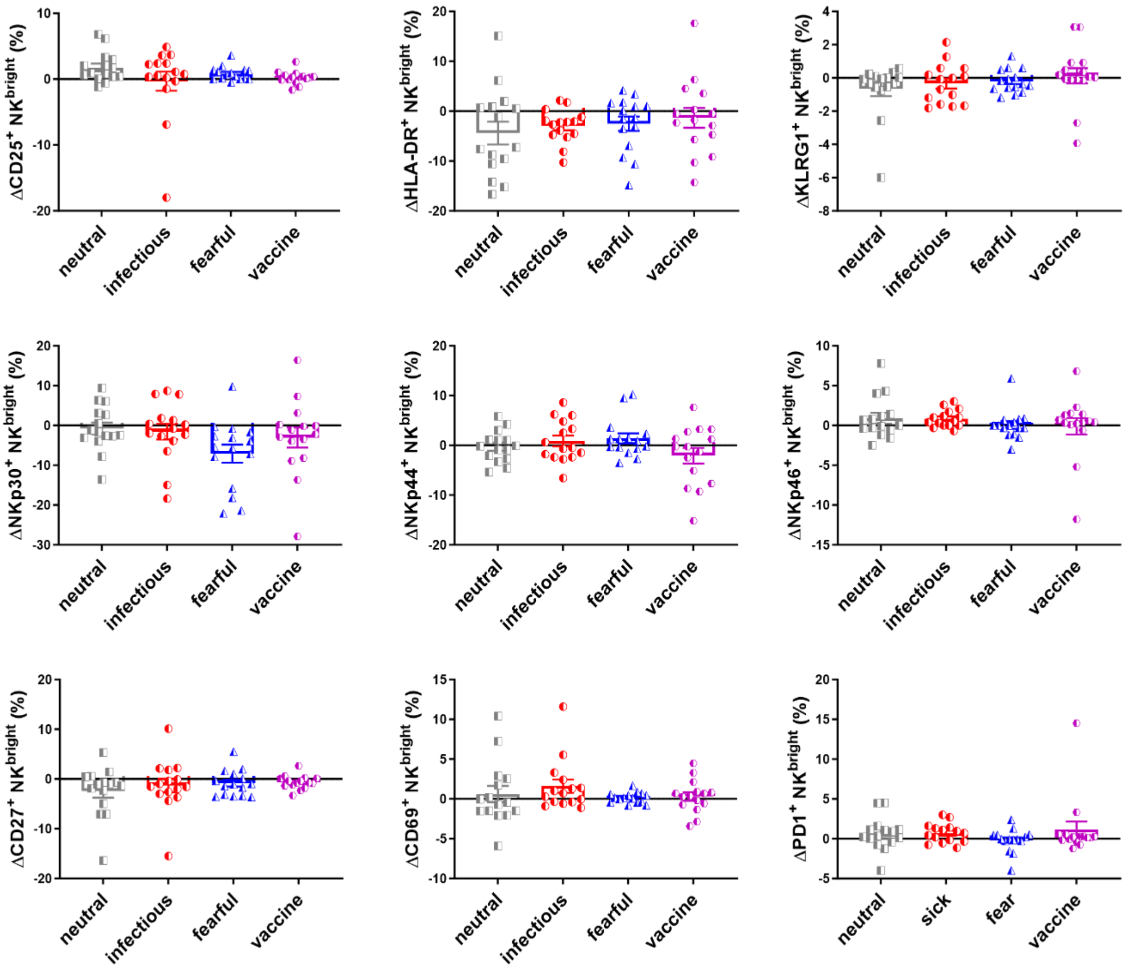

Fig. S13. Expression of activation markers on the NK^{bright} of the 4 cohorts.

Fig. S14

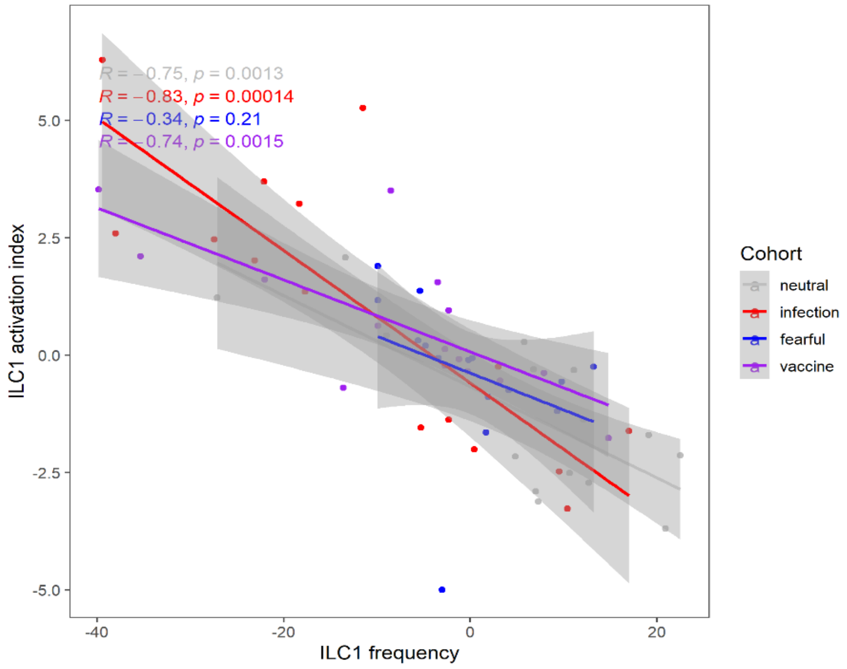

Fig. S14. ILC1 frequency and activation are inversely correlated. Correlation between the synthetic ILC1 frequency and activation indexes, with different colours denoting the different cohorts. Shades indicate the 66% confidence interval for the linear regression of each cohort.

Fig. S15

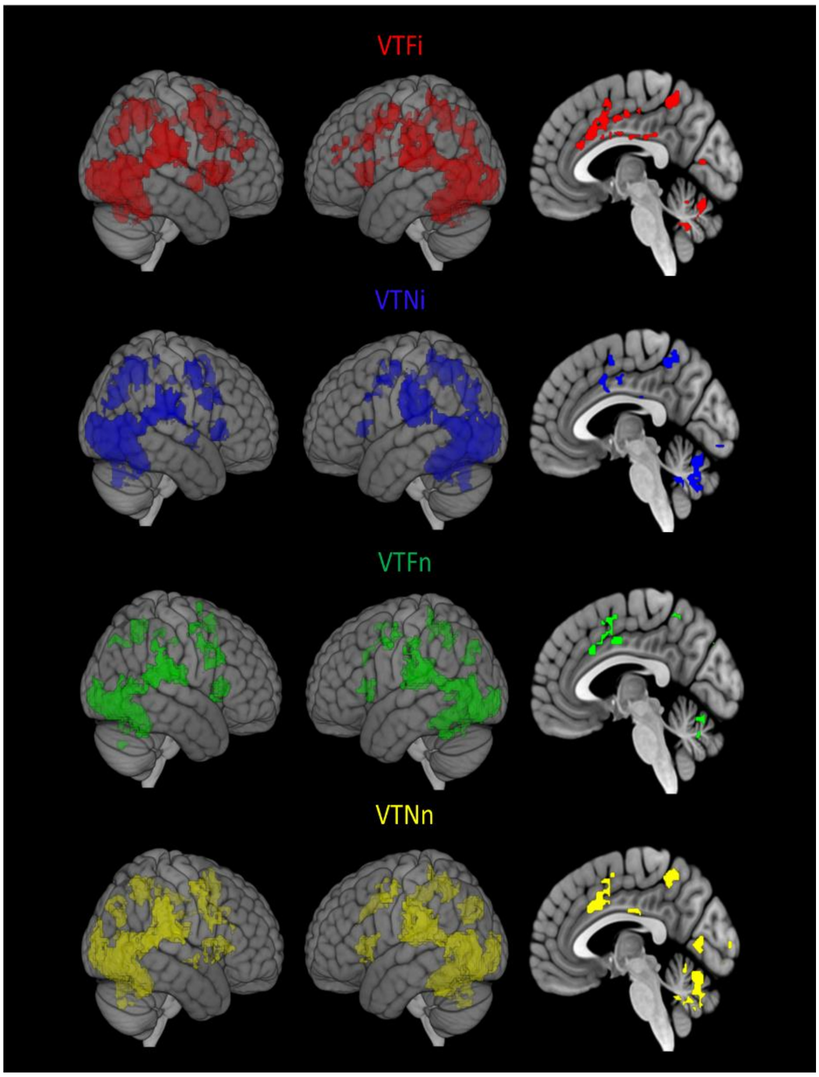

Fig. S15. fMRI activations during conditions VTFi, VTNi, VTFn and VTNn in the infectious group. Results showed common activations for all conditions in PPS-related areas including occipital areas, temporal areas, parietal areas and frontal areas. These results demonstrate the recruitment of PPS-related areas for both near and far conditions, as well as for both infectious and neutral avatars.

Fig. S16

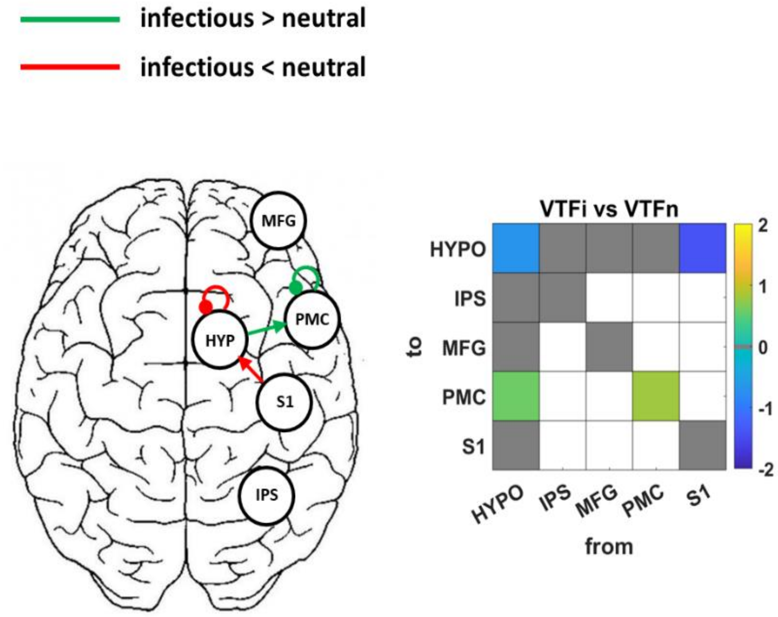

Fig. S16. Modulation of hypothalamic connectivity in the right hemisphere during exposure to infectious avatar in far space. Results showed an increase in connectivity from the hypothalamus towards PMC during conditions with infectious avatars compared to neutral avatars, while connectivity from S1 towards the hypothalamus was reduced. In addition, self-inhibition in PMC was increased while self-inhibition in the hypothalamus was reduced during conditions with infectious avatars compared to neutral avatars. Importantly, these hypothalamic modulations were specific for the far conditions and for infectious avatars, as we did not find any modulation when replicating the same analyses for conditions where avatars were presented in the near space nor where fearful avatars were presented.

Fig. S17

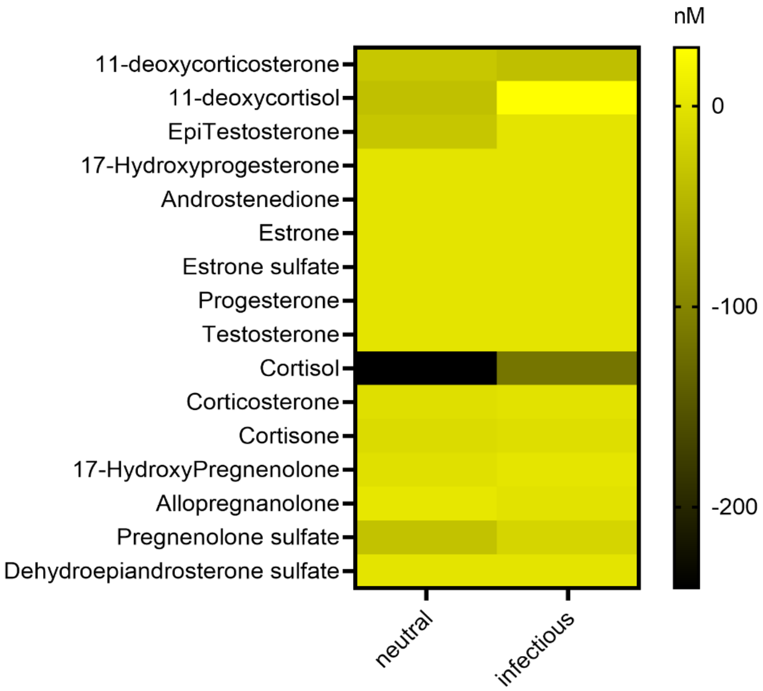

Fig. S17. Quantification of steroids in the sera of the neutral and the infection cohorts. Serum samples obtained from the subjects of the neutral and the infection cohorts were tested for the presence of different steroids that could link the neural with the immune activation. The heatmaps show the difference between the soluble mediators' concentrations after the second and the first VR exposure. While specific steroid species showed significantly different concentrations (cortisol, T test: p=0.0164; corticosterone, Mann-Whitney test: p=0.0453; pregnenolone sulphate, Mann-Whitney test: p=0.0186 and dehydroepiandrosterone sulphate, Mann-Whitney test: p=0.0408), for others no significant difference was observed (all p-values >0.11 except for Estrone: p=0.064). Steroids reported in Table S9 and not present in the heatmap were below the limit of detection.

Fig. S18

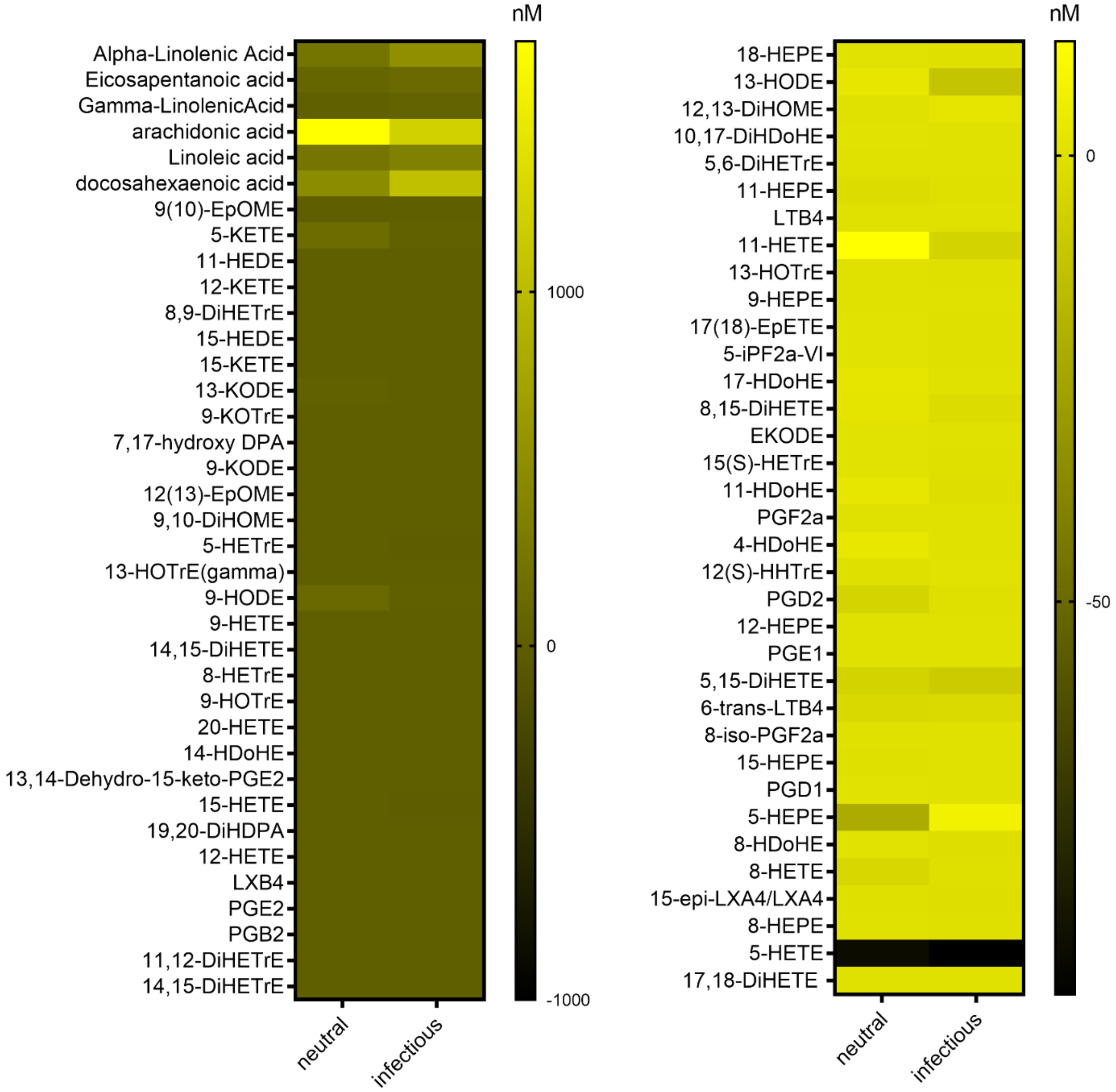

Fig. S18. Quantification of eicosanoids in the sera of the neutral and the infection cohorts. Different eicosanoids, as key inflammatory mediators that could link the neural with the immune activation, were quantified (reported in Table S10) in the serum samples obtained from the subjects of the neutral and the infection cohorts. The heatmaps show the difference between the soluble mediators' concentrations after the second and the first VR exposure. Beside the different concentration of some eicosanoids (11,12-DiHETrE (T test, p=0.0071), 14,15DiHETrE (T test, p=0.0453) and 17,18-DiHETE (T test, p=0.0064)), we observed no difference for other eicosanoids (all p-values >0.09 with the exception of 5-KETE: p=0.0186; 15-KETE: p=0.0556; 14,15-DiHETE: p=0.0694; LTB4: p=0.0767; 17(18)-EpETE: p=0.0418; 15(S)-HETrE: p=0.0664; 15-epi-LXA4/LXA4: p=0.0647).

Fig. S19

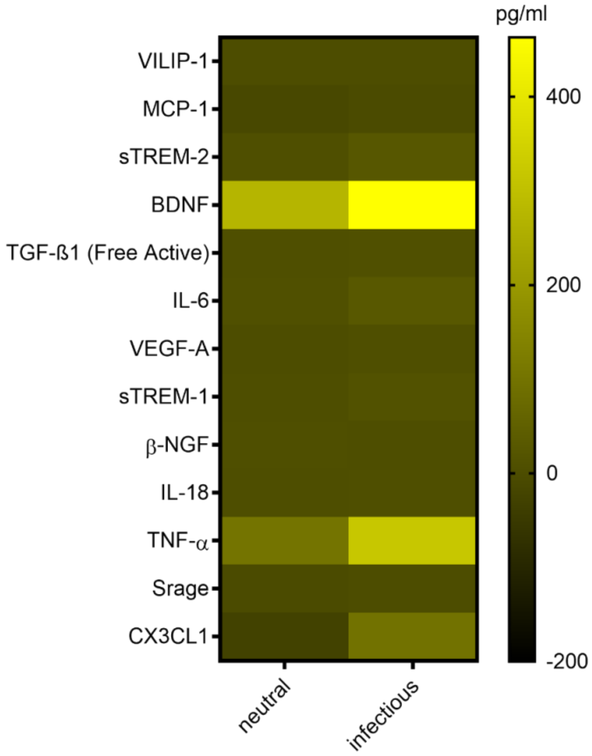

Fig. S19. Quantification of neuroinflammatory factors in the sera of the neutral and the infection cohorts. The serum obtained from the subjects of the neutral and the infection cohorts was also tested for the presence of neuroinflammatory factors. The heatmap shows the difference between the soluble mediators' concentrations after the second and the first VR exposure. Beside the different concentration of some mediators (CX3CL1 (T test: p=0.0264), sTREM1 (T test: p=0.0415), IL-6 (T test: p=0.0412)), we observed no difference for others (all p values >0.25, apart from IL-18 (p=0.097).

Figs. S20 and S21

Due to the large number of markers compared to the number of donors, we started by reducing the dimensionality of the input data by running a PCA on each of these three families of markers, similarly to what done to extract ILC frequency and activation indexes. In the case of neuroinflammatory factors, where data is available to the vaccine and fear cohorts, the PCA was run on all the cohorts. Estrone, Estrone sulphate, Hydroxyprogesterone, and Epitestosterone had to be excluded from the HPA-related hormone set, as they included below detection limit values. X14.15.DiHETE was also excluded for the same reason from the eicosanoids pool. Therefore, 71 eicosanoids, 11 hormones and 13 neuroinflammatory factors were included in our PCAs. As visible in Fig. S20, the first PCA component explained 39 % of the total variance for eicosanoids, and 33 % and 35 % for neuroinflammatory factors and HPA-related hormones respectively. Since in all the second and further PCA components explained less than half the variance explained by the first component, we retained only the first component of each family of markers as an input for the neural network. The vast majority of loadings on the first component has a positive sign for all three marker families (Fig. S21). Therefore, almost all markers of one family contribute with a different weight, but always positively, to their respective first PCA component. This makes the interpretation of the results more intuitive.

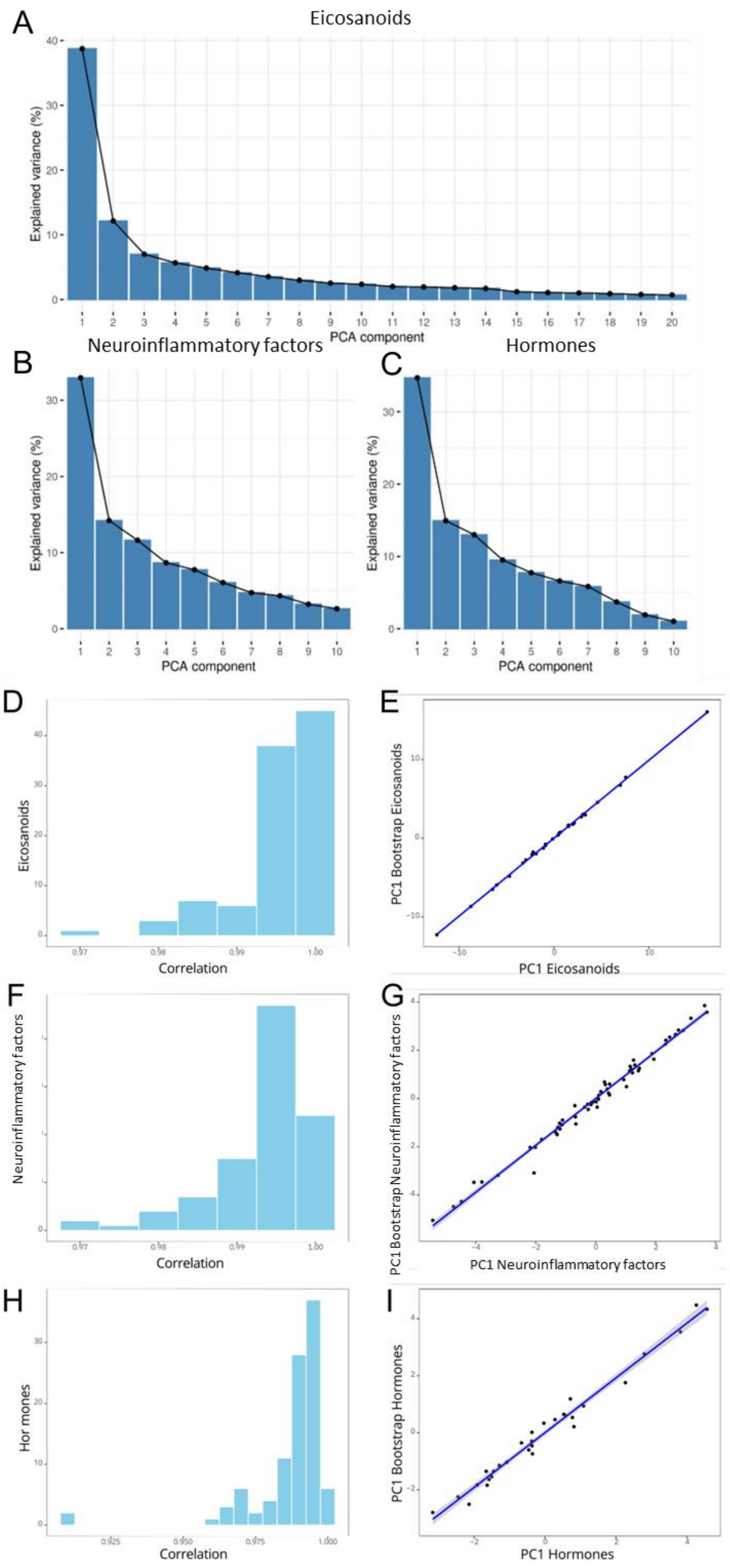

Fig. S20. Dimensionality reduction of serum multiOMICS. (**A, B, C**) Explained variance by the different PCA components for eicosanoids (**A**), neuroinflammatory factors (**B**), and HPA

related hormones (**C**) changes. Stability of PCA components was confirmed for eicosanoids (**D, E**), neuroinflammatory factors (**F, G**), and HPA related hormones (**H, I**), showing in left panels the histogram of correlation coefficients for the first component across resamples for each of the five PCAs considered, and showing in right panels an example correlation between the original first principal component, and the first principal component computed based on loadings from a resample.

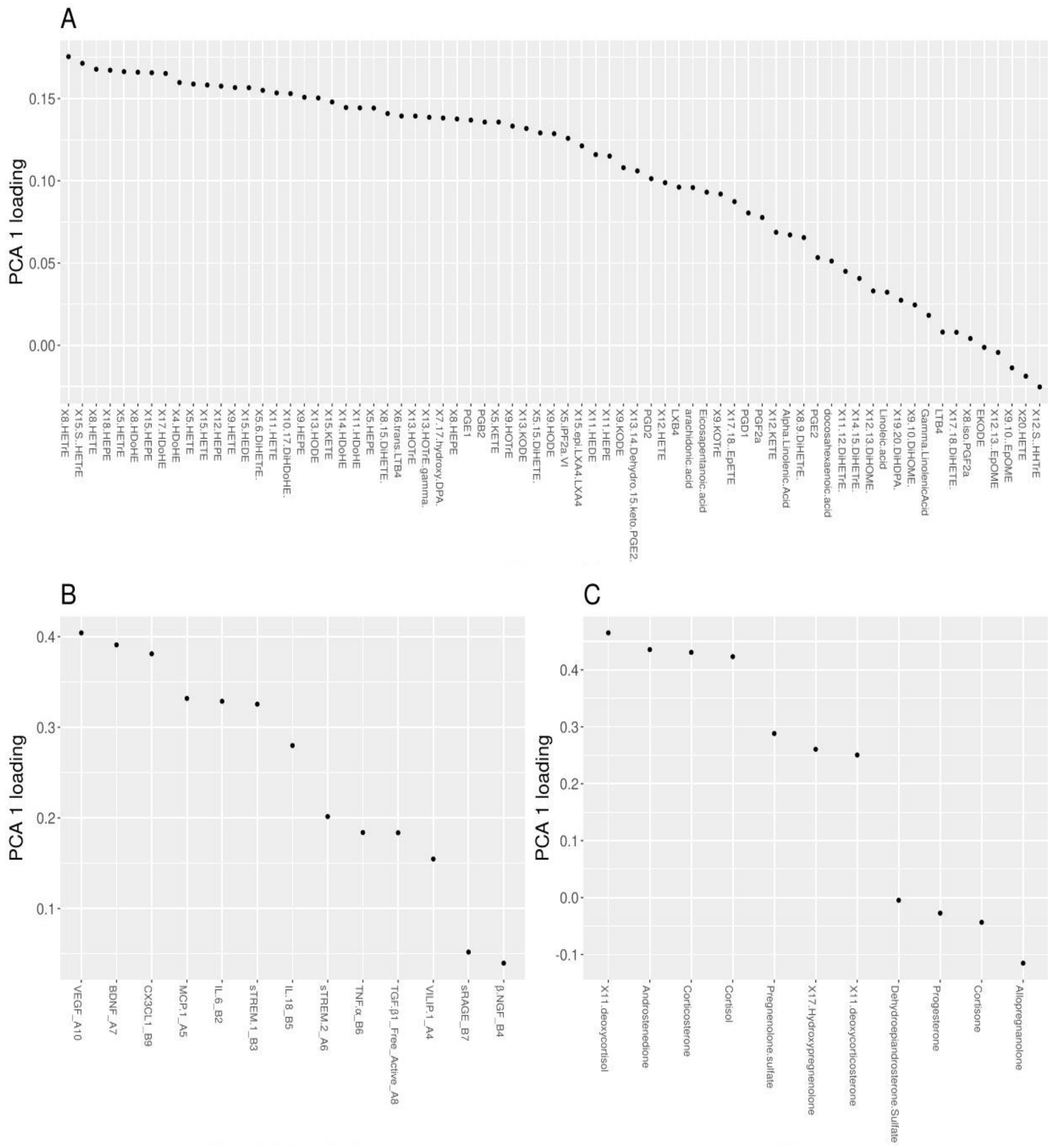

Fig. S21. Loadings of serum multiOMICS PCAs. (**A, B, C**) Loadings on the first PCA component for eicosanoids (**A**), neuroinflammatory factors (**B**) and HPA-related hormones (**C**) changes.

Fig. S22

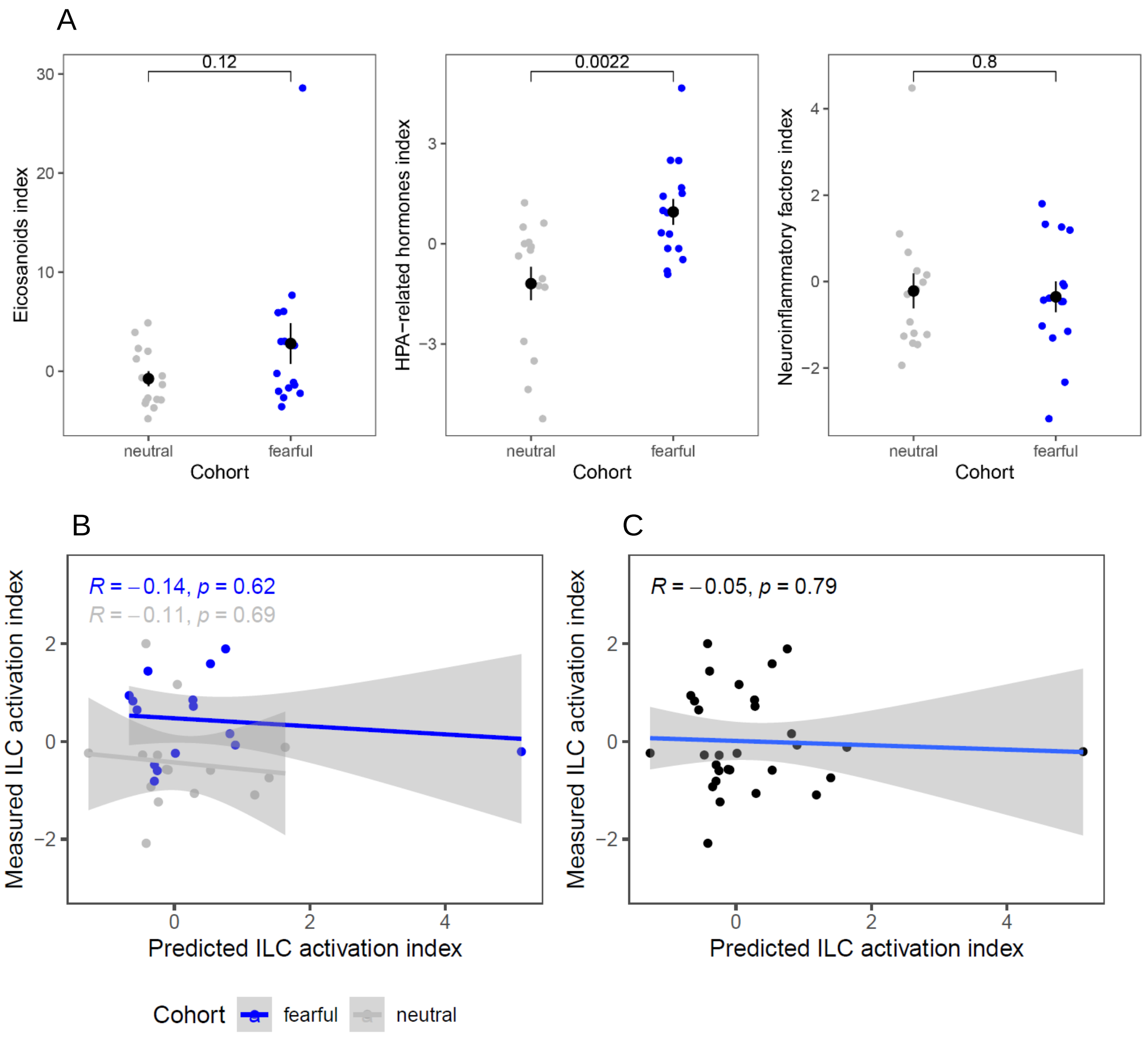

Fig. S22. Control neural network on neutral and fearful cohorts. Neural network analyses of the neutral and fearful cohorts. Neural network parameters were the same as described in the main text (i.e., eicosanoid, HPA-related hormones and neuroinflammatory factor indexes, panel (A)). Panel (B) shows the two cohorts separately, and panel (C) presents the same results for the two cohorts together. As expected, the analysis yielded no results, suggesting the neural signalling pathway highlighted for the infectious cohort is not activated by fearful avatars.

Fig. S23

To control for a potential effect of disgust in our results (e.g., an increase in brain activity when disgust is experienced), we computed an additional group-level comparison with contrast 3 by including as covariate an assessment of sensitivity to disgusting stimuli. We found a significant effect of disgust in the precuneus, the cerebellum and the left middle temporal gyrus (MTG). We note that none of these areas overlap with our previous results. In addition, even when accounting for disgust (i.e., removing the variance associated with disgust), the previous results were preserved (not shown). In addition, we also included disgust as covariate in our effective connectivity analyses (DCM) and found no significant effect associated with disgust.

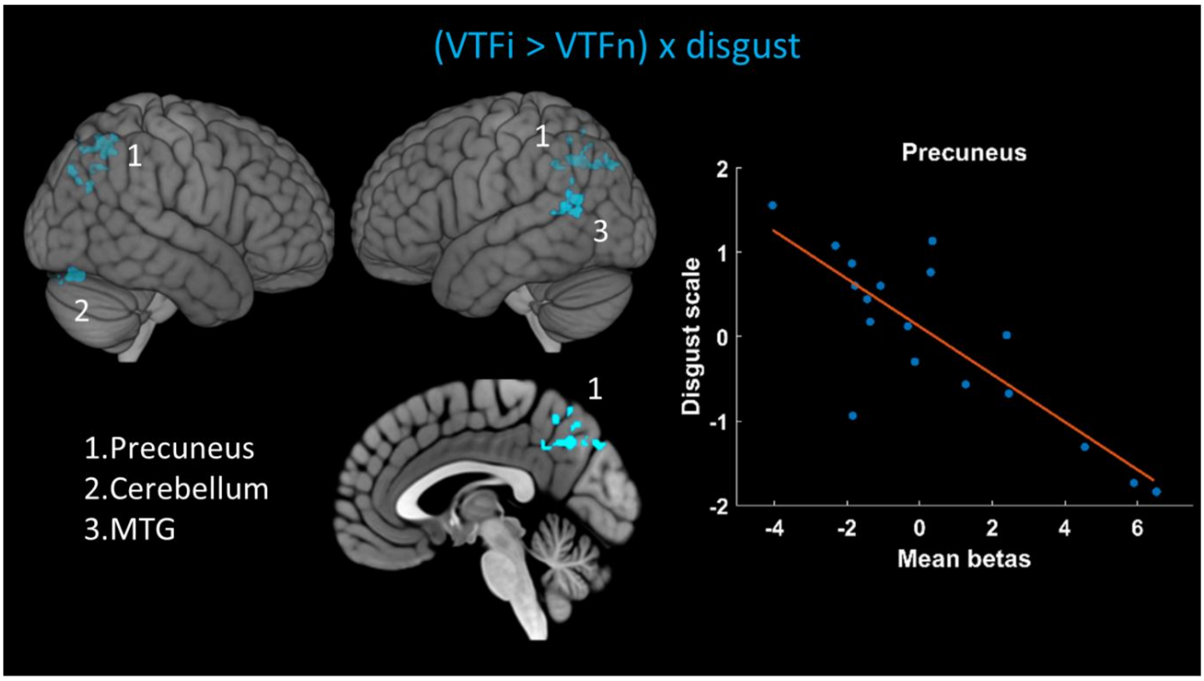

Fig. S23. Effect of disgust. Activations (infectious > neutral) modulated by sensitivity for disgust with a negative linear relationship between activation and disgust ratings in the bilateral precuneus, right cerebellum and left middle temporal gyrus.

Supplementary Methods

1. Visual stimuli

To select avatars with comparable intensity of perceived sickness, 23 participants [11 females; mean age 28.70 ± 4.52 (range 23–41)] were invited to rate the infectious avatars, shown on a computer screen, for how strongly the faces looked sick and contagious on a 10-point Likert scale (0 = not sick at all, healthy; 10 = sick and contagious). With this validation procedure, three female and three male infectious avatars were selected according to their highest perceived sickness score (mean female avatars= 6.40 ± 2.48; mean female avatars 6.80 ± 1.80; mean female avatars= 6.59 ± 2.18; mean male avatars= 7.44, ± 2.15; mean male avatars= 7.02 ±0.87; mean male avatars= 6.95 ± 2.21). Then, to control for potential differences in arousal evoked by the preselected avatars, 18 participants [9 females, mean age 27.33 ± 3.74 (range 20-35)] were invited to rate on a 7-point Likert Scale the level of "intensity" associated to the faces shown on a computer screen (0= not intense at all; 7 = extremely intense). For each face and gender condition separately (neutral, infectious, fear / male, female avatar), we computed average scores of the three preselected faces (see above) and compared these with non-parametric tests. A Friedman test showed that the arousal rating was significantly different across face conditions (female avatars: $X^2{}_F\,(18,2) = 26.17$, $P < 0.001$; male avatars: $X^2{}_F\,(18,2) = 28.22$, $P < 0.001$). Post-hoc tests using a Wilcoxon signed-rank test showed that neutral faces generated less arousal compared to infectious (female avatars: Z=3.59, $P < 0.05$; male avatars: Z=3.72, $P < 0.05$) and fearful ones (female avatars: Z=3.72, $P < 0.05$; male avatars: Z=3.72, $P < 0.05$). In contrast, similar levels of arousal were associated to infectious and fearful faces (female avatars: Z=1.21; male avatars: Z=1.81). Thus, fearful avatars were considered as a "control" condition, generating similar arousal effect as infectious avatars, but different level of contagion threat (explicit vs implicit threat).

2. Attitudes towards virtual infectious threats

This experiment was run to measure both implicit and explicit approach vs avoidance attitudes towards the infectious avatars. The experiment followed a within-subject design in which participants responded to an Implicit Association Test (IAT),[9,10] then performed an explicit rating of the avatar's faces along six dimensions and finally completed the seating distance scale.[1] The IAT consisted of seven-blocks and was designed to assess the differential association of infectious ("sick") and neutral ("healthy") faces with an approach vs. avoidance dimension (see Table S2). The single categorization task served as practice task, whereas the combined task as test phase to reveal and assess participants' implicit associations. Stimuli in the IAT consisted of twelve virtual faces developed for the PPS paradigm (six infectious avatars resulting from the stimuli selection and six neutral healthy avatars) and 10 words related to approach (approach, closer, touch, attraction, getting closer) or avoidance (stay away, distance, avoid, avoidance, repulsion). The first two blocks were single categorization task: participants were asked to categorize via a button press approach vs. avoidance words (36 trials), and then healthy and sick faces (36 trials). Then, the first of the two combined tasks were presented. This consisted in a familiarization phase (36 trials) followed by a block (96 trials) in which participants reaction times (RT) were registered. Then the procedure was repeated and the responses to the "healthy" vs. "sick" faces were reversed. A single categorization task (36 trials)

was presented to prepare participants to the last combined task (96 trials). The order of the combined task was counterbalanced across participants. In each block, the randomly selected stimuli were presented, one at the time (no replacement), in the centre of the computer screen and participants were asked to categorize each stimulus as fast as possible using the left (D) and the right (K) key of the keyboard. The IAT was scored by the D algorithm[10], such that more positive scores indicated stronger associations between healthy faces and approach words (and sick faces and avoidance words) than the reverse combination.

Then, participants rated each of the six neutral and six infectious avatar faces - randomly presented - on the following attributes: realistic, pleasant, unpleasant, healthy, and sick on a 7-point scale. If the face was judged somehow "sick" (response 2 or greater), participants also evaluated its perceived contagiousness.

Finally, a graphic version of the seating distance scale[1] was administered as a measure of the preferred interpersonal distance. The participants' task was to choose the chair where they would like the target to be seated among seven others that differed in terms of distance (1 = closest, 7 = furthest) from the chair they were supposedly seated in. In different conditions, the target was one of the six infectious or the six neutral avatars.

3. Behavioural responses to virtual infectious threats entering the PPS

Visual stimuli consisted of different virtual faces, described above. The experiment was implemented using a custom-made experimental software (ExpyVR, available online at http://lnco.epfl.ch/).

At the beginning of each trial, an avatar's face appeared in one of three possible, counterbalanced positions: centrally on the visual field, on the left or on the right periphery with ±10° visual angle off the centre. The face moved in the sagittal plane for 3s from the initial far apparent position from the participant to a near position, where the face remained still for 1s before disappearing. While watching these looming avatars, participants were requested to respond as quickly as possible any time they felt a tactile vibration on their cheeks by pressing a button with their dominant hand. The task was composed of four blocks of 84 trials. Each block involved 40 trials in which a looming avatar was coupled with a tactile vibration (*visuotactile* trials). Tactile stimuli were delivered at five different delays following the onset of the visual stimulus (D5=0.5s; D4=1s; D3=1.5s; D2=2s; D1=2.5s). Correspondingly, the avatar's face was perceived at one of five distances (D1 ≈ 45 cm, the nearest; D2 ≈ 80 cm; D3 ≈ 115 cm; D4 ≈ 150 cm; D5 ≈ 185 cm, the farthest). At the start of each trial, the avatar's face appeared in one of three counterbalanced positions: centrally, on the left, or on the right periphery (10°). The face moved at a constant velocity of 0.70m/s in the sagittal plane, starting from an initial position approximately 2.20m away and moving to a position about 0.30m from the participant, where it remained still for 1s. Thus, the position of the face at the time of tactile stimulation appeared to be more proximal to the participant at increasing delays. In 20 trials, there was no avatar face (unimodal *tactile* trials), where tactile stimulation was delivered alone at one of the same five temporal delays. Another 20 trials were *visual only* trials, in which the looming avatar was presented without tactile stimulation, and participants were instructed to refrain from responding (*catch trials*). The remaining four trials were used as *attentional trials*, to confirm that participants were looking at the avatars' faces during the task. These trails consisted of avatars with a red dot on the forehead that participants were asked to detect by

signalling to the experimenter with a vocal response (no touch was delivered). Trials were presented randomly, separated by one of five jittered inter-trial intervals (0.2s, 0.4s, 0.6s, 0.8s or 1.0s). Each block lasted approximately 7 minutes and the experiment consisted in 2 blocks per condition.

The experiment followed a mixed-subject design in which participants were randomly assigned to one of three groups: infection cohort (N=15, 8 females, 7 males); neutral cohort (N=15, 8 females, 7 males) and fearful cohort (N=15, 8 females, 7 males). The experiment was divided into two sessions, composed of two blocks each. In the first, baseline session (block 1 and 2), all participants were exposed to neutral avatars. In the second session, participants were exposed either to infectious (“infection cohort”), fearful (“fearful cohort”) or neutral (“neutral cohort”) avatars, according to their assignment.

RT to tactile stimulation were analysed. RT slower than 1000 ms were discarded. Then, we excluded from the analyses RT higher or lower than 2.5 standard deviations for each block as outlier responses. In line with previous studies,[11] for each subject we computed a baseline-correction index of multisensory facilitation by subtracting the averaged RT in the unimodal condition from the mean RT in the multimodal condition at each distance (D1-D5), separately for each block. Consequently, all negative baseline-corrected RT indicated a multisensory facilitation.

First, using baseline-corrected RT, we checked that the visuo-tactile stimuli to neutral avatars presented in baseline session in each cohort were similarly processed. Statistically, there was no difference between the cohorts at baseline. An ANOVA with the factors distance (from D1 to D5) and cohort at baseline (first session) showed, as expected, a main effect of Distance ($F(4, 168)=28.9$; $p<.001$; $p\eta^2=0.41$), but no main effect of Cohort ($F(2,42)=1.08$; $p=.35$; $p\eta^2=0.049$) nor an interaction ($F(8,168)=1.77$; $p=.09$; $p\eta^2=0.078$).

Then, baseline-corrected RT were submitted to a repeated measures ANOVA with distance (D1; D2; D3; D4; D5) and session (pre-session; post-session) as the within-subject factors, and cohort (infection cohort; fearful cohort; neutral cohort) as a between-subjects factor. In all conditions, responses speeded as the virtual faces approached (main effect of distance: $F_{(4,168)}= 24.073$, $P <0.001$, $p\eta^2= 0.364$, Greenhouse-corrected), thus showing the predicted PPS-like facilitation effect[11]. Specifically, results obtained from one-sample t-test against 0 indicated that baseline-corrected RT were faster than unimodal RT from D1 to D4 ($P < 0.05$, FDR corrected), showing a spatially dependent multisensory facilitation, hereafter a PPS effect. Critically, the PPS effect varied between the baseline and the second session [session x distance: $F_{(4,168)} =4.332$, $P = 0.002$; $p\eta^2= 0.093$], but more importantly as a function of the presented avatar (three-way interaction; $F_{(8,168)} = 2.421$, $P=0.017$, $p\eta^2=0.103$). Accordingly, to identify the source of such interaction, we analysed results with separated repeated measures ANOVAs, one per cohort.

As regard to the neutral cohort, results indicate a significant main effect of distance [$F_{(4,56)}=8.532$, $P<0.001$, $p\eta^2=0.379$], but no main effect of session [$F_{(1,14)}=2.798$, $P=0.117$, $p\eta^2=0.166$] nor of the session X distance interaction. [$F_{(4,56)}=1.149$, $P=0.343$, $p\eta^2=0.076$]. One sample t-tests against 0 indicated that baseline-corrected RT were faster than unimodal RTs from D1 to D3 ($P < 0.05$¸FDR corrected). Thus, there was a PPS effect from D3 to the closest distance, which did not vary between the two sessions.

Results from the infection cohort indicated a main effect of distance [$F_{(4,56)}$=9.625, P<0.001 $p\eta^2$=0.401], no main effect of session [$F_{(1,14)}$=2.158, P=0.164 $p\eta^2$=0.134], and, more importantly, a significant interaction between session and distance [$F_{(4,56}$=3.063, P=0.024, $p\eta^2$=0.180)]. The significant interaction is explained by the fact that in the baseline session multimodal RT were significantly faster than unimodal RT only at D1 and D2 (one sample t-tests against 0, P < 0.05¸FDR corrected), while in the infection session they were significantly faster from D1 to D4 (one sample t-tests against 0, P < 0.05¸FDR corrected), indicating that the PPS effect occurred earlier, i.e., when an infectious threat was present even farther from the body.

Previous work suggested that a higher PPS segregation caused by COVID-19 lockdown was associated with increased fear of contamination.[12] Those data were collected immediately after the relaxation of the first lock-down measures, which had imposed strong social distance policies. Thus, the higher PPS segregation can be interpreted not as due to a generic fear to be contaminated by another individual, but as the consequence of interacting with other people from a much higher distance than before the lock-down, sharpening one's PPS to very closer distances. On the other hand, we interpret the effect triggered by infectious avatars as an anticipation of contact. Indeed, there is no correlation between the behavioural effect induced by infection faces and the Perceived Vulnerability to Disease scale in the present data (correlation with the slope of the RT by distance function: r=-0.21; p=.45), suggesting that the effects triggered by the COVID-19 lockdown and the present effects does not tap into the same mechanisms.

Finally, findings from the Fearful cohort indicated a main effect of distance ($F_{(4,56)=}$6.930, P<0.001 $p\eta^2$=0.331), but no main effect of session [$F_{(1,14)=}$1.541, P=0.235 $p\eta^2$=0.099]. A significant interaction session X distance was found [$F_{(4,56)}$=5.568, P<0.001, $p\eta^2$=0.285)]. The interaction depended by a significant difference between sessions only at D1, as shown by planned post-hoc comparisons (p<.05, corrected for 5 comparisons): RT at the closest location was faster for neutral than for fearful avatars. However, the boundary of PPS, i.e., the location at which a significant facilitation effect occurs, did not vary between the two conditions: visuo-tactile RT were significantly faster than unimodal RT (i.e., baseline at 0 in the graph) from D3 to D1 in both conditions (all p-values <.01). Thus, fearful faces affected multisensory integration within the PPS differently than neutral faces, only at the closest distance from the participant's body, without altering the PPS extent. Different responses to neutral and fearful faces at closer distances were reported also by Ellena and colleagues.[13] That effect however depended on the location of visual attention, as modulated by a concurrent fixation task. Here we did not modulate the focus of attention, thus we cannot further interpret these findings.

To summarize, an infectious face elicits a PPS effect at farther distances as compared to a neutral or a fearful face.

4. <u>EEG-adapted PPS task</u>

EEG experiment followed a within-subject design in which participants were randomly assigned to two independent groups: an infection cohort (N=16) and a control cohort (N=16). Data from two participants (one in each cohort) were discarded due to large degrees of electrical noise or technical problems during EEG recording. Thus, the final data set consisted of 30 participants: N=15 in the infection cohort, and N=15 in the control cohort.

We assessed the electrophysiological processing of PPS representation by adapting a modified version of the PPS task described in Experiment 2. Participants were sitting on a comfortable chair in a Faraday cage and were exposed to visual stimuli rendered in 3D with an Oculus Rift while tactile stimulations were applied on the participant's cheeks. We used the same tactile stimulations and avatars faces (neutral and infectious) as described in 3.1 and 3.2. However, to limit the experiment duration (given the number of trials per condition necessary for EEG analysis) and to control for the number of comparisons, avatars were presented only in the closest and in the farthest distances (two distances, D1 and D5). In order to still present dynamic stimuli, which are known to evoke stronger responses from PPS neurons,[11,14] while controlling for expectancy effects, the avatar face moved centrally on a sagittal plane for 1.5s either in the near or far space. Importantly, the size of the faces for the near and the far distances occupied the same visual angle.

Four types of trials were presented. During *visuo-tactile trials*, while the avatar face approached towards the participant in the near space (visuo-tactile near trials, N=28) or in the far space (visuo-tactile far trials, N=28), tactile stimulation was presented after 1s with a jitter of +-100ms (steps of 25ms, randomly presented). During *unisensory tactile trials* (N=28), tactile stimulation appeared after 1s with a jitter of +- 100ms (steps of 25ms, randomly presented), without avatar faces approaching. In the *unisensory visual trials,* avatar faces in the near space (visual near trials, N=28) or in the far space (visual far trials, N=28) moved centrally on a sagittal plane during 1.5s, without concurrent tactile stimulation. Attentional trials consisted of avatar faces (attentional near trials, N=4; attentional far trials, N=4) with a red dot on the forehead that participants were asked to detect by signalling with a vocal response (no tactile stimulation delivered). These trials aimed to keep participant's attention on visual stimuli. Thus, one block of the task included 148 trials with seven conditions presented randomly (visuo-tactile near; visuo-tactile far; unisensory visual near; unisensory visual far; unisensory tactile; attentional trials near; and attentional trials far) and lasted approximately 8 minutes. In total, participants were exposed to six blocks of the task, with 2-minute break between each block.

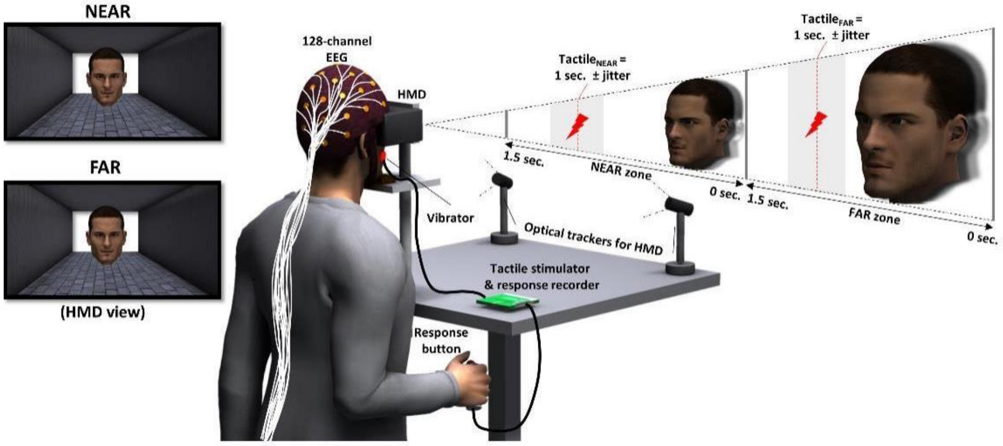

Fig. S24. Experimental setup and paradigm for the EEG study. Participants wearing 128-channel EEG rested their chins on a custom-made rigid structure that also held the head mounted display (HMD). In the Visual only or Visuo-Tactile conditions, they observed through the HMD an avatar face (sick or neutral) looming towards their own face either in the Near or

the Far zone for a duration of 1.5s. In the Visuo-Tactile conditions, at 1s ± jitter after the appearance of the looming face, participants received simultaneously a tactile stimulation on their two cheeks using vibrators. The size of the avatar face was adapted to always cover the same visual angle, irrespective of whether it appeared in the Near or Far zone. The in-depth information was therefore provided by stereoscopy. A virtual hallway and centred fixation cross acted as static references to stabilize gaze.

The experiment started with a training block of four minutes in which participants were required to press a button as fast as possible upon receiving automatized mild touch to their cheeks while concurrently observing neutral avatars in mini-looming trials. The aim of the training block was to remove the novelty effect of the task and to associate implicitly tactile stimulations to a relevant event, as in the PPS task of Experiment 2. Then the experimental blocks started, and participants did not press the button anymore upon receiving tactile stimulations, to avoid any contamination by processes related to a decision to react or to withhold reaction.

The infection cohort was exposed to three blocks with neutral avatars (condition 1, *Neutral blocks*) and three blocks with infectious avatars (condition 2, *Infection blocks*), while the control cohort was exposed to three blocks with neutral avatars (condition 1, *Neutral blocks*) and three blocks with different neutral avatars (condition 2, *Neutral 2 blocks*). All blocks were presented in alternated order.

4.1 EEG acquisition

Continuous EEG was acquired with a sampling rate of 1024 Hz through a 128-channels Biosemi ActiveTwo system (Biosemi V.O.F., Amsterdam, Netherlands) referenced to the common mode sens/driven right leg (CMS-DRL) ground. The horizontal and vertical electrooculograms were recorded by attaching additional electrodes at the lateral canthi of both eyes and below the eyes, respectively. The EEG data was preprocessed and analysed using MATLAB (R2017a; The MathWorks), EEGLAB toolbox41, and Cartool software42. Data were first re-referenced to average reference, down-sampled to 512Hz, notch filtered at 50 Hz and bandpass filtered from 0.1 Hz to 40 Hz using a 4th order bi-directional zero-phase infinite impulse response (IIR) filter. Epochs from -100 to 400ms relative to tactile stimulation onset were then extracted for each condition separately. Major artifacts (eye blinks and noisy data segments) were screened and removed manually. Noisy channels were selected manually for interpolation with the data from adjacent channels. EEG data was transformed using independent component analysis, and SASICA43 was used to guide the exclusion of independent component associated to noisy channels, eye movements, blinks and muscular contractions. On average, we excluded 11.6 (+-2.1) and 12.2 (+-1.6) independent components in the control and the infection cohort, respectively (see Supplementary Method 4 for EEG adaptation of PPS task and analysis).

4.2 EEG analyses

Statistical analyses were performed in two steps. First, we identified time windows responding to multisensory (visuotactile, VT) stimuli (vs. unisensory tactile, T). Then, within these time windows, we characterized distinct PPS responses for neutral and infectious avatars. In the first step, we compared multisensory (VT near and VT far) to unisensory (T) responses (Fig. 1D). To increase the power of this analysis, for each distance (near, far), we combined EEG data of condition 1 (neutral avatars) and condition 2 (neutral avatars in the control cohort and

infectious avatars in the infection cohort) of both cohorts (control, infection). Significant differences between T and VT (near, far) were determined with a cluster-based, non-parametric statistical procedure as implemented in the Fieldtrip toolbox.9,10 This data-driven approach controls for the false positive error rate in a situation of multiple comparisons (multiple time points and electrodes). Significant time windows (between -100ms to 400ms) in the contrast VT versus T were considered as multisensory responses and were selected for the second step of the analyses. In the second step, we conducted analyses based on the classic approach to study PPS,[3,15,16] in which PPS is defined as a multisensory modulation of tactile stimulation due to an external stimulus (here visual presentation of avatars), as a function of the distance of these stimuli from the body in space. We first estimated the PPS distance effect (VT near versus VT far) with EEG data from both cohorts together (Fig. 1E). Then, we tested whether the PPS response was distinct when a neutral or an infectious avatar was presented, by using the contrast [Near (Neutral – Infectious) – Far (Neutral – Infectious)] in the infectious cohort, and [Near (Neutral – Neutral 2) – Far (Neutral – Neutral 2)] in the control cohort (Fig. 1F-H). All statistical analyses in the second step were performed on the Global Field Power (GFP). GFP has the advantage to represent a measure of the neural strength of evoked responses while attempting to reduce the inherent high dimensionality of EEG data (false positive).[17]

To localize neural activity in key contrasts, we performed a current density analysis in 3D Tailarach/MNI space of the scalp-recorded electrical activity using the sLORETA/eLORETA software package.13 sLORETA estimates the distribution of electrical neural activity in 3D space, based on the measurements of a dense grid of 6239 voxels at 5mm spatial resolution, which are placed on the entire scalp surface covering the brain. This inverse solution algorithm assumes related orientations and strengths of neighboring neuronal sources (represented by adjacent voxels). For each time point, the voxel based sLORETA-images were compared between the conditions using the sLORETA-built-in voxel wise randomization tests (5000 permutations) based on t-statistical non-parametric mapping (SnPM),14 corrected for multiple comparisons. Figures 1E and 1H show the difference in estimated current distribution at the timepoint showing the strongest statistical difference within each significant time period.

5. <u>fMRI-adapted PPS task</u>

fMRI data acquisition followed a within-subject design in which participants (N=18) were exposed to both neutral and infectious avatars entering the PPS, in a pseudo-randomized design. In addition, another group of participants (N=20) were exposed to both neutral and fearful avatars entering the PPS.

The fMRI setup for the PPS task was composed of an MR-compatible VR-system to present the virtual avatars (resolution: 1920x1080, refresh rate: 60 Hz, https://nordicneurolab.com/visual-system-hd/) and a MR-compatible pneumatic stimulator to deliver tactile stimulation on the chin. The fMRI PPS task was adapted from the EEG PPS task (see section 4.4). As in experiment 4, we used the “mini looming” stimuli, whereby the avatar face moved centrally on a sagittal plane for 2.0s either in the near or far space. Because of the lower 3D resolution of the fMRI VR system, to improve the perception of depth and the associated near/far perceived positions, the size of the faces for the near and the far distances were kept at their original dimensions (i.e., the face in the near space appeared larger), differently from the EEG task where the size of the faces were adapted to occupy the same visual angle.

During visuo-tactile trials, while the avatar face approached towards the participant for 2.0s in the near space (visuo-tactile near trials VTN, 9 trials/run) or in the far space (visuo-tactile far trials VTF, 9 trials/run), tactile stimulation was presented after 1s, with a jitter of +- 100ms. During unisensory tactile trials (9 trials/run), tactile stimulation appeared after 1s, with a jitter of +- 100ms, without avatar faces approaching. In the unisensory visual trials, avatar faces in the near space (visual near trials VN, 9 trials/run) or in the far space (visual far trials VF, 9 trials/run) moved centrally on a sagittal plane during 2.0s, without concurrent tactile stimulation. Attentional trials consisted of avatar faces (attentional near trials, 1 trial/block; attentional far trials, 1 trial/block) with a red dot on the forehead that participants were asked to detect by signalling with a button response (no tactile stimulation delivered). These trials aimed to keep participant's attention on visual stimuli and were not included in the fMRI analyses. Thus, one run of the task included 47 trials with seven conditions presented randomly (visuo-tactile near; visuo-tactile far; unisensory visual near; unisensory visual far; unisensory tactile; attentional trials near; and attentional trials far) and lasted approximately 6 minutes. In total, each participant was exposed to four runs of the task, two runs with neutral avatars and two runs with infectious avatars, in a pseudorandomized order. Similarly, in the fearful cohort, each participant was exposed to four runs of the task, two runs with neutral avatars and two runs with fearful avatars, in a pseudorandomized order.
The experimental procedure started with a training block (in MR environment) of four minutes in which participants were required to press a button as fast as possible upon receiving automatized mild touch to their chin, while concurrently observing neutral avatars in mini-looming trials. The aim of the training block was to remove the novelty effect of the task and to associate implicitly tactile stimulations with a relevant event, as in the PPS task of Experiment 2. Then the experimental runs started, and participants did not press the button anymore upon receiving tactile stimulations (except for attentional control trials), to avoid any contamination by processes related to a decision to react or to withhold reaction.

6. <u>Neural network architecture and predictions</u>

The experimental data was fitted through a single hidden layer neural network, with 50 hidden units, biases and skip-layer connections. Sigmoidal activation functions were used except for the output layer, which was linear since the response variable is continuous. Input and output variables were not scaled since they already have close to zero mean and similar variance. This allows directly comparing network predictions and other data reported in the paper. To avoid overfitting, weight decay was used and set to 1, a value which (compared to the commonly used value of 0.1) considers the fact that unscaled input and output variables have variance larger than 1. To test the network's accuracy in predicting experimental data while controlling for overfitting, we used leave-one-out cross-validation. Predictions for each donor were generated by training the network on all the other donors, and the final network accuracy was computed as the correlation coefficient between predictions and actual data. To minimize variability due to stochastic variability in the network's training, we repeated the process 100 times and averaged the predictions over the iterations before computing the final results.
To interpret and visualize the relation between serum multiOMICS and immune response, uncovered by the neural network, we investigated its input-output mapping. We trained another neural network with the same parameters on all data and computed the predicted immune

response on a regular grid of values spanning from the minimum to the maximum value of each input variable. Since neuroinflammatory factors showed a simple, almost linear modulation of the predicted output, they were fixed at their mean values to provide an interpretable visualization in the main text (see Fig. 5D). The complete input-output relation for the whole range of neuroinflammatory factors is shown in Fig. S25. A "hotspot" of predicted immune response is present for all levels of neuroinflammatory factors, but the overall response reduces as neuroinflammatory factors increase.

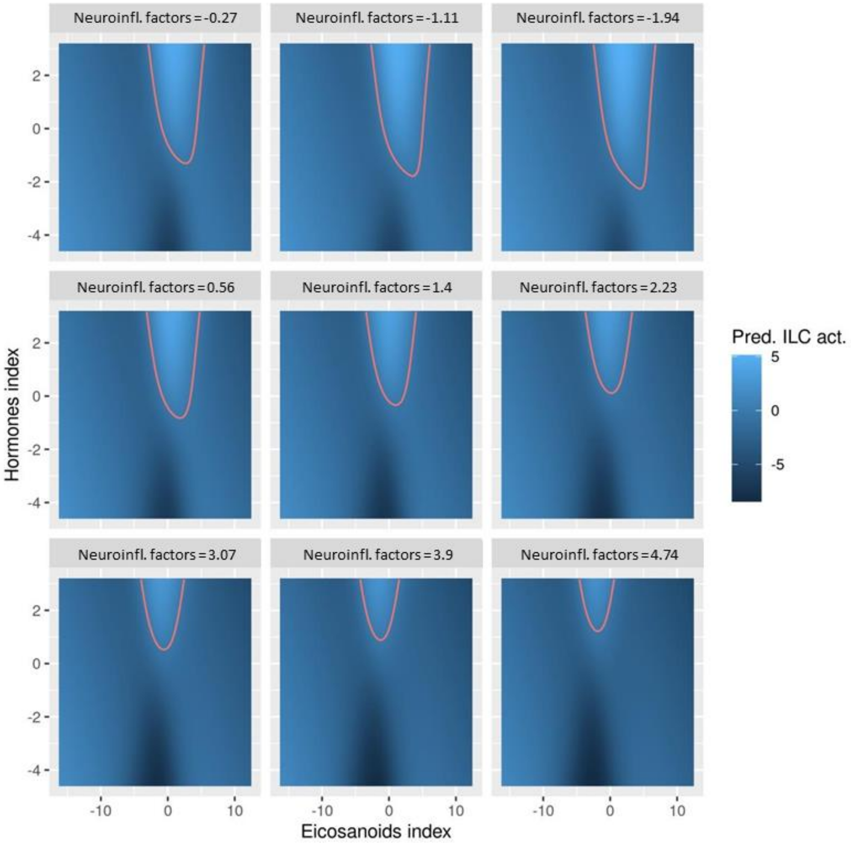

Fig. S25. Complete input-output relation of the ILC activation network. Input-output relation of the network trained on ILC activations, as a function of eicosanoids and HPA-related hormones, with each subplot representing a different level of neuroinflammatory factors.

We then investigated whether individuals exposed to the infectious avatars did indeed show levels of serum markers corresponding to such activation hotspot. We defined the hotspot as the region of the input variables inducing an immune response above the average of the neutral + infection cohorts. To be conservative, we excluded from the so defined hotspot the region at low eicosanoids, low hormones, in which the above average predicted response is mainly pulled by one single subject in the infection cohort. Then, we performed a binomial test to check whether subjects in the infection cohort were statistically more likely to fall within the activation hotspot. The test was significant with the selected threshold (T-test, $p = .008$), and remained significant across a broad range of thresholds used to define the activation hotspot (Fig. S26).

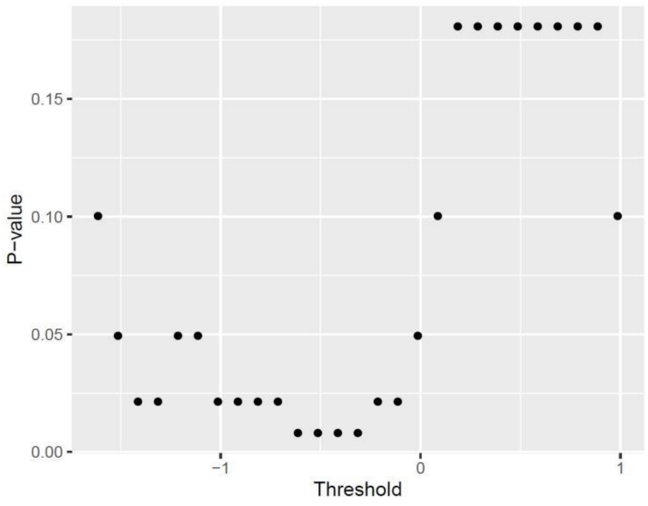

Fig. S26. Threshold sensitivity of the activation hotspot analysis. The graph shows the p value for the binomial test on the number of subjects from the neutral/infection cohort falling in the activation hotspot, as a function of the threshold used to define it. The threshold used in the main text (ILC activation index = -0.287) corresponds to the average ILC activation index, and the analysis yields significant results across a broad range of thresholds.

6.1 <u>Neural network prediction of ILC frequency from serum multiOMICS after exposure to infection avatars</u>

Finally, we tested the same network architecture and testing method on ILC frequencies instead of ILC activations. The network still provided significant predictions, although with moderate performances (R = 0.42, p = 0.022).

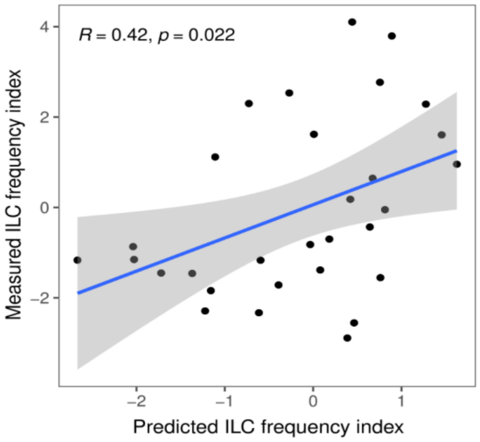

Fig. S27. Predicted vs measured ILC frequency index. Correlation between the measured ILC frequency index and predictions from a single hidden layer neural network based on eicosanoids, HPA-related hormones, and neuroinflammatory factors.

As visible in Fig. S28, the input-output relation was similar to the one obtained on ILC activations, with strongest activations at intermediate eicosanoids, high HPA-related hormones' levels.

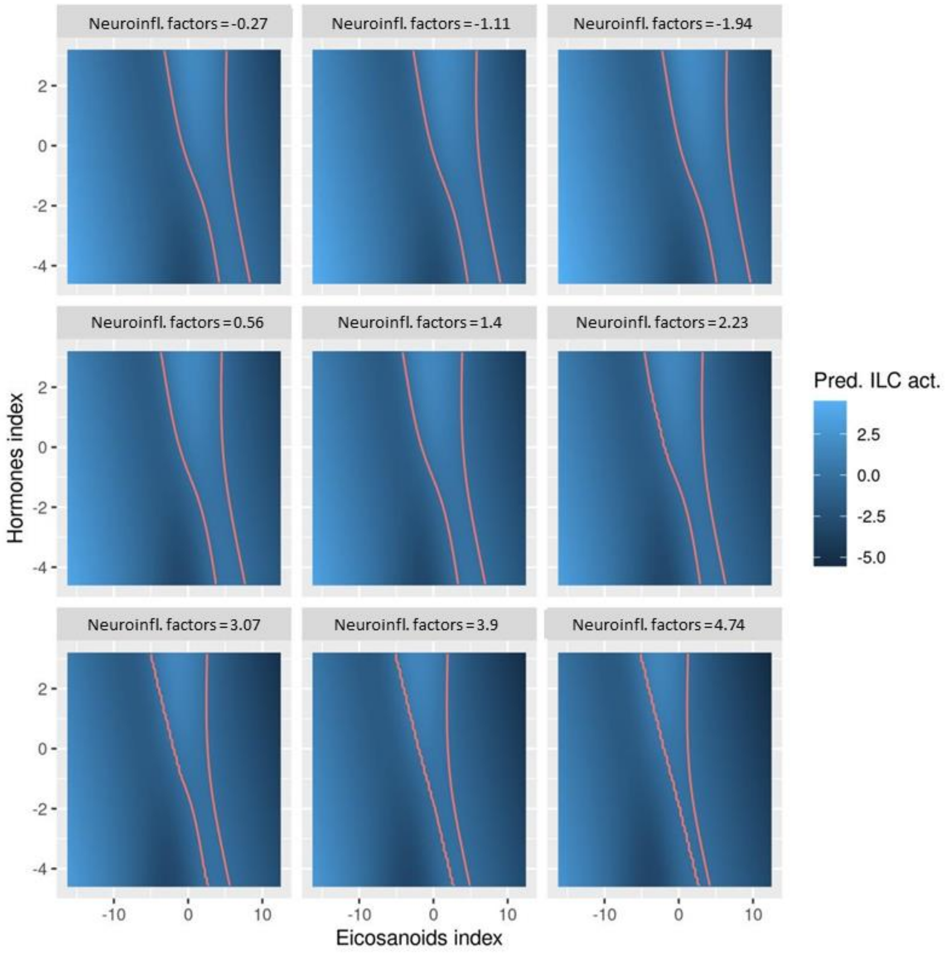

Fig. S28. Complete input-output relation of the ILC frequency network. Input-output relation of the network trained on ILC frequencies, as a function of eicosanoids and HPA related hormones, with each subplot representing a different level of neuroinflammatory factors.

Supplementary References

1. Mehrabian, A., and Ksionzky, S. (1971). Factors of interpersonal behavior and judgment in social groups. Psychol Rep *28*, 483-492. 10.2466/pr0.1971.28.2.483.
2. Lehmann, D., and Skrandies, W. (1980). Reference-free identification of components of checkerboard-evoked multichannel potential fields. Electroencephalogr Clin Neurophysiol *48*, 609-621. 10.1016/0013-4694(80)90419-8.
3. Bernasconi, F., Noel, J.P., Park, H.D., Faivre, N., Seeck, M., Spinelli, L., Schaller, K., Blanke, O., and Serino, A. (2018). Audio-Tactile and Peripersonal Space Processing Around the Trunk in Human Parietal and Temporal Cortex: An Intracranial EEG Study. Cereb Cortex *28*, 3385-3397. 10.1093/cercor/bhy156.
4. Quatrini, L., Wieduwild, E., Escaliere, B., Filtjens, J., Chasson, L., Laprie, C., Vivier, E., and Ugolini, S. (2018). Endogenous glucocorticoids control host resistance to viral infection through the tissue-specific regulation of PD-1 expression on NK cells. Nat Immunol *19*, 954-962. 10.1038/s41590-018-0185-0.
5. Muscari, I., Fierabracci, A., Adorisio, S., Moretti, M., Cannarile, L., Thi Minh Hong, V., Ayroldi, E., and Delfino, D.V. (2022). Glucocorticoids and natural killer cells: A suppressive relationship. Biochem Pharmacol *198*, 114930. 10.1016/j.bcp.2022.114930.
6. Nekrasova, I., Glebezdina, N., Maslennikova, I., Danchenko, I., and Shirshev, S. (2024). Estriol and commensal microflora strains regulate innate lymphoid cells functional activity in multiple sclerosis. Mult Scler Relat Disord *83*, 105453. 10.1016/j.msard.2024.105453.
7. Gorica, E., and Calderone, V. (2022). Arachidonic Acid Derivatives and Neuroinflammation. CNS Neurol Disord Drug Targets *21*, 118-129. 10.2174/1871527320666210208130412.
8. McKinnon, K.M. (2018). Flow Cytometry: An Overview. Curr Protoc Immunol *120*, 5 1 1-5 1 11. 10.1002/cpim.40.
9. Greenwald, A.G., McGhee, D.E., and Schwartz, J.L. (1998). Measuring individual differences in implicit cognition: the implicit association test. J Pers Soc Psychol *74*, 1464-1480. 10.1037//0022-3514.74.6.1464.
10. Greenwald, A.G., Nosek, B.A., and Banaji, M.R. (2003). Understanding and using the implicit association test: I. An improved scoring algorithm. J Pers Soc Psychol *85*, 197-216. 10.1037/0022-3514.85.2.197.
11. Serino, A., Noel, J.P., Galli, G., Canzoneri, E., Marmaroli, P., Lissek, H., and Blanke, O. (2015). Body part-centered and full body-centered peripersonal space representations. Sci Rep *5*, 18603. 10.1038/srep18603.
12. Serino, S., Trabanelli, S., Jandus, C., Fellrath, J., Grivaz, P., Paladino, M.P., and Serino, A. (2021). Sharpening of peripersonal space during the COVID-19 pandemic. Curr Biol *31*, R889-R890. 10.1016/j.cub.2021.06.001.
13. Ellena, G., Battaglia, S., and Ladavas, E. (2020). The spatial effect of fearful faces in the autonomic response. Exp Brain Res *238*, 2009-2018. 10.1007/s00221-020-05829-4.
14. Graziano, M.S., and Cooke, D.F. (2006). Parieto-frontal interactions, personal space, and defensive behavior. Neuropsychologia *44*, 2621-2635. 10.1016/j.neuropsychologia.2005.09.011.
15. Noel, J.P., Chatelle, C., Perdikis, S., Johr, J., Lopes Da Silva, M., Ryvlin, P., De Lucia, M., Millan, J.D.R., Diserens, K., and Serino, A. (2019). Peri-personal space encoding in patients with disorders of consciousness and cognitive-motor dissociation. Neuroimage Clin *24*, 101940. 10.1016/j.nicl.2019.101940.
16. Ronga, I., Galigani, M., Bruno, V., Noel, J.P., Gazzin, A., Perathoner, C., Serino, A., and Garbarini, F. (2021). Spatial tuning of electrophysiological responses to multisensory stimuli

reveals a primitive coding of the body boundaries in newborns. Proc Natl Acad Sci U S A *118*. 10.1073/pnas.2024548118.
17. Murray, M.M., Brunet, D., and Michel, C.M. (2008). Topographic ERP analyses: a step-by-step tutorial review. Brain Topogr *20*, 249-264. 10.1007/s10548-008-0054-5.
