# Supplementary material for: Neural anticipation of virtual infection triggers an immune response
Source: Nat Neurosci. 2025 Jul 28;28(9):1968–77. doi: 10.1038/s41593-025-02008-y (PMC12411218; doi:10.1038/s41593-025-02008-y)
Supplement: Supplementary file 2 — Reporting Summary [file 41593_2025_2008_MOESM2_ESM.pdf]

nature portfolio

Corresponding author(s): Andrea Serino
Camilla Jandus

Last updated by author(s): May 16, 2025

Reporting Summary

Nature Portfolio wishes to improve the reproducibility of the work that we publish. This form provides structure for consistency and transparency in reporting. For further information on Nature Portfolio policies, see our Editorial Policies and the Editorial Policy Checklist.

Statistics

For all statistical analyses, confirm that the following items are present in the figure legend, table legend, main text, or Methods section.

n/a	Confirmed	
☐	☒	The exact sample size (*n*) for each experimental group/condition, given as a discrete number and unit of measurement
☐	☒	A statement on whether measurements were taken from distinct samples or whether the same sample was measured repeatedly
☐	☒	The statistical test(s) used AND whether they are one- or two-sided<br>*Only common tests should be described solely by name; describe more complex techniques in the Methods section.*
☐	☒	A description of all covariates tested
☐	☒	A description of any assumptions or corrections, such as tests of normality and adjustment for multiple comparisons
☐	☒	A full description of the statistical parameters including central tendency (e.g. means) or other basic estimates (e.g. regression coefficient) AND variation (e.g. standard deviation) or associated estimates of uncertainty (e.g. confidence intervals)
☐	☒	For null hypothesis testing, the test statistic (e.g. *F*, *t*, *r*) with confidence intervals, effect sizes, degrees of freedom and *P* value noted<br>*Give P values as exact values whenever suitable.*
☒	☐	For Bayesian analysis, information on the choice of priors and Markov chain Monte Carlo settings
☐	☒	For hierarchical and complex designs, identification of the appropriate level for tests and full reporting of outcomes
☐	☒	Estimates of effect sizes (e.g. Cohen's *d*, Pearson's *r*), indicating how they were calculated

Our web collection on statistics for biologists contains articles on many of the points above.

Software and code

Policy information about availability of computer code

Data collection	Custom Python v3.7 code for ExpyVR toolbox v1.0 (https://www.epfl.ch/labs/lnco/research/expyvr/)<br>BD FACSDiva v8.0.2
Data analysis	GraphPad Prism Version 10.4.1<br>FlowJo software v10.7.1 and v10.8.1 (TreeStar)<br>SPM12 with MATLAB version 2021a<br>Connectome mapper 3 v3.1.0<br>Fieldtrip v20171231 with MATLAB version 2017a<br>EEGLAB v14 with MATLAB version 2017a<br>Cartool v3.12<br>R version v4.2.1

For manuscripts utilizing custom algorithms or software that are central to the research but not yet described in published literature, software must be made available to editors and reviewers. We strongly encourage code deposition in a community repository (e.g. GitHub). See the Nature Portfolio guidelines for submitting code & software for further information.

Data

Policy information about availability of data

All manuscripts must include a data availability statement. This statement should provide the following information, where applicable:

- Accession codes, unique identifiers, or web links for publicly available datasets
- A description of any restrictions on data availability
- For clinical datasets or third party data, please ensure that the statement adheres to our policy

All data are available in the main text or the supplementary materials. Correspondence and material requests should be addressed to camilla.jandus@unige.ch and andrea.serino@unil.ch.

Research involving human participants, their data, or biological material

Policy information about studies with human participants or human data. See also policy information about sex, gender (identity/presentation), and sexual orientation and race, ethnicity and racism.

Reporting on sex and gender	Only sex was taken into consideration and was determined by self-reporting. The answers were collected on subject-coded questionnaires. A total of 248 subjects participated in the study. The different experimental cohorts were age and sex matched (total of 132 females; participants mean age 26.8 years, range 18-49).
Reporting on race, ethnicity, or other socially relevant groupings	Race was collected by self-reporting in the subject-coded questionnaires.<br>This parameter was not used to assign the subjects to the different cohorts and was not taken into consideration during data analysis. It can be retrieved upon request.
Population characteristics	individual disgust scores were used as covariate for fMRI analyses.
Recruitment	All participants were recruited through the participant management software “Sona-Systems”. No self-selection bias was introduced. Participants were only required to be healthy and with an age comprised between 18 and 50. These 2 criteria could limit the generalizability of the results to younger, older or unhealthy people
Ethics oversight	Ethical committee approval “Commission cantonale d'éthique de la recherche sur l'être humain” in Vaud, Switzerland (Project-ID 201701588).

Note that full information on the approval of the study protocol must also be provided in the manuscript.

Field-specific reporting

Please select the one below that is the best fit for your research. If you are not sure, read the appropriate sections before making your selection.

☒ Life sciences ☐ Behavioural & social sciences ☐ Ecological, evolutionary & environmental sciences

For a reference copy of the document with all sections, see nature.com/documents/nr-reporting-summary-flat.pdf

Life sciences study design

All studies must disclose on these points even when the disclosure is negative.

Sample size	Given the absence of previous studies assessing immunological responses to virtual threats, we first calculated the required sample size for the behavioural multisensory experiment. Based on previous experiments [1,2], an averaged effect size f = 0.403 has been calculated. Thus, we originally estimated, for immunomonitoring, a sample size of 15 participants per group, with a desired power of 0.95 (1 – β) on within-group comparisons, via G*Power 3.1 software. The sample size for the other experiments was then established accordingly.<br>For the replication experiment, based on the effect size of the data presented in the manuscript of the original cohorts (neural vs. infection cohorts: Cohen’s d=1.114 for ILCs frequency and d=1.116 for ILCs activation), the sample size with a p-value <.05 and a power of .80 was determined as 14 participants per cohorts. To minimize the risk of dropouts and data loss, we enrolled and analysed 16 participants per group.<br><br>1) Pellencin, E., Paladino, M.P., Herbelin, B., and Serino, A. (2018). Social perception of others shapes one's own multisensory peripersonal space. Cortex 104, 163-179. 10.1016/j.cortex.2017.08.033.<br>2) Serino, A., Noel, J.P., Galli, G., Canzoneri, E., Marmaroli, P., Lissek, H., and Blanke, O. (2015). Body part-centered and full body-centered peripersonal space representations. Sci Rep 5, 18603. 10.1038/srep18603.
Data exclusions	No data were excluded.
Replication	To verify the reproducibility of the experimental findings, we performed a replication experiment for the immuno-related findings in a different city (Geneva, Switzerland instead of Lausanne, Switzerland), several years later (2024 instead of 2018), on a different flow cytometer (BD LSRFortessa™ instead of BD LSR SORP™) and with different experimenters, in comparison to what was done in the first data collection, while maintaining the same paradigm, antibody clones and fluorochrome conjugations. The replication attempt was successful.<br>As in the first data collection, all the subjects were tested in the morning from 8.30 a.m. to 12.30 a.m. and the 2 experimental cohorts (neutral

vs infection cohort) were age and sex matched.
All other experiments were performed independently (distinct biological replicates) and without replication.

Randomization — The allocation was random.

Blinding — Group allocation was blinded for data collection and analysis.

Reporting for specific materials, systems and methods

We require information from authors about some types of materials, experimental systems and methods used in many studies. Here, indicate whether each material, system or method listed is relevant to your study. If you are not sure if a list item applies to your research, read the appropriate section before selecting a response.

Materials & experimental systems

n/a	Involved in the study	
☐	☒	Antibodies
☒	☐	Eukaryotic cell lines
☒	☐	Palaeontology and archaeology
☒	☐	Animals and other organisms
☒	☐	Clinical data
☒	☐	Dual use research of concern
☒	☐	Plants

Methods

n/a	Involved in the study	
☒	☐	ChIP-seq
☐	☒	Flow cytometry
☐	☒	MRI-based neuroimaging

Antibodies

Antibodies used — FITC-conjugated lineage markers: anti-human CD3 (UCHT1, Beckman Coulter (BC) cat. 6604623, dilution 1:200), anti-human CD4 (SFCI12T4D11, BC cat. 6602393, dilution 1:200), anti-human CD8 (MEM- 31, Immunotools cat. 21270083, dilution 1:100), anti-human CD14 (RMO52, BC cat. B36297, dilution 1:200), anti-human CD15 (80H5, BC cat. B36298, dilution 1:50), anti-human CD19 (J3-119, BC cat. A07768, dilution 1:100), anti-human CD20 (2H7, Biolegend cat.980202, dilution 1:400), anti-human CD33 (HIM3-4, Biolegend cat.303304, dilution 1:400), anti-human CD34 (561, Biolegend cat.343604, dilution 1:100), antihuman CD203c (E-NPP3, Biolegend cat. 324614, dilution 1:25), anti-human FcεRIα (AER-37, Biolegend cat. 334608, dilution 1:200). Additionally, we used: APC/Cyanine7 anti-human CD27 (M-T271, Biolegend cat. 356424, dilution 1:50), Brilliant Violet 605 antihuman CD117 (cKit) (104D2, Biolegend cat. 313218, dilution 1:200), Brilliant Violet 421 anti-human CRTH2 (CD294) (BM16, Biolegend cat. 350112, dilution 1:200), PerCP/Cy5.5 anti-human CD335 (NKp46) (9E2, Biolegend, cat. 331920, dilution 1:50), PE antihuman CD337 (NKp30) (P30-15, Biolegend cat. 325208, dilution 1:100), PE/Dazzle 594 anti-human HLA-DR (L243, Biolegend cat.307654, dilution 1:200), PE/Cy7 anti-human KLRG1 (14C2A07, Biolegend cat.368614, dilution 1:200), APC anti-human CD336 (NKp44) (P44-8, Biolegend cat. 325110, dilution 1:100), Alexa Fluor 700 anti-human CD16 (3G8, Biolegend cat. 302026, dilution 1:100), Brilliant Violet 510 anti-human CD25 (BC96, Biolegend cat.302640, dilution 1:100), Brilliant Violet 650 anti-human CD69 (FN50, Biolegend cat. 310934, dilution 1:200), Brilliant Violet 711 anti-human CD279 (PD1) (NAT105, Biolegend cat.367428, dilution 1:50), Brilliant Violet 785 anti-human CD127 (IL-7Rα) (A019D5, Biolegend cat. 351330, dilution 1:200), BUV737 anti-human CD56 (NCAM16.2, BD Biosciences cat. 612767, dilution 1:100).

Validation — All antibodies have been titrated on the same flow cytometer used to run the experiment on human samples containing a positive and a negative population using 6 different dilutions, i.e., 1:25, 1:50, 1:100, 1:200, 1:400, 1:800. The best titration considered as the antibody dilution allowing the best separation between the positive and the negative populations, without affecting the fluorescence intensity of the negative population,have been used to stain the samples of this study. All the antibodies used for the analysis are commercially available and are validated by the manufacturing companies (BC, Immunotools, Biolegend and BD) on either human peripheral blood mononuclear cells or cell lines transfected with the appropriate target.

Plants

Seed stocks — N/A

Novel plant genotypes — N/A

Authentication — N/A

Flow Cytometry

Plots

Confirm that:

☒ The axis labels state the marker and fluorochrome used (e.g. CD4-FITC).

☒ The axis scales are clearly visible. Include numbers along axes only for bottom left plot of group (a 'group' is an analysis of identical markers).

☒ All plots are contour plots with outliers or pseudocolor plots.

☒ A numerical value for number of cells or percentage (with statistics) is provided.

Methodology

Sample preparation	Isolated PBMCs were immediately stained for 20 minutes at room temperature in sorting buffer (PBS, 50 µM EDTA, 0.2% BSA) with the proper antibody mix. DAPI was added to each sample immediately before acquisition.
Instrument	Samples were acquired on a LSR SORP™ flow cytometer (BD) and on a LSRFortessa™ (BD; replication).
Software	BD FACSDiva was used to collect the data. FlowJo software_v10.8.1 (TreeStar) was used to analyse the data.
Cell population abundance	No post-sort fractions were used in this study.
Gating strategy	NKs and ILCs were identified in the FSClowSSClow lymphocyte gate. After doublets' exclusion, living cells were considered as DAPI negative. From the lineage negative (Lin-) living lymphocytes, according to the expression of CD16 and CD56, NKbright (CD56br) were identified as CD56brightCD16-, NKdim (CD56dim) as CD56dimCD16+ and pre-innate lymphoid cells (preILC) as CD56-CD16-. From the preILC, total ILCs (ILCs) were identified as Lin-CD127+ cells. According to the expression of CRTH2 and cKit, ILC1s were gated as CRTH2-cKit-, ILC2s as CRTH2+cKit+/- and ILCPs as CRTH2-cKit+.

☒ Tick this box to confirm that a figure exemplifying the gating strategy is provided in the Supplementary Information.

Magnetic resonance imaging

Experimental design

Design type	task and block design
Design specifications	4 task runs, 47 trials per run, trial duration 2.5 + variable ISI (1.5 - 2.5)
Behavioral performance measures	no behavioral performance measured during MRI acquisitions

Acquisition

Imaging type(s)	functional and structural
Field strength	3T
Sequence & imaging parameters	gradient echo planar imaging (EPI) sequence over the whole brain with TR: 1000 ms; TE: 32 ms; slice thickness: 2 mm; 66 axial slices; in-plane resolution: 2×2 mm2; multi-slice acceleration factor: 6
Area of acquisition	whole brain

Diffusion MRI ☐ Used ☒ Not used

Preprocessing

Preprocessing software	SPM12
Normalization	normalization to MNI space with tissue probabilty maps in spm12
Normalization template	MNI305
Noise and artifact removal	motions parameters
Volume censoring	no volume censoring

Statistical modeling & inference

Model type and settings: mass univariate with random effects as implemented in SPM12

Effect(s) tested: one sample and two sample t-tests on first level contrasts

Specify type of analysis: ☒ Whole brain ☐ ROI-based ☐ Both

Statistic type for inference (See Eklund et al. 2016): FWE cluster-level correction (FWE: $p<0.05$, cluster-defining threshold: $p<0.005$)

Correction: FWE at $p<0.05$

Models & analysis

n/a	Involved in the study
☐	☒ Functional and/or effective connectivity
☒	☐ Graph analysis
☒	☐ Multivariate modeling or predictive analysis

Functional and/or effective connectivity: Dynamical Causal Modeling with Parametric Empirical Bayes framework
